# Supplementary material for: Transcriptome Analysis Reveals Vimentin-Induced Disruption of Cell–Cell Associations Augments Breast Cancer Cell Migration
Source: Cells. 2022 Dec 13;11(24):4035. doi: 10.3390/cells11244035 (PMC9776984; doi:10.3390/cells11244035)
Supplement: Supplementary file 1 [file cells-11-04035-s001.zip › DEGs and enrichment analysis.zip!/DEGs and enrichment analysis/WT_VIMvsVector KEGG/GSEA/WT_VIM/RIBOSOME/RIBOSOME(HSA03010).html]

Details for gene set RIBOSOME(HSA03010)[GSEA]

|  || Dataset | fpkm.sample |
| Phenotype | sample.cls |
| Upregulated in class | WT\_VIM |
| GeneSet | RIBOSOME(HSA03010) |
| Enrichment Score (ES) | 0.6525345 |
| Normalized Enrichment Score (NES) | 1.2003291 |
| Nominal p-value | 0.0 |
| FDR q-value | 0.23785724 |
| FWER p-Value | 0.335 |
Table: GSEA Results Summary

  

Fig 1: Enrichment plot: RIBOSOME(HSA03010)      
 Profile of the Running ES Score & Positions of GeneSet Members on the Rank Ordered List

  

| PROBE | DESCRIPTION (from dataset) | GENE SYMBOL | GENE\_TITLE | RANK IN GENE LIST | RANK METRIC SCORE | RUNNING ES | CORE ENRICHMENT || 1 | ENSG00000243904 | RPSAP5 |  |  | 17 | Infinity | -0.0003 | Yes |
| 2 | ENSG00000060303 | RPS17P5 |  |  | 40 | Infinity | -0.0007 | Yes |
| 3 | ENSG00000225893 | AL136231.1 |  |  | 65 | Infinity | -0.0011 | Yes |
| 4 | ENSG00000232439 | RPL18AP7 |  |  | 141 | Infinity | -0.0024 | Yes |
| 5 | ENSG00000244451 | RPL34P21 |  |  | 166 | Infinity | -0.0028 | Yes |
| 6 | ENSG00000213399 | AC022210.1 |  |  | 221 | Infinity | -0.0038 | Yes |
| 7 | ENSG00000260615 | RPL23AP97 |  |  | 240 | Infinity | -0.0041 | Yes |
| 8 | ENSG00000224011 | AC110602.1 |  |  | 273 | Infinity | -0.0046 | Yes |
| 9 | ENSG00000244582 | RPL21P120 |  |  | 276 | Infinity | -0.0046 | Yes |
| 10 | ENSG00000233416 | AC012065.2 |  |  | 303 | Infinity | -0.0051 | Yes |
| 11 | ENSG00000235916 | AC233279.1 |  |  | 327 | Infinity | -0.0055 | Yes |
| 12 | ENSG00000224545 | AC008264.1 |  |  | 357 | Infinity | -0.0060 | Yes |
| 13 | ENSG00000244691 | RPL10AP1 |  |  | 427 | Infinity | -0.0072 | Yes |
| 14 | ENSG00000213663 | AC010409.1 |  |  | 450 | Infinity | -0.0076 | Yes |
| 15 | ENSG00000215236 | RPL7P33 |  |  | 455 | Infinity | -0.0076 | Yes |
| 16 | ENSG00000236762 | RPL19P16 |  |  | 475 | Infinity | -0.0080 | Yes |
| 17 | ENSG00000225416 | AC104843.1 |  |  | 511 | Infinity | -0.0086 | Yes |
| 18 | ENSG00000240366 | RPL36AP45 |  |  | 522 | Infinity | -0.0087 | Yes |
| 19 | ENSG00000242888 | AL133368.1 |  |  | 531 | Infinity | -0.0089 | Yes |
| 20 | ENSG00000262141 | AC040162.2 |  |  | 551 | Infinity | -0.0092 | Yes |
| 21 | ENSG00000215840 | AL592295.1 |  |  | 585 | Infinity | -0.0098 | Yes |
| 22 | ENSG00000255296 | AP002490.2 |  |  | 602 | Infinity | -0.0100 | Yes |
| 23 | ENSG00000229503 | AC092155.2 |  |  | 626 | Infinity | -0.0104 | Yes |
| 24 | ENSG00000220105 | AL353151.1 |  |  | 628 | Infinity | -0.0105 | Yes |
| 25 | ENSG00000243687 | AC079594.1 |  |  | 637 | Infinity | -0.0106 | Yes |
| 26 | ENSG00000227836 | AC008850.1 |  |  | 653 | Infinity | -0.0108 | Yes |
| 27 | ENSG00000213779 | AC107956.2 |  |  | 657 | Infinity | -0.0109 | Yes |
| 28 | ENSG00000232142 | RPS25P9 |  |  | 670 | Infinity | -0.0111 | Yes |
| 29 | ENSG00000240950 | AC021074.1 |  |  | 693 | Infinity | -0.0115 | Yes |
| 30 | ENSG00000231241 | RPS3AP3 |  |  | 715 | Infinity | -0.0118 | Yes |
| 31 | ENSG00000215520 | RPS4XP4 |  |  | 727 | Infinity | -0.0120 | Yes |
| 32 | ENSG00000258204 | AC008126.1 |  |  | 728 | Infinity | -0.0120 | Yes |
| 33 | ENSG00000224340 | AL590639.1 |  |  | 732 | Infinity | -0.0121 | Yes |
| 34 | ENSG00000241255 | AL136126.1 |  |  | 762 | Infinity | -0.0126 | Yes |
| 35 | ENSG00000224415 | AC007683.1 |  |  | 843 | Infinity | -0.0140 | Yes |
| 36 | ENSG00000233541 | RPL31P47 |  |  | 848 | Infinity | -0.0140 | Yes |
| 37 | ENSG00000215184 | RPS12P16 |  |  | 849 | Infinity | -0.0140 | Yes |
| 38 | ENSG00000234135 | RPL23AP83 |  |  | 851 | Infinity | -0.0140 | Yes |
| 39 | ENSG00000228195 | RPL5P27 |  |  | 866 | Infinity | -0.0143 | Yes |
| 40 | ENSG00000244217 | RPS4XP10 |  |  | 918 | Infinity | -0.0151 | Yes |
| 41 | ENSG00000234982 | AL441963.1 |  |  | 954 | Infinity | -0.0158 | Yes |
| 42 | ENSG00000234981 | AC244034.1 |  |  | 955 | Infinity | -0.0157 | Yes |
| 43 | ENSG00000233668 | AL353662.2 |  |  | 956 | Infinity | -0.0157 | Yes |
| 44 | ENSG00000241597 | AC022121.1 |  |  | 970 | Infinity | -0.0160 | Yes |
| 45 | ENSG00000236317 | AC104333.2 |  |  | 987 | Infinity | -0.0162 | Yes |
| 46 | ENSG00000213942 | AC005840.1 |  |  | 1010 | Infinity | -0.0166 | Yes |
| 47 | ENSG00000231995 | AL590399.4 |  |  | 1039 | Infinity | -0.0171 | Yes |
| 48 | ENSG00000213302 | AC015983.1 |  |  | 1053 | Infinity | -0.0173 | Yes |
| 49 | ENSG00000225272 | AL451074.3 |  |  | 1055 | Infinity | -0.0173 | Yes |
| 50 | ENSG00000214016 | RPSAP61 |  |  | 1065 | Infinity | -0.0175 | Yes |
| 51 | ENSG00000237077 | AC105399.1 |  |  | 1123 | Infinity | -0.0185 | Yes |
| 52 | ENSG00000242262 | AC092597.1 |  |  | 1124 | Infinity | -0.0185 | Yes |
| 53 | ENSG00000244245 | AC133134.1 |  |  | 1182 | Infinity | -0.0195 | Yes |
| 54 | ENSG00000235315 | RPL23AP69 |  |  | 1183 | Infinity | -0.0195 | Yes |
| 55 | ENSG00000213891 | RPL3P6 |  |  | 1215 | Infinity | -0.0200 | Yes |
| 56 | ENSG00000225854 | AL451139.1 |  |  | 1216 | Infinity | -0.0200 | Yes |
| 57 | ENSG00000254522 | AP001767.1 |  |  | 1219 | Infinity | -0.0200 | Yes |
| 58 | ENSG00000261303 | AC022748.2 |  |  | 1259 | Infinity | -0.0207 | Yes |
| 59 | ENSG00000230958 | AC093166.3 |  |  | 1262 | Infinity | -0.0207 | Yes |
| 60 | ENSG00000232176 | AL161909.1 |  |  | 1269 | Infinity | -0.0208 | Yes |
| 61 | ENSG00000240828 | RPL21P4 |  |  | 1272 | Infinity | -0.0208 | Yes |
| 62 | ENSG00000236848 | RPL23AP95 |  |  | 1290 | Infinity | -0.0211 | Yes |
| 63 | ENSG00000257531 | AC008147.3 |  |  | 1316 | Infinity | -0.0216 | Yes |
| 64 | ENSG00000217644 | AL355864.1 |  |  | 1326 | Infinity | -0.0217 | Yes |
| 65 | ENSG00000217527 | RPS16P5 |  |  | 1363 | Infinity | -0.0223 | Yes |
| 66 | ENSG00000242595 | RPL26P26 |  |  | 1423 | Infinity | -0.0234 | Yes |
| 67 | ENSG00000225616 | AL138785.1 |  |  | 1443 | Infinity | -0.0237 | Yes |
| 68 | ENSG00000271057 | AC073648.5 |  |  | 1455 | Infinity | -0.0239 | Yes |
| 69 | ENSG00000215467 | RPL27AP |  |  | 1467 | Infinity | -0.0241 | Yes |
| 70 | ENSG00000216316 | AL022722.1 |  |  | 1486 | Infinity | -0.0244 | Yes |
| 71 | ENSG00000196933 | RPS26P11 |  |  | 1564 | Infinity | -0.0257 | Yes |
| 72 | ENSG00000235459 | RPS26P31 |  |  | 1565 | Infinity | -0.0257 | Yes |
| 73 | ENSG00000226299 | RPSAP62 |  |  | 1586 | Infinity | -0.0261 | Yes |
| 74 | ENSG00000248459 | AC083906.1 |  |  | 1624 | Infinity | -0.0267 | Yes |
| 75 | ENSG00000214629 | RPSAP6 |  |  | 1643 | Infinity | -0.0270 | Yes |
| 76 | ENSG00000241400 | AC022215.1 |  |  | 1649 | Infinity | -0.0271 | Yes |
| 77 | ENSG00000214541 | RPS4XP3 |  |  | 1665 | Infinity | -0.0273 | Yes |
| 78 | ENSG00000228375 | RPS20P25 |  |  | 1669 | Infinity | -0.0274 | Yes |
| 79 | ENSG00000234513 | AC073072.2 |  |  | 1689 | Infinity | -0.0277 | Yes |
| 80 | ENSG00000230364 | RPL4P3 |  |  | 1713 | Infinity | -0.0281 | Yes |
| 81 | ENSG00000232897 | AL117329.2 |  |  | 1717 | Infinity | -0.0282 | Yes |
| 82 | ENSG00000250324 | MRPL22P1 |  |  | 1730 | Infinity | -0.0284 | Yes |
| 83 | ENSG00000225770 | RPS29P3 |  |  | 1763 | Infinity | -0.0289 | Yes |
| 84 | ENSG00000244021 | AC093591.1 |  |  | 1794 | Infinity | -0.0294 | Yes |
| 85 | ENSG00000214922 | HLA-F-AS1 |  |  | 1797 | Infinity | -0.0295 | Yes |
| 86 | ENSG00000237389 | AL645634.2 |  |  | 1831 | Infinity | -0.0300 | Yes |
| 87 | ENSG00000176043 | AC007160.1 |  |  | 1856 | Infinity | -0.0304 | Yes |
| 88 | ENSG00000236325 | AC005300.1 |  |  | 1874 | Infinity | -0.0307 | Yes |
| 89 | ENSG00000249148 | AC006445.2 |  |  | 1894 | Infinity | -0.0311 | Yes |
| 90 | ENSG00000230721 | AL049597.1 |  |  | 1929 | Infinity | -0.0317 | Yes |
| 91 | ENSG00000227939 | RPL3P2 |  |  | 1936 | Infinity | -0.0318 | Yes |
| 92 | ENSG00000188873 | RPL10AP2 |  |  | 1948 | Infinity | -0.0319 | Yes |
| 93 | ENSG00000241641 | RPS23P6 |  |  | 1970 | Infinity | -0.0323 | Yes |
| 94 | ENSG00000179131 | AC073335.1 |  |  | 1974 | Infinity | -0.0323 | Yes |
| 95 | ENSG00000219023 | AL033519.2 |  |  | 1980 | Infinity | -0.0324 | Yes |
| 96 | ENSG00000258245 | RPL10P13 |  |  | 1982 | Infinity | -0.0324 | Yes |
| 97 | ENSG00000229585 | RPL21P44 |  |  | 2000 | Infinity | -0.0327 | Yes |
| 98 | ENSG00000240418 | AC020917.1 |  |  | 2005 | Infinity | -0.0328 | Yes |
| 99 | ENSG00000255991 | AC121334.1 |  |  | 2027 | Infinity | -0.0331 | Yes |
| 100 | ENSG00000237668 | RPS15AP38 |  |  | 2069 | Infinity | -0.0339 | Yes |
| 101 | ENSG00000214359 | RPL18P10 |  |  | 2090 | Infinity | -0.0342 | Yes |
| 102 | ENSG00000258640 | RPL21P5 |  |  | 2112 | Infinity | -0.0346 | Yes |
| 103 | ENSG00000235937 | AC008280.1 |  |  | 2128 | Infinity | -0.0348 | Yes |
| 104 | ENSG00000236358 | AL355472.3 |  |  | 2132 | Infinity | -0.0349 | Yes |
| 105 | ENSG00000226617 | RPL21P110 |  |  | 2140 | Infinity | -0.0350 | Yes |
| 106 | ENSG00000213988 | ZNF90 |  |  | 2160 | Infinity | -0.0353 | Yes |
| 107 | ENSG00000259195 | AC021739.1 |  |  | 2190 | Infinity | -0.0358 | Yes |
| 108 | ENSG00000243403 | AC090543.2 |  |  | 2197 | Infinity | -0.0359 | Yes |
| 109 | ENSG00000226080 | AC062028.2 |  |  | 2267 | Infinity | -0.0371 | Yes |
| 110 | ENSG00000256159 | AC022080.3 |  |  | 2313 | Infinity | -0.0379 | Yes |
| 111 | ENSG00000226915 | AC068137.1 |  |  | 2319 | Infinity | -0.0380 | Yes |
| 112 | ENSG00000229721 | AC104115.1 |  |  | 2323 | Infinity | -0.0380 | Yes |
| 113 | ENSG00000241825 | RPL7P42 |  |  | 2344 | Infinity | -0.0384 | Yes |
| 114 | ENSG00000239544 | AL442663.1 |  |  | 2352 | Infinity | -0.0385 | Yes |
| 115 | ENSG00000213035 | RPL23AP80 |  |  | 2385 | Infinity | -0.0390 | Yes |
| 116 | ENSG00000226945 | AC098935.1 |  |  | 2394 | 74.226 | 0.0228 | Yes |
| 117 | ENSG00000235508 | RPS2P7 |  |  | 2408 | 27.013 | 0.0452 | Yes |
| 118 | ENSG00000212829 | RPS26P3 |  |  | 2419 | 21.234 | 0.0627 | Yes |
| 119 | ENSG00000241112 | RPL29P14 |  |  | 2440 | 16.521 | 0.0762 | Yes |
| 120 | novel.658 | - |  |  | 2451 | 15.441 | 0.0889 | Yes |
| 121 | ENSG00000234354 | RPS26P47 |  |  | 2468 | 13.534 | 0.0999 | Yes |
| 122 | ENSG00000250321 | AC079140.2 |  |  | 2480 | 12.495 | 0.1102 | Yes |
| 123 | ENSG00000244073 | RPS4XP6 |  |  | 2481 | 12.414 | 0.1206 | Yes |
| 124 | ENSG00000228007 | AC011933.1 |  |  | 2541 | 9.713 | 0.1276 | Yes |
| 125 | ENSG00000233045 | AC097523.1 |  |  | 2564 | 8.729 | 0.1345 | Yes |
| 126 | ENSG00000216866 | RPS2P55 |  |  | 2565 | 8.694 | 0.1418 | Yes |
| 127 | ENSG00000241352 | AC007688.1 |  |  | 2600 | 8.111 | 0.1480 | Yes |
| 128 | ENSG00000240785 | RPL36AP21 |  |  | 2613 | 7.910 | 0.1544 | Yes |
| 129 | ENSG00000234268 | AP000936.3 |  |  | 2619 | 7.830 | 0.1608 | Yes |
| 130 | ENSG00000241458 | RPL7P19 |  |  | 2624 | 7.734 | 0.1672 | Yes |
| 131 | ENSG00000140986 | RPL3L |  |  | 2637 | 7.594 | 0.1734 | Yes |
| 132 | ENSG00000224114 | AL591846.1 |  |  | 2651 | 7.379 | 0.1793 | Yes |
| 133 | ENSG00000219932 | RPL12P8 |  |  | 2680 | 7.078 | 0.1847 | Yes |
| 134 | ENSG00000224261 | RPSAP18 |  |  | 2697 | 6.875 | 0.1902 | Yes |
| 135 | ENSG00000228205 | AC131235.1 |  |  | 2711 | 6.766 | 0.1956 | Yes |
| 136 | ENSG00000234648 | AL162151.2 |  |  | 2735 | 6.525 | 0.2006 | Yes |
| 137 | ENSG00000241741 | RPL7AP30 |  |  | 2749 | 6.459 | 0.2058 | Yes |
| 138 | ENSG00000240311 | AC004224.1 |  |  | 2769 | 6.269 | 0.2107 | Yes |
| 139 | ENSG00000231369 | Z97353.1 |  |  | 2784 | 6.149 | 0.2156 | Yes |
| 140 | ENSG00000239246 | AC008026.1 |  |  | 2788 | 6.079 | 0.2206 | Yes |
| 141 | ENSG00000227331 | AC005042.1 |  |  | 2790 | 6.079 | 0.2257 | Yes |
| 142 | ENSG00000183298 | RPSAP19 |  |  | 2812 | 5.940 | 0.2303 | Yes |
| 143 | ENSG00000243181 | AC087343.1 |  |  | 2818 | 5.914 | 0.2351 | Yes |
| 144 | ENSG00000213609 | RPL7AP50 |  |  | 2844 | 5.740 | 0.2395 | Yes |
| 145 | ENSG00000234335 | RPS4XP11 |  |  | 2860 | 5.624 | 0.2439 | Yes |
| 146 | ENSG00000224683 | RPL36AP29 |  |  | 2877 | 5.547 | 0.2483 | Yes |
| 147 | ENSG00000234004 | AC092017.2 |  |  | 2885 | 5.474 | 0.2527 | Yes |
| 148 | ENSG00000239218 | RPS20P22 |  |  | 2951 | 5.117 | 0.2559 | Yes |
| 149 | ENSG00000217716 | RPS10P3 |  |  | 2966 | 5.045 | 0.2598 | Yes |
| 150 | ENSG00000230291 | AC078817.1 |  |  | 2972 | 5.003 | 0.2639 | Yes |
| 151 | ENSG00000239797 | RPL21P39 |  |  | 3014 | 4.827 | 0.2672 | Yes |
| 152 | ENSG00000230979 | AC079250.1 |  |  | 3016 | 4.803 | 0.2712 | Yes |
| 153 | ENSG00000233406 | AL162430.1 |  |  | 3068 | 4.628 | 0.2742 | Yes |
| 154 | ENSG00000250327 | RPSAP70 |  |  | 3074 | 4.628 | 0.2780 | Yes |
| 155 | ENSG00000213529 | AL359073.1 |  |  | 3093 | 4.618 | 0.2815 | Yes |
| 156 | ENSG00000243680 | RPL37P23 |  |  | 3122 | 4.453 | 0.2847 | Yes |
| 157 | ENSG00000231409 | AC018868.1 |  |  | 3124 | 4.453 | 0.2884 | Yes |
| 158 | ENSG00000226396 | AL031727.1 |  |  | 3134 | 4.425 | 0.2920 | Yes |
| 159 | ENSG00000213280 | AC090114.1 |  |  | 3145 | 4.394 | 0.2955 | Yes |
| 160 | ENSG00000215349 | MRPL3P1 |  |  | 3151 | 4.343 | 0.2990 | Yes |
| 161 | ENSG00000250318 | AC003072.1 |  |  | 3233 | 4.156 | 0.3011 | Yes |
| 162 | ENSG00000243824 | RPL12P6 |  |  | 3324 | 3.955 | 0.3028 | Yes |
| 163 | novel.634 | - |  |  | 3340 | 3.942 | 0.3058 | Yes |
| 164 | ENSG00000231414 | AC016700.2 |  |  | 3354 | 3.867 | 0.3088 | Yes |
| 165 | ENSG00000223803 | RPS20P14 |  |  | 3378 | 3.818 | 0.3116 | Yes |
| 166 | ENSG00000249773 | AC092647.5 |  |  | 3394 | 3.794 | 0.3145 | Yes |
| 167 | ENSG00000244086 | RPS20P35 |  |  | 3395 | 3.794 | 0.3177 | Yes |
| 168 | ENSG00000180211 | FO393411.1 |  |  | 3405 | 3.766 | 0.3207 | Yes |
| 169 | ENSG00000213939 | AC091153.1 |  |  | 3448 | 3.711 | 0.3230 | Yes |
| 170 | ENSG00000140988 | RPS2 |  |  | 3501 | 3.663 | 0.3252 | Yes |
| 171 | ENSG00000229605 | RPL21P93 |  |  | 3507 | 3.656 | 0.3281 | Yes |
| 172 | ENSG00000234040 | RPL10P12 |  |  | 3532 | 3.632 | 0.3307 | Yes |
| 173 | ENSG00000171858 | RPS21 |  |  | 3544 | 3.623 | 0.3336 | Yes |
| 174 | ENSG00000213304 | AC008481.2 |  |  | 3559 | 3.581 | 0.3363 | Yes |
| 175 | ENSG00000230897 | RPS18P12 |  |  | 3581 | 3.525 | 0.3389 | Yes |
| 176 | ENSG00000227097 | RPS28P7 |  |  | 3627 | 3.461 | 0.3410 | Yes |
| 177 | ENSG00000213885 | RPL13AP7 |  |  | 3642 | 3.439 | 0.3436 | Yes |
| 178 | ENSG00000233762 | AC007969.1 |  |  | 3643 | 3.437 | 0.3465 | Yes |
| 179 | ENSG00000244716 | BX679664.3 |  |  | 3673 | 3.389 | 0.3488 | Yes |
| 180 | ENSG00000242445 | RPL7AP11 |  |  | 3703 | 3.364 | 0.3511 | Yes |
| 181 | ENSG00000226243 | RPL37AP1 |  |  | 3719 | 3.349 | 0.3537 | Yes |
| 182 | ENSG00000234742 | AC144530.1 |  |  | 3723 | 3.345 | 0.3564 | Yes |
| 183 | ENSG00000241680 | RPL31P49 |  |  | 3762 | 3.310 | 0.3585 | Yes |
| 184 | ENSG00000224892 | RPS4XP16 |  |  | 3767 | 3.308 | 0.3612 | Yes |
| 185 | ENSG00000241081 | RPL22P2 |  |  | 3806 | 3.273 | 0.3632 | Yes |
| 186 | ENSG00000205871 | RPS3AP47 |  |  | 3825 | 3.248 | 0.3656 | Yes |
| 187 | ENSG00000184612 | RPL7P26 |  |  | 3847 | 3.212 | 0.3680 | Yes |
| 188 | ENSG00000239470 | AC011979.1 |  |  | 3850 | 3.206 | 0.3706 | Yes |
| 189 | ENSG00000243304 | AC008494.1 |  |  | 3884 | 3.160 | 0.3727 | Yes |
| 190 | ENSG00000228981 | AC097658.1 |  |  | 3948 | 3.088 | 0.3741 | Yes |
| 191 | ENSG00000243964 | RPL23AP65 |  |  | 3958 | 3.077 | 0.3765 | Yes |
| 192 | ENSG00000139239 | RPL14P1 |  |  | 3988 | 3.037 | 0.3786 | Yes |
| 193 | ENSG00000083845 | RPS5 |  |  | 3991 | 3.033 | 0.3811 | Yes |
| 194 | ENSG00000212664 | AC064799.1 |  |  | 4010 | 3.009 | 0.3833 | Yes |
| 195 | ENSG00000231006 | RPL7P32 |  |  | 4025 | 2.991 | 0.3855 | Yes |
| 196 | ENSG00000233190 | RPS24P13 |  |  | 4031 | 2.987 | 0.3879 | Yes |
| 197 | ENSG00000243094 | AC079203.1 |  |  | 4036 | 2.987 | 0.3903 | Yes |
| 198 | ENSG00000236132 | AL022331.1 |  |  | 4037 | 2.987 | 0.3928 | Yes |
| 199 | ENSG00000230702 | AC107081.1 |  |  | 4040 | 2.987 | 0.3953 | Yes |
| 200 | ENSG00000224598 | RPS5P2 |  |  | 4055 | 2.987 | 0.3976 | Yes |
| 201 | ENSG00000229455 | RPS10P18 |  |  | 4065 | 2.987 | 0.3999 | Yes |
| 202 | ENSG00000213757 | AC020898.1 |  |  | 4124 | 2.935 | 0.4013 | Yes |
| 203 | ENSG00000214784 | AC010468.1 |  |  | 4201 | 2.860 | 0.4024 | Yes |
| 204 | ENSG00000232926 | AC000078.1 |  |  | 4223 | 2.842 | 0.4044 | Yes |
| 205 | ENSG00000231333 | RPL34P6 |  |  | 4311 | 2.787 | 0.4052 | Yes |
| 206 | ENSG00000273870 | AL138721.1 |  |  | 4322 | 2.787 | 0.4073 | Yes |
| 207 | ENSG00000240616 | RPS6P25 |  |  | 4330 | 2.782 | 0.4095 | Yes |
| 208 | ENSG00000219747 | AL133260.1 |  |  | 4351 | 2.769 | 0.4115 | Yes |
| 209 | ENSG00000236264 | RPL26P30 |  |  | 4365 | 2.756 | 0.4136 | Yes |
| 210 | ENSG00000217275 | AL031777.1 |  |  | 4382 | 2.742 | 0.4156 | Yes |
| 211 | ENSG00000215030 | RPL13P12 |  |  | 4390 | 2.738 | 0.4177 | Yes |
| 212 | ENSG00000213741 | RPS29 |  |  | 4449 | 2.684 | 0.4190 | Yes |
| 213 | ENSG00000234287 | AC099560.2 |  |  | 4456 | 2.679 | 0.4211 | Yes |
| 214 | ENSG00000175886 | RPL7AP66 |  |  | 4465 | 2.672 | 0.4232 | Yes |
| 215 | ENSG00000239559 | RPL37P2 |  |  | 4559 | 2.637 | 0.4237 | Yes |
| 216 | ENSG00000233487 | RPSAP69 |  |  | 4571 | 2.630 | 0.4257 | Yes |
| 217 | ENSG00000229057 | RPS3AP54 |  |  | 4586 | 2.623 | 0.4277 | Yes |
| 218 | ENSG00000240395 | RPL5P23 |  |  | 4597 | 2.616 | 0.4297 | Yes |
| 219 | ENSG00000189343 | RPS2P46 |  |  | 4624 | 2.599 | 0.4314 | Yes |
| 220 | ENSG00000158042 | MRPL17 |  |  | 4629 | 2.597 | 0.4335 | Yes |
| 221 | ENSG00000161016 | RPL8 |  |  | 4659 | 2.582 | 0.4352 | Yes |
| 222 | ENSG00000240376 | AC010343.1 |  |  | 4661 | 2.581 | 0.4373 | Yes |
| 223 | ENSG00000122140 | MRPS2 |  |  | 4700 | 2.558 | 0.4388 | Yes |
| 224 | ENSG00000227077 | AC107983.1 |  |  | 4813 | 2.481 | 0.4389 | Yes |
| 225 | ENSG00000267355 | RPL9P29 |  |  | 4831 | 2.474 | 0.4406 | Yes |
| 226 | novel.591 | - |  |  | 4848 | 2.469 | 0.4424 | Yes |
| 227 | ENSG00000224988 | AL158207.1 |  |  | 4851 | 2.468 | 0.4444 | Yes |
| 228 | ENSG00000240087 | RPSAP12 |  |  | 4863 | 2.463 | 0.4463 | Yes |
| 229 | ENSG00000213296 | AC005521.1 |  |  | 4882 | 2.460 | 0.4480 | Yes |
| 230 | ENSG00000240435 | RPS12P27 |  |  | 4896 | 2.460 | 0.4499 | Yes |
| 231 | ENSG00000239602 | AC091959.2 |  |  | 4905 | 2.460 | 0.4518 | Yes |
| 232 | ENSG00000105364 | MRPL4 |  |  | 4913 | 2.455 | 0.4537 | Yes |
| 233 | ENSG00000233913 | RPL10P9 |  |  | 4944 | 2.438 | 0.4552 | Yes |
| 234 | ENSG00000242951 | AC007182.2 |  |  | 4965 | 2.423 | 0.4569 | Yes |
| 235 | ENSG00000086504 | MRPL28 |  |  | 4993 | 2.405 | 0.4584 | Yes |
| 236 | ENSG00000241431 | RPL37P6 |  |  | 5068 | 2.368 | 0.4591 | Yes |
| 237 | ENSG00000259441 | MRPL15P1 |  |  | 5146 | 2.322 | 0.4597 | Yes |
| 238 | ENSG00000270781 | AC091133.5 |  |  | 5162 | 2.314 | 0.4614 | Yes |
| 239 | ENSG00000235332 | RPSAP76 |  |  | 5168 | 2.314 | 0.4632 | Yes |
| 240 | ENSG00000253175 | AC023632.1 |  |  | 5195 | 2.314 | 0.4647 | Yes |
| 241 | ENSG00000213432 | RPL17P34 |  |  | 5220 | 2.309 | 0.4662 | Yes |
| 242 | ENSG00000241556 | AC018475.1 |  |  | 5222 | 2.309 | 0.4681 | Yes |
| 243 | ENSG00000262636 | AC099489.2 |  |  | 5225 | 2.309 | 0.4700 | Yes |
| 244 | ENSG00000242083 | RPL7AP31 |  |  | 5233 | 2.308 | 0.4718 | Yes |
| 245 | ENSG00000231851 | AC104655.1 |  |  | 5272 | 2.287 | 0.4730 | Yes |
| 246 | ENSG00000137818 | RPLP1 |  |  | 5298 | 2.276 | 0.4745 | Yes |
| 247 | ENSG00000226360 | RPL10AP6 |  |  | 5316 | 2.269 | 0.4761 | Yes |
| 248 | ENSG00000226525 | RPS7P10 |  |  | 5330 | 2.261 | 0.4778 | Yes |
| 249 | ENSG00000241634 | AC069499.1 |  |  | 5343 | 2.256 | 0.4794 | Yes |
| 250 | ENSG00000112651 | MRPL2 |  |  | 5374 | 2.243 | 0.4808 | Yes |
| 251 | ENSG00000223522 | AC093690.1 |  |  | 5381 | 2.241 | 0.4825 | Yes |
| 252 | ENSG00000217130 | AL139100.1 |  |  | 5400 | 2.233 | 0.4841 | Yes |
| 253 | ENSG00000166902 | MRPL16 |  |  | 5411 | 2.229 | 0.4858 | Yes |
| 254 | ENSG00000226221 | RPL26P19 |  |  | 5418 | 2.225 | 0.4875 | Yes |
| 255 | ENSG00000242299 | AC073861.1 |  |  | 5425 | 2.223 | 0.4893 | Yes |
| 256 | ENSG00000105640 | RPL18A |  |  | 5448 | 2.217 | 0.4907 | Yes |
| 257 | ENSG00000215221 | UBA52P6 |  |  | 5493 | 2.201 | 0.4918 | Yes |
| 258 | ENSG00000230076 | RPL10P6 |  |  | 5506 | 2.196 | 0.4934 | Yes |
| 259 | ENSG00000262814 | MRPL12 |  |  | 5521 | 2.192 | 0.4950 | Yes |
| 260 | ENSG00000174547 | MRPL11 |  |  | 5553 | 2.181 | 0.4963 | Yes |
| 261 | ENSG00000244134 | RPS12P20 |  |  | 5597 | 2.159 | 0.4973 | Yes |
| 262 | ENSG00000224800 | RPS27AP6 |  |  | 5598 | 2.159 | 0.4991 | Yes |
| 263 | ENSG00000242571 | RPL21P11 |  |  | 5606 | 2.159 | 0.5008 | Yes |
| 264 | ENSG00000213228 | RPL12P38 |  |  | 5659 | 2.150 | 0.5017 | Yes |
| 265 | ENSG00000241061 | RPL5P1 |  |  | 5664 | 2.149 | 0.5034 | Yes |
| 266 | ENSG00000188856 | RPSAP47 |  |  | 5708 | 2.135 | 0.5045 | Yes |
| 267 | ENSG00000225513 | AL158824.1 |  |  | 5746 | 2.119 | 0.5056 | Yes |
| 268 | ENSG00000150991 | UBC |  |  | 5815 | 2.092 | 0.5061 | Yes |
| 269 | ENSG00000233927 | RPS28 |  |  | 5826 | 2.087 | 0.5077 | Yes |
| 270 | ENSG00000037241 | RPL26L1 |  |  | 5837 | 2.084 | 0.5093 | Yes |
| 271 | ENSG00000244313 | AC024293.1 |  |  | 5843 | 2.080 | 0.5109 | Yes |
| 272 | ENSG00000232385 | RPS3AP25 |  |  | 5847 | 2.079 | 0.5126 | Yes |
| 273 | ENSG00000232454 | AL138752.1 |  |  | 5904 | 2.061 | 0.5133 | Yes |
| 274 | ENSG00000220749 | RPL21P28 |  |  | 5948 | 2.048 | 0.5143 | Yes |
| 275 | ENSG00000105193 | RPS16 |  |  | 5967 | 2.044 | 0.5157 | Yes |
| 276 | ENSG00000229119 | AC026403.1 |  |  | 5993 | 2.035 | 0.5169 | Yes |
| 277 | ENSG00000240342 | RPS2P5 |  |  | 6005 | 2.033 | 0.5184 | Yes |
| 278 | ENSG00000227008 | AL009174.1 |  |  | 6011 | 2.031 | 0.5200 | Yes |
| 279 | ENSG00000243979 | AC087752.1 |  |  | 6017 | 2.029 | 0.5216 | Yes |
| 280 | ENSG00000130255 | RPL36 |  |  | 6038 | 2.023 | 0.5230 | Yes |
| 281 | ENSG00000241030 | RPL29P24 |  |  | 6083 | 2.008 | 0.5239 | Yes |
| 282 | ENSG00000213442 | RPL18AP3 |  |  | 6099 | 2.004 | 0.5253 | Yes |
| 283 | ENSG00000167526 | RPL13 |  |  | 6122 | 1.998 | 0.5266 | Yes |
| 284 | ENSG00000135390 | ATP5MC2 |  |  | 6132 | 1.995 | 0.5281 | Yes |
| 285 | ENSG00000115268 | RPS15 |  |  | 6144 | 1.993 | 0.5296 | Yes |
| 286 | ENSG00000243445 | AC106820.1 |  |  | 6153 | 1.992 | 0.5311 | Yes |
| 287 | ENSG00000242970 | AC068522.1 |  |  | 6156 | 1.992 | 0.5327 | Yes |
| 288 | ENSG00000236489 | AC133473.1 |  |  | 6165 | 1.992 | 0.5342 | Yes |
| 289 | ENSG00000270723 | RPL23AP92 |  |  | 6166 | 1.992 | 0.5359 | Yes |
| 290 | ENSG00000243568 | AC020779.1 |  |  | 6213 | 1.992 | 0.5368 | Yes |
| 291 | ENSG00000243455 | RPS18P13 |  |  | 6220 | 1.992 | 0.5383 | Yes |
| 292 | ENSG00000230807 | AC099535.1 |  |  | 6222 | 1.992 | 0.5400 | Yes |
| 293 | ENSG00000213970 | AC006122.1 |  |  | 6287 | 1.977 | 0.5405 | Yes |
| 294 | ENSG00000221983 | UBA52 |  |  | 6332 | 1.966 | 0.5414 | Yes |
| 295 | ENSG00000228929 | RPS13P2 |  |  | 6342 | 1.964 | 0.5428 | Yes |
| 296 | ENSG00000234093 | RPS15AP11 |  |  | 6366 | 1.956 | 0.5441 | Yes |
| 297 | ENSG00000240914 | RPL15P2 |  |  | 6399 | 1.947 | 0.5451 | Yes |
| 298 | ENSG00000168028 | RPSA |  |  | 6459 | 1.932 | 0.5457 | Yes |
| 299 | ENSG00000159111 | MRPL10 |  |  | 6500 | 1.923 | 0.5466 | Yes |
| 300 | ENSG00000181991 | MRPS11 |  |  | 6563 | 1.909 | 0.5471 | Yes |
| 301 | ENSG00000243829 | AC011495.1 |  |  | 6596 | 1.903 | 0.5481 | Yes |
| 302 | ENSG00000197345 | MRPL21 |  |  | 6605 | 1.901 | 0.5496 | Yes |
| 303 | ENSG00000108826 | MRPL27 |  |  | 6624 | 1.895 | 0.5508 | Yes |
| 304 | ENSG00000164587 | RPS14 |  |  | 6652 | 1.889 | 0.5519 | Yes |
| 305 | ENSG00000177600 | RPLP2 |  |  | 6687 | 1.881 | 0.5529 | Yes |
| 306 | ENSG00000185834 | RPL12P4 |  |  | 6726 | 1.870 | 0.5538 | Yes |
| 307 | ENSG00000235145 | RPSAP16 |  |  | 6807 | 1.858 | 0.5540 | Yes |
| 308 | ENSG00000270733 | AL355877.2 |  |  | 6811 | 1.858 | 0.5555 | Yes |
| 309 | ENSG00000220237 | RPS24P12 |  |  | 6830 | 1.858 | 0.5567 | Yes |
| 310 | ENSG00000143314 | MRPL24 |  |  | 6842 | 1.856 | 0.5580 | Yes |
| 311 | ENSG00000101278 | RPS10P5 |  |  | 6845 | 1.855 | 0.5596 | Yes |
| 312 | ENSG00000180992 | MRPL14 |  |  | 6848 | 1.854 | 0.5611 | Yes |
| 313 | ENSG00000232713 | AC010733.1 |  |  | 6869 | 1.847 | 0.5623 | Yes |
| 314 | ENSG00000233924 | RPSAP13 |  |  | 6882 | 1.845 | 0.5636 | Yes |
| 315 | ENSG00000242477 | AC091429.1 |  |  | 6911 | 1.837 | 0.5646 | Yes |
| 316 | ENSG00000219451 | RPL23P8 |  |  | 6925 | 1.834 | 0.5659 | Yes |
| 317 | ENSG00000100316 | RPL3 |  |  | 6930 | 1.833 | 0.5674 | Yes |
| 318 | ENSG00000243071 | AC107032.1 |  |  | 6981 | 1.820 | 0.5680 | Yes |
| 319 | ENSG00000108107 | RPL28 |  |  | 6982 | 1.820 | 0.5696 | Yes |
| 320 | ENSG00000212994 | RPS26P6 |  |  | 6995 | 1.817 | 0.5709 | Yes |
| 321 | ENSG00000243498 | UBA52P5 |  |  | 7031 | 1.810 | 0.5718 | Yes |
| 322 | ENSG00000237214 | AL080243.2 |  |  | 7049 | 1.806 | 0.5730 | Yes |
| 323 | ENSG00000244363 | RPL7P23 |  |  | 7059 | 1.803 | 0.5743 | Yes |
| 324 | ENSG00000225071 | AC004552.1 |  |  | 7150 | 1.784 | 0.5742 | Yes |
| 325 | ENSG00000196183 | RPS2P4 |  |  | 7200 | 1.774 | 0.5748 | Yes |
| 326 | ENSG00000240509 | RPL34P18 |  |  | 7216 | 1.771 | 0.5761 | Yes |
| 327 | novel.612 | - |  |  | 7220 | 1.770 | 0.5775 | Yes |
| 328 | ENSG00000230799 | AC007279.1 |  |  | 7222 | 1.770 | 0.5790 | Yes |
| 329 | ENSG00000247363 | AC090061.1 |  |  | 7275 | 1.759 | 0.5795 | Yes |
| 330 | ENSG00000228477 | AL663070.1 |  |  | 7306 | 1.753 | 0.5804 | Yes |
| 331 | ENSG00000236533 | AC009413.1 |  |  | 7310 | 1.753 | 0.5819 | Yes |
| 332 | ENSG00000218426 | AL590867.2 |  |  | 7477 | 1.720 | 0.5804 | Yes |
| 333 | ENSG00000231500 | RPS18 |  |  | 7505 | 1.714 | 0.5813 | Yes |
| 334 | ENSG00000149273 | RPS3 |  |  | 7575 | 1.700 | 0.5815 | Yes |
| 335 | ENSG00000230629 | RPS23P8 |  |  | 7611 | 1.694 | 0.5823 | Yes |
| 336 | ENSG00000244398 | AC116533.1 |  |  | 7697 | 1.678 | 0.5822 | Yes |
| 337 | ENSG00000146677 | AC004453.1 |  |  | 7698 | 1.678 | 0.5836 | Yes |
| 338 | ENSG00000225093 | RPL3P7 |  |  | 7711 | 1.675 | 0.5848 | Yes |
| 339 | ENSG00000142937 | RPS8 |  |  | 7737 | 1.669 | 0.5858 | Yes |
| 340 | ENSG00000213621 | RPSAP54 |  |  | 7758 | 1.665 | 0.5868 | Yes |
| 341 | ENSG00000148303 | RPL7A |  |  | 7776 | 1.661 | 0.5879 | Yes |
| 342 | ENSG00000240036 | AC104563.1 |  |  | 7787 | 1.660 | 0.5891 | Yes |
| 343 | ENSG00000136942 | RPL35 |  |  | 7810 | 1.656 | 0.5901 | Yes |
| 344 | ENSG00000228149 | RPL3P1 |  |  | 7858 | 1.648 | 0.5907 | Yes |
| 345 | ENSG00000236439 | AC099336.2 |  |  | 7910 | 1.640 | 0.5911 | Yes |
| 346 | ENSG00000063177 | RPL18 |  |  | 7924 | 1.637 | 0.5923 | Yes |
| 347 | ENSG00000220472 | AL139095.2 |  |  | 7953 | 1.631 | 0.5931 | Yes |
| 348 | ENSG00000197756 | RPL37A |  |  | 7975 | 1.626 | 0.5941 | Yes |
| 349 | ENSG00000125445 | MRPS7 |  |  | 7990 | 1.625 | 0.5952 | Yes |
| 350 | ENSG00000089157 | RPLP0 |  |  | 8007 | 1.622 | 0.5963 | Yes |
| 351 | ENSG00000230188 | AL158156.1 |  |  | 8087 | 1.612 | 0.5963 | Yes |
| 352 | ENSG00000236992 | RPL12P12 |  |  | 8131 | 1.604 | 0.5968 | Yes |
| 353 | ENSG00000142534 | RPS11 |  |  | 8140 | 1.603 | 0.5980 | Yes |
| 354 | ENSG00000213553 | RPLP0P6 |  |  | 8187 | 1.597 | 0.5986 | Yes |
| 355 | ENSG00000136149 | RPL13AP25 |  |  | 8349 | 1.570 | 0.5970 | Yes |
| 356 | ENSG00000220848 | RPS18P9 |  |  | 8515 | 1.544 | 0.5954 | Yes |
| 357 | ENSG00000171421 | MRPL36 |  |  | 8517 | 1.544 | 0.5967 | Yes |
| 358 | ENSG00000228501 | RPL15P18 |  |  | 8522 | 1.544 | 0.5979 | Yes |
| 359 | ENSG00000213303 | AC008481.1 |  |  | 8526 | 1.544 | 0.5992 | Yes |
| 360 | ENSG00000239272 | RPL21P10 |  |  | 8528 | 1.544 | 0.6004 | Yes |
| 361 | ENSG00000142676 | RPL11 |  |  | 8542 | 1.543 | 0.6015 | Yes |
| 362 | ENSG00000266472 | MRPS21 |  |  | 8589 | 1.536 | 0.6020 | Yes |
| 363 | ENSG00000232346 | Z74021.1 |  |  | 8599 | 1.535 | 0.6031 | Yes |
| 364 | ENSG00000218175 | AC016739.1 |  |  | 8619 | 1.532 | 0.6040 | Yes |
| 365 | ENSG00000178429 | RPS3AP5 |  |  | 8690 | 1.522 | 0.6041 | Yes |
| 366 | ENSG00000239789 | MRPS17 |  |  | 8712 | 1.519 | 0.6050 | Yes |
| 367 | ENSG00000114686 | MRPL3 |  |  | 8714 | 1.518 | 0.6062 | Yes |
| 368 | ENSG00000149806 | FAU |  |  | 8719 | 1.518 | 0.6074 | Yes |
| 369 | ENSG00000198034 | RPS4X |  |  | 8797 | 1.507 | 0.6073 | Yes |
| 370 | ENSG00000147403 | RPL10 |  |  | 8925 | 1.492 | 0.6063 | Yes |
| 371 | ENSG00000183911 | RPL21P132 |  |  | 8932 | 1.491 | 0.6075 | Yes |
| 372 | ENSG00000108298 | RPL19 |  |  | 9003 | 1.480 | 0.6075 | Yes |
| 373 | ENSG00000197958 | RPL12 |  |  | 9112 | 1.465 | 0.6068 | Yes |
| 374 | ENSG00000232573 | RPL3P4 |  |  | 9131 | 1.462 | 0.6077 | Yes |
| 375 | ENSG00000241286 | RPL29P25 |  |  | 9153 | 1.460 | 0.6086 | Yes |
| 376 | ENSG00000215444 | AL138807.1 |  |  | 9155 | 1.460 | 0.6098 | Yes |
| 377 | ENSG00000240370 | RPL13P5 |  |  | 9219 | 1.450 | 0.6099 | Yes |
| 378 | ENSG00000217004 | Z97832.1 |  |  | 9296 | 1.442 | 0.6097 | Yes |
| 379 | ENSG00000232750 | AL139132.1 |  |  | 9303 | 1.442 | 0.6108 | Yes |
| 380 | ENSG00000112306 | RPS12 |  |  | 9381 | 1.433 | 0.6107 | Yes |
| 381 | ENSG00000182180 | MRPS16 |  |  | 9423 | 1.428 | 0.6111 | Yes |
| 382 | ENSG00000232075 | MRPL35P2 |  |  | 9456 | 1.422 | 0.6118 | Yes |
| 383 | ENSG00000229638 | RPL4P4 |  |  | 9469 | 1.420 | 0.6127 | Yes |
| 384 | ENSG00000162244 | RPL29 |  |  | 9524 | 1.414 | 0.6130 | Yes |
| 385 | ENSG00000235297 | FAUP1 |  |  | 9548 | 1.411 | 0.6137 | Yes |
| 386 | ENSG00000198755 | RPL10A |  |  | 9621 | 1.402 | 0.6136 | Yes |
| 387 | ENSG00000223416 | RPS26P15 |  |  | 9716 | 1.393 | 0.6131 | Yes |
| 388 | ENSG00000131469 | RPL27 |  |  | 9764 | 1.387 | 0.6135 | Yes |
| 389 | ENSG00000234750 | AC012618.2 |  |  | 9803 | 1.382 | 0.6140 | Yes |
| 390 | ENSG00000241362 | RPL36AP43 |  |  | 9804 | 1.382 | 0.6151 | Yes |
| 391 | ENSG00000225224 | RPS27AP12 |  |  | 9851 | 1.377 | 0.6155 | Yes |
| 392 | ENSG00000226107 | AC004383.1 |  |  | 9873 | 1.374 | 0.6162 | Yes |
| 393 | ENSG00000241582 | RPL23AP8 |  |  | 9917 | 1.370 | 0.6166 | Yes |
| 394 | ENSG00000276763 | AC069499.2 |  |  | 9955 | 1.365 | 0.6171 | Yes |
| 395 | novel.382 | - |  |  | 9963 | 1.364 | 0.6181 | Yes |
| 396 | ENSG00000170889 | RPS9 |  |  | 9991 | 1.360 | 0.6188 | Yes |
| 397 | ENSG00000240356 | RPL23AP7 |  |  | 10006 | 1.358 | 0.6197 | Yes |
| 398 | ENSG00000188846 | RPL14 |  |  | 10022 | 1.356 | 0.6205 | Yes |
| 399 | ENSG00000145592 | RPL37 |  |  | 10053 | 1.352 | 0.6211 | Yes |
| 400 | ENSG00000213872 | AC092798.1 |  |  | 10203 | 1.334 | 0.6196 | Yes |
| 401 | ENSG00000229994 | RPL5P4 |  |  | 10217 | 1.333 | 0.6205 | Yes |
| 402 | ENSG00000224094 | RPS24P8 |  |  | 10253 | 1.329 | 0.6210 | Yes |
| 403 | ENSG00000144713 | RPL32 |  |  | 10364 | 1.319 | 0.6202 | Yes |
| 404 | ENSG00000220583 | RPL35P2 |  |  | 10378 | 1.318 | 0.6211 | Yes |
| 405 | ENSG00000227525 | RPL7P6 |  |  | 10422 | 1.318 | 0.6214 | Yes |
| 406 | ENSG00000236887 | AL357055.2 |  |  | 10446 | 1.318 | 0.6221 | Yes |
| 407 | ENSG00000239397 | AC084878.1 |  |  | 10457 | 1.318 | 0.6230 | Yes |
| 408 | ENSG00000229759 | MRPS18AP1 |  |  | 10471 | 1.318 | 0.6239 | Yes |
| 409 | ENSG00000224858 | RPL29P11 |  |  | 10507 | 1.318 | 0.6244 | Yes |
| 410 | ENSG00000172809 | RPL38 |  |  | 10514 | 1.316 | 0.6254 | Yes |
| 411 | ENSG00000096080 | MRPS18A |  |  | 10548 | 1.312 | 0.6259 | Yes |
| 412 | ENSG00000212802 | RPL15P3 |  |  | 10579 | 1.308 | 0.6264 | Yes |
| 413 | ENSG00000243697 | AC009108.1 |  |  | 10690 | 1.295 | 0.6256 | Yes |
| 414 | ENSG00000219470 | AL355802.1 |  |  | 10710 | 1.294 | 0.6263 | Yes |
| 415 | ENSG00000213753 | CENPBD1P1 |  |  | 10743 | 1.290 | 0.6269 | Yes |
| 416 | ENSG00000226084 | AC113935.1 |  |  | 10757 | 1.288 | 0.6277 | Yes |
| 417 | ENSG00000237991 | RPL35P1 |  |  | 10763 | 1.288 | 0.6287 | Yes |
| 418 | ENSG00000224993 | RPL29P12 |  |  | 10765 | 1.287 | 0.6297 | Yes |
| 419 | ENSG00000071082 | RPL31 |  |  | 10803 | 1.283 | 0.6302 | Yes |
| 420 | novel.384 | - |  |  | 10806 | 1.283 | 0.6312 | Yes |
| 421 | ENSG00000242737 | AC012170.1 |  |  | 10848 | 1.279 | 0.6316 | Yes |
| 422 | ENSG00000134419 | RPS15A |  |  | 10871 | 1.277 | 0.6322 | Yes |
| 423 | ENSG00000242485 | MRPL20 |  |  | 10876 | 1.277 | 0.6332 | Yes |
| 424 | ENSG00000227470 | AC073415.1 |  |  | 10996 | 1.265 | 0.6322 | Yes |
| 425 | ENSG00000178464 | RPL10P16 |  |  | 11042 | 1.260 | 0.6324 | Yes |
| 426 | ENSG00000112110 | MRPL18 |  |  | 11077 | 1.258 | 0.6329 | Yes |
| 427 | ENSG00000163584 | RPL22L1 |  |  | 11114 | 1.255 | 0.6333 | Yes |
| 428 | ENSG00000115364 | MRPL19 |  |  | 11183 | 1.249 | 0.6332 | Yes |
| 429 | ENSG00000213385 | AC105052.2 |  |  | 11205 | 1.247 | 0.6338 | Yes |
| 430 | ENSG00000137547 | MRPL15 |  |  | 11239 | 1.244 | 0.6343 | Yes |
| 431 | ENSG00000218227 | AC136632.1 |  |  | 11323 | 1.235 | 0.6339 | Yes |
| 432 | ENSG00000213178 | RPL22P1 |  |  | 11327 | 1.235 | 0.6348 | Yes |
| 433 | ENSG00000213752 | AL008718.1 |  |  | 11409 | 1.230 | 0.6344 | Yes |
| 434 | ENSG00000244538 | AC098583.1 |  |  | 11437 | 1.230 | 0.6350 | Yes |
| 435 | ENSG00000243742 | RPLP0P2 |  |  | 11529 | 1.225 | 0.6344 | Yes |
| 436 | ENSG00000105372 | RPS19 |  |  | 11562 | 1.222 | 0.6349 | Yes |
| 437 | ENSG00000142541 | RPL13A |  |  | 11594 | 1.219 | 0.6353 | Yes |
| 438 | ENSG00000174444 | RPL4 |  |  | 11623 | 1.216 | 0.6359 | Yes |
| 439 | ENSG00000172172 | MRPL13 |  |  | 11632 | 1.216 | 0.6367 | Yes |
| 440 | ENSG00000008988 | RPS20 |  |  | 11649 | 1.214 | 0.6375 | Yes |
| 441 | ENSG00000227615 | AP001324.1 |  |  | 11748 | 1.205 | 0.6368 | Yes |
| 442 | ENSG00000186468 | RPS23 |  |  | 11772 | 1.204 | 0.6374 | Yes |
| 443 | ENSG00000082515 | MRPL22 |  |  | 11779 | 1.203 | 0.6383 | Yes |
| 444 | ENSG00000177954 | RPS27 |  |  | 11814 | 1.199 | 0.6387 | Yes |
| 445 | ENSG00000242071 | RPL7AP6 |  |  | 11868 | 1.194 | 0.6387 | Yes |
| 446 | ENSG00000165502 | RPL36AL |  |  | 11891 | 1.191 | 0.6393 | Yes |
| 447 | ENSG00000226706 | AL161452.1 |  |  | 11898 | 1.191 | 0.6402 | Yes |
| 448 | ENSG00000263266 | RPS7P1 |  |  | 11912 | 1.189 | 0.6410 | Yes |
| 449 | ENSG00000243199 | AC115223.1 |  |  | 11942 | 1.186 | 0.6415 | Yes |
| 450 | ENSG00000179818 | PCBP1-AS1 |  |  | 11946 | 1.186 | 0.6424 | Yes |
| 451 | ENSG00000214389 | RPS3AP26 |  |  | 12063 | 1.177 | 0.6413 | Yes |
| 452 | ENSG00000120333 | MRPS14 |  |  | 12122 | 1.172 | 0.6413 | Yes |
| 453 | ENSG00000234797 | RPS3AP6 |  |  | 12130 | 1.171 | 0.6422 | Yes |
| 454 | ENSG00000213862 | AC044787.1 |  |  | 12134 | 1.170 | 0.6431 | Yes |
| 455 | ENSG00000130312 | MRPL34 |  |  | 12140 | 1.170 | 0.6440 | Yes |
| 456 | ENSG00000213272 | RPL7AP9 |  |  | 12290 | 1.157 | 0.6423 | Yes |
| 457 | ENSG00000116898 | MRPS15 |  |  | 12301 | 1.156 | 0.6431 | Yes |
| 458 | ENSG00000238003 | RPL10P4 |  |  | 12319 | 1.155 | 0.6438 | Yes |
| 459 | ENSG00000241941 | RPL32P26 |  |  | 12327 | 1.155 | 0.6446 | Yes |
| 460 | ENSG00000137154 | RPS6 |  |  | 12419 | 1.148 | 0.6440 | Yes |
| 461 | ENSG00000213860 | RPL21P75 |  |  | 12448 | 1.146 | 0.6444 | Yes |
| 462 | ENSG00000169288 | MRPL1 |  |  | 12460 | 1.144 | 0.6452 | Yes |
| 463 | ENSG00000220842 | RPL21P16 |  |  | 12486 | 1.142 | 0.6457 | Yes |
| 464 | ENSG00000234118 | RPL13AP6 |  |  | 12508 | 1.140 | 0.6463 | Yes |
| 465 | ENSG00000125691 | RPL23 |  |  | 12518 | 1.139 | 0.6471 | Yes |
| 466 | ENSG00000143436 | MRPL9 |  |  | 12650 | 1.128 | 0.6457 | Yes |
| 467 | ENSG00000265681 | RPL17 |  |  | 12654 | 1.128 | 0.6466 | Yes |
| 468 | ENSG00000110700 | RPS13 |  |  | 12707 | 1.124 | 0.6466 | Yes |
| 469 | ENSG00000233597 | AC133435.1 |  |  | 12799 | 1.117 | 0.6460 | Yes |
| 470 | ENSG00000236058 | RPL17P36 |  |  | 12809 | 1.117 | 0.6467 | Yes |
| 471 | ENSG00000144029 | MRPS5 |  |  | 12830 | 1.115 | 0.6473 | Yes |
| 472 | ENSG00000231084 | RPL22P24 |  |  | 12868 | 1.111 | 0.6476 | Yes |
| 473 | ENSG00000224629 | AC004975.1 |  |  | 12875 | 1.110 | 0.6484 | Yes |
| 474 | ENSG00000181524 | RPL24P4 |  |  | 12895 | 1.109 | 0.6490 | Yes |
| 475 | ENSG00000227304 | AC067942.1 |  |  | 12946 | 1.104 | 0.6490 | Yes |
| 476 | ENSG00000236552 | RPL13AP5 |  |  | 12951 | 1.104 | 0.6499 | Yes |
| 477 | ENSG00000163319 | MRPS18C |  |  | 12963 | 1.104 | 0.6506 | Yes |
| 478 | ENSG00000138326 | RPS24 |  |  | 13026 | 1.100 | 0.6504 | Yes |
| 479 | ENSG00000219027 | RPS3AP2 |  |  | 13058 | 1.098 | 0.6508 | Yes |
| 480 | ENSG00000161970 | RPL26 |  |  | 13086 | 1.096 | 0.6513 | Yes |
| 481 | ENSG00000176054 | RPL23P2 |  |  | 13313 | 1.078 | 0.6482 | Yes |
| 482 | ENSG00000225573 | RPL35P5 |  |  | 13323 | 1.078 | 0.6489 | Yes |
| 483 | ENSG00000235552 | RPL6P27 |  |  | 13355 | 1.076 | 0.6493 | Yes |
| 484 | ENSG00000230207 | RPL4P5 |  |  | 13481 | 1.066 | 0.6480 | Yes |
| 485 | ENSG00000241962 | AC079447.1 |  |  | 13535 | 1.061 | 0.6479 | Yes |
| 486 | ENSG00000230202 | AL450405.1 |  |  | 13582 | 1.058 | 0.6480 | Yes |
| 487 | ENSG00000174748 | RPL15 |  |  | 13686 | 1.051 | 0.6471 | Yes |
| 488 | ENSG00000266891 | AP000902.1 |  |  | 13701 | 1.049 | 0.6477 | Yes |
| 489 | ENSG00000237506 | RPSAP15 |  |  | 13726 | 1.048 | 0.6481 | Yes |
| 490 | ENSG00000213326 | RPS7P11 |  |  | 13727 | 1.048 | 0.6490 | Yes |
| 491 | ENSG00000235174 | RPL39P3 |  |  | 13829 | 1.039 | 0.6481 | Yes |
| 492 | ENSG00000232134 | RPS15AP12 |  |  | 13961 | 1.029 | 0.6467 | Yes |
| 493 | ENSG00000269737 | AL691432.1 |  |  | 13976 | 1.028 | 0.6473 | Yes |
| 494 | ENSG00000224631 | RPS27AP16 |  |  | 14074 | 1.020 | 0.6464 | Yes |
| 495 | ENSG00000223508 | RPL23AP53 |  |  | 14232 | 1.008 | 0.6445 | Yes |
| 496 | ENSG00000234498 | RPL13AP20 |  |  | 14391 | 0.996 | 0.6425 | Yes |
| 497 | ENSG00000270285 | AC124916.2 |  |  | 14410 | 0.996 | 0.6431 | Yes |
| 498 | ENSG00000270802 | AC005776.1 |  |  | 14433 | 0.996 | 0.6435 | Yes |
| 499 | ENSG00000247151 | CSTF3-AS1 |  |  | 14442 | 0.996 | 0.6442 | Yes |
| 500 | ENSG00000213063 | RPL29P7 |  |  | 14444 | 0.996 | 0.6450 | Yes |
| 501 | ENSG00000241187 | AC008379.1 |  |  | 14469 | 0.996 | 0.6454 | Yes |
| 502 | ENSG00000258439 | AC007956.1 |  |  | 14506 | 0.996 | 0.6456 | Yes |
| 503 | ENSG00000239617 | AC073610.1 |  |  | 14510 | 0.996 | 0.6464 | Yes |
| 504 | ENSG00000235077 | AC073842.1 |  |  | 14516 | 0.996 | 0.6471 | Yes |
| 505 | ENSG00000233254 | RPL21P134 |  |  | 14540 | 0.996 | 0.6476 | Yes |
| 506 | ENSG00000132313 | MRPL35 |  |  | 14613 | 0.995 | 0.6471 | Yes |
| 507 | ENSG00000230383 | AC009245.1 |  |  | 14616 | 0.995 | 0.6479 | Yes |
| 508 | ENSG00000228019 | AC006480.1 |  |  | 14673 | 0.991 | 0.6478 | Yes |
| 509 | ENSG00000182774 | RPS17 |  |  | 14810 | 0.981 | 0.6462 | Yes |
| 510 | ENSG00000235776 | AC000089.1 |  |  | 14825 | 0.981 | 0.6468 | Yes |
| 511 | ENSG00000048544 | MRPS10 |  |  | 14918 | 0.975 | 0.6459 | Yes |
| 512 | ENSG00000122026 | RPL21 |  |  | 14978 | 0.971 | 0.6457 | Yes |
| 513 | ENSG00000275160 | AL354718.1 |  |  | 14987 | 0.971 | 0.6464 | Yes |
| 514 | ENSG00000227081 | AC005912.1 |  |  | 15013 | 0.969 | 0.6468 | Yes |
| 515 | ENSG00000233084 | RPL23AP25 |  |  | 15035 | 0.968 | 0.6472 | Yes |
| 516 | ENSG00000122406 | RPL5 |  |  | 15041 | 0.968 | 0.6479 | Yes |
| 517 | ENSG00000171863 | RPS7 |  |  | 15081 | 0.965 | 0.6480 | Yes |
| 518 | ENSG00000238103 | RPL9P7 |  |  | 15087 | 0.965 | 0.6488 | Yes |
| 519 | ENSG00000236801 | RPL24P8 |  |  | 15100 | 0.964 | 0.6494 | Yes |
| 520 | ENSG00000214485 | RPL7P1 |  |  | 15105 | 0.964 | 0.6501 | Yes |
| 521 | ENSG00000235990 | RPL23AP20 |  |  | 15159 | 0.961 | 0.6500 | Yes |
| 522 | ENSG00000231804 | AL353748.1 |  |  | 15168 | 0.961 | 0.6506 | Yes |
| 523 | ENSG00000116251 | RPL22 |  |  | 15186 | 0.959 | 0.6511 | Yes |
| 524 | ENSG00000236047 | AC073410.1 |  |  | 15262 | 0.954 | 0.6506 | Yes |
| 525 | ENSG00000241494 | AL355032.1 |  |  | 15273 | 0.954 | 0.6512 | Yes |
| 526 | ENSG00000214535 | RPS15AP1 |  |  | 15285 | 0.953 | 0.6518 | Yes |
| 527 | ENSG00000114391 | RPL24 |  |  | 15304 | 0.952 | 0.6523 | Yes |
| 528 | ENSG00000185414 | MRPL30 |  |  | 15384 | 0.947 | 0.6517 | Yes |
| 529 | ENSG00000231181 | AL954705.1 |  |  | 15405 | 0.946 | 0.6521 | Yes |
| 530 | ENSG00000240392 | RPL9P3 |  |  | 15691 | 0.929 | 0.6479 | Yes |
| 531 | ENSG00000213695 | RPS7P14 |  |  | 15697 | 0.929 | 0.6486 | Yes |
| 532 | ENSG00000249721 | AC112206.3 |  |  | 15708 | 0.929 | 0.6492 | Yes |
| 533 | ENSG00000180662 | RPL21P8 |  |  | 15736 | 0.929 | 0.6495 | Yes |
| 534 | ENSG00000232333 | RPS27AP2 |  |  | 15740 | 0.929 | 0.6502 | Yes |
| 535 | ENSG00000242325 | RPS12P31 |  |  | 15774 | 0.929 | 0.6504 | Yes |
| 536 | ENSG00000223718 | AC093107.1 |  |  | 15803 | 0.929 | 0.6507 | Yes |
| 537 | ENSG00000241853 | AC097484.1 |  |  | 15806 | 0.929 | 0.6514 | Yes |
| 538 | ENSG00000225419 | RPL21P3 |  |  | 15807 | 0.929 | 0.6522 | Yes |
| 539 | ENSG00000143947 | RPS27A |  |  | 15847 | 0.929 | 0.6523 | Yes |
| 540 | ENSG00000239365 | RPS26P49 |  |  | 15878 | 0.927 | 0.6525 | Yes |
| 541 | ENSG00000237550 | RPL9P9 |  |  | 15990 | 0.920 | 0.6513 | No |
| 542 | ENSG00000198242 | RPL23A |  |  | 16099 | 0.913 | 0.6502 | No |
| 543 | ENSG00000185641 | AC034236.1 |  |  | 16127 | 0.911 | 0.6505 | No |
| 544 | ENSG00000232450 | AL133517.1 |  |  | 16248 | 0.902 | 0.6491 | No |
| 545 | ENSG00000231416 | AL358472.1 |  |  | 16299 | 0.900 | 0.6490 | No |
| 546 | ENSG00000219355 | RPL31P52 |  |  | 16373 | 0.896 | 0.6485 | No |
| 547 | ENSG00000106591 | MRPL32 |  |  | 16417 | 0.893 | 0.6485 | No |
| 548 | ENSG00000109475 | RPL34 |  |  | 16562 | 0.884 | 0.6467 | No |
| 549 | ENSG00000198918 | RPL39 |  |  | 16613 | 0.881 | 0.6465 | No |
| 550 | ENSG00000182899 | RPL35A |  |  | 16643 | 0.880 | 0.6467 | No |
| 551 | ENSG00000234851 | RPL23AP42 |  |  | 16670 | 0.878 | 0.6470 | No |
| 552 | ENSG00000244060 | RPS2P41 |  |  | 16695 | 0.877 | 0.6473 | No |
| 553 | ENSG00000243927 | MRPS6 |  |  | 16719 | 0.875 | 0.6476 | No |
| 554 | ENSG00000089009 | RPL6 |  |  | 16873 | 0.865 | 0.6457 | No |
| 555 | ENSG00000135972 | MRPS9 |  |  | 16933 | 0.861 | 0.6454 | No |
| 556 | ENSG00000220793 | RPL21P119 |  |  | 16936 | 0.861 | 0.6460 | No |
| 557 | ENSG00000242748 | RPL23AP81 |  |  | 17012 | 0.856 | 0.6454 | No |
| 558 | ENSG00000145425 | RPS3A |  |  | 17133 | 0.849 | 0.6440 | No |
| 559 | ENSG00000234371 | RPSAP31 |  |  | 17298 | 0.839 | 0.6418 | No |
| 560 | ENSG00000243517 | AC024940.3 |  |  | 17304 | 0.839 | 0.6425 | No |
| 561 | ENSG00000163682 | RPL9 |  |  | 17339 | 0.837 | 0.6426 | No |
| 562 | ENSG00000147604 | RPL7 |  |  | 17617 | 0.820 | 0.6384 | No |
| 563 | ENSG00000240567 | LINC02067 |  |  | 17738 | 0.813 | 0.6369 | No |
| 564 | ENSG00000213542 | AC007000.1 |  |  | 17758 | 0.812 | 0.6373 | No |
| 565 | ENSG00000237039 | AC018738.1 |  |  | 17915 | 0.804 | 0.6352 | No |
| 566 | ENSG00000241343 | RPL36A |  |  | 18026 | 0.798 | 0.6339 | No |
| 567 | ENSG00000232888 | RPS11P5 |  |  | 18167 | 0.789 | 0.6321 | No |
| 568 | ENSG00000137876 | RSL24D1 |  |  | 18275 | 0.783 | 0.6309 | No |
| 569 | ENSG00000244176 | AP003733.1 |  |  | 18341 | 0.779 | 0.6304 | No |
| 570 | ENSG00000267119 | RPL10P15 |  |  | 18497 | 0.771 | 0.6283 | No |
| 571 | ENSG00000185088 | RPS27L |  |  | 18513 | 0.771 | 0.6287 | No |
| 572 | ENSG00000224221 | RPS6P8 |  |  | 18993 | 0.743 | 0.6209 | No |
| 573 | ENSG00000224452 | RSL24D1P6 |  |  | 19272 | 0.727 | 0.6166 | No |
| 574 | ENSG00000240634 | AC145285.1 |  |  | 19490 | 0.716 | 0.6134 | No |
| 575 | ENSG00000235065 | RPL24P2 |  |  | 19503 | 0.715 | 0.6137 | No |
| 576 | ENSG00000234618 | RPSAP9 |  |  | 19504 | 0.715 | 0.6143 | No |
| 577 | ENSG00000137970 | RPL7P9 |  |  | 19519 | 0.714 | 0.6147 | No |
| 578 | ENSG00000242085 | RPS20P33 |  |  | 19734 | 0.703 | 0.6115 | No |
| 579 | ENSG00000234329 | AL604028.2 |  |  | 19759 | 0.702 | 0.6117 | No |
| 580 | ENSG00000124614 | RPS10 |  |  | 19766 | 0.702 | 0.6122 | No |
| 581 | ENSG00000234009 | RPL5P34 |  |  | 19792 | 0.700 | 0.6123 | No |
| 582 | ENSG00000234450 | AC005534.2 |  |  | 19803 | 0.699 | 0.6127 | No |
| 583 | ENSG00000237169 | RPL12P27 |  |  | 19850 | 0.697 | 0.6125 | No |
| 584 | ENSG00000248794 | AC026436.1 |  |  | 20238 | 0.675 | 0.6062 | No |
| 585 | ENSG00000239763 | AC009120.1 |  |  | 20276 | 0.672 | 0.6061 | No |
| 586 | ENSG00000215464 | AP000354.1 |  |  | 20429 | 0.664 | 0.6040 | No |
| 587 | ENSG00000232163 | RPLP1P13 |  |  | 20521 | 0.659 | 0.6030 | No |
| 588 | ENSG00000242615 | AC022415.1 |  |  | 20629 | 0.654 | 0.6016 | No |
| 589 | ENSG00000264577 | AC010761.1 |  |  | 20679 | 0.651 | 0.6013 | No |
| 590 | ENSG00000227775 | AL031282.1 |  |  | 20693 | 0.650 | 0.6016 | No |
| 591 | ENSG00000224646 | AC007387.1 |  |  | 20705 | 0.650 | 0.6020 | No |
| 592 | ENSG00000232342 | AC022540.1 |  |  | 20731 | 0.648 | 0.6021 | No |
| 593 | ENSG00000239917 | RPS10P16 |  |  | 20978 | 0.636 | 0.5983 | No |
| 594 | ENSG00000237433 | RPSAP11 |  |  | 20998 | 0.636 | 0.5985 | No |
| 595 | ENSG00000245970 | AP003352.1 |  |  | 21158 | 0.628 | 0.5962 | No |
| 596 | ENSG00000225200 | AC246787.1 |  |  | 21352 | 0.617 | 0.5933 | No |
| 597 | ENSG00000239510 | RPL9P5 |  |  | 21381 | 0.615 | 0.5933 | No |
| 598 | ENSG00000216639 | AL133406.1 |  |  | 21407 | 0.615 | 0.5934 | No |
| 599 | ENSG00000213755 | AC093270.1 |  |  | 21416 | 0.615 | 0.5938 | No |
| 600 | ENSG00000243024 | RPS11P6 |  |  | 21627 | 0.600 | 0.5906 | No |
| 601 | ENSG00000241157 | AC104763.1 |  |  | 21945 | 0.580 | 0.5855 | No |
| 602 | ENSG00000241907 | RPS20P4 |  |  | 22068 | 0.573 | 0.5838 | No |
| 603 | ENSG00000223861 | AL365436.1 |  |  | 22084 | 0.572 | 0.5840 | No |
| 604 | ENSG00000228463 | AP006222.1 |  |  | 22303 | 0.557 | 0.5806 | No |
| 605 | ENSG00000240522 | RPL7AP10 |  |  | 22332 | 0.555 | 0.5806 | No |
| 606 | ENSG00000240399 | AC004801.2 |  |  | 22430 | 0.549 | 0.5794 | No |
| 607 | ENSG00000235330 | RPL12P25 |  |  | 22624 | 0.535 | 0.5764 | No |
| 608 | ENSG00000236830 | CBR3-AS1 |  |  | 22680 | 0.531 | 0.5759 | No |
| 609 | ENSG00000224331 | AC019181.1 |  |  | 22736 | 0.526 | 0.5754 | No |
| 610 | ENSG00000231307 | RPS3P2 |  |  | 22904 | 0.514 | 0.5729 | No |
| 611 | ENSG00000240051 | RPL23AP10 |  |  | 22906 | 0.513 | 0.5733 | No |
| 612 | ENSG00000214988 | RPL7AP26 |  |  | 23046 | 0.503 | 0.5712 | No |
| 613 | ENSG00000118181 | RPS25 |  |  | 23079 | 0.500 | 0.5711 | No |
| 614 | ENSG00000228820 | RPSAP1 |  |  | 23117 | 0.498 | 0.5709 | No |
| 615 | ENSG00000241913 | RPS29P21 |  |  | 23137 | 0.498 | 0.5709 | No |
| 616 | ENSG00000229870 | RPL21P89 |  |  | 23145 | 0.498 | 0.5712 | No |
| 617 | ENSG00000219133 | AL592114.1 |  |  | 23151 | 0.498 | 0.5716 | No |
| 618 | ENSG00000233984 | RPSAP14 |  |  | 23154 | 0.498 | 0.5719 | No |
| 619 | ENSG00000216867 | RPL22P12 |  |  | 23167 | 0.498 | 0.5721 | No |
| 620 | ENSG00000267693 | AC011477.5 |  |  | 23169 | 0.498 | 0.5725 | No |
| 621 | ENSG00000239291 | AC002558.1 |  |  | 23293 | 0.489 | 0.5708 | No |
| 622 | ENSG00000240163 | AC087385.1 |  |  | 23317 | 0.487 | 0.5708 | No |
| 623 | ENSG00000223876 | RPS27P1 |  |  | 23436 | 0.481 | 0.5691 | No |
| 624 | ENSG00000240991 | RPL23AP67 |  |  | 23469 | 0.481 | 0.5689 | No |
| 625 | ENSG00000239912 | RPL39P36 |  |  | 23491 | 0.479 | 0.5690 | No |
| 626 | ENSG00000232344 | AC087163.2 |  |  | 23534 | 0.475 | 0.5686 | No |
| 627 | ENSG00000232938 | RPL23AP87 |  |  | 23563 | 0.473 | 0.5685 | No |
| 628 | ENSG00000237749 | AL034379.1 |  |  | 23689 | 0.464 | 0.5667 | No |
| 629 | ENSG00000230783 | AC009961.3 |  |  | 23857 | 0.450 | 0.5642 | No |
| 630 | ENSG00000244712 | AC084754.1 |  |  | 24111 | 0.429 | 0.5601 | No |
| 631 | ENSG00000231702 | AL645608.4 |  |  | 24116 | 0.429 | 0.5603 | No |
| 632 | ENSG00000223849 | AL354893.1 |  |  | 24122 | 0.428 | 0.5606 | No |
| 633 | ENSG00000239881 | RPS27P25 |  |  | 24221 | 0.419 | 0.5592 | No |
| 634 | ENSG00000223396 | RPS10P7 |  |  | 24389 | 0.408 | 0.5566 | No |
| 635 | ENSG00000213013 | RPS15AP36 |  |  | 24417 | 0.406 | 0.5565 | No |
| 636 | ENSG00000229839 | AC018462.1 |  |  | 24421 | 0.405 | 0.5568 | No |
| 637 | ENSG00000243020 | RPL7P39 |  |  | 24451 | 0.403 | 0.5566 | No |
| 638 | ENSG00000228955 | AL591623.2 |  |  | 24459 | 0.402 | 0.5568 | No |
| 639 | ENSG00000236732 | AC094019.1 |  |  | 24468 | 0.402 | 0.5570 | No |
| 640 | ENSG00000224321 | RPL12P14 |  |  | 24658 | 0.386 | 0.5540 | No |
| 641 | ENSG00000243007 | RPL12P35 |  |  | 24694 | 0.382 | 0.5537 | No |
| 642 | ENSG00000229659 | RPL26P6 |  |  | 24929 | 0.360 | 0.5499 | No |
| 643 | ENSG00000230071 | RPL4P6 |  |  | 24996 | 0.353 | 0.5490 | No |
| 644 | ENSG00000240793 | UBA52P8 |  |  | 24999 | 0.353 | 0.5493 | No |
| 645 | ENSG00000235354 | RPS29P16 |  |  | 25016 | 0.351 | 0.5493 | No |
| 646 | ENSG00000229519 | AL591866.1 |  |  | 25050 | 0.347 | 0.5490 | No |
| 647 | ENSG00000237641 | AC073476.1 |  |  | 25098 | 0.338 | 0.5485 | No |
| 648 | ENSG00000227123 | RPL12P44 |  |  | 25166 | 0.332 | 0.5476 | No |
| 649 | ENSG00000173727 | AP000769.1 |  |  | 25191 | 0.331 | 0.5474 | No |
| 650 | ENSG00000245281 | AC124242.1 |  |  | 25322 | 0.320 | 0.5454 | No |
| 651 | ENSG00000213700 | RPL17P50 |  |  | 25327 | 0.320 | 0.5456 | No |
| 652 | ENSG00000226493 | RPL26P27 |  |  | 25335 | 0.319 | 0.5457 | No |
| 653 | ENSG00000259121 | AL109628.1 |  |  | 25373 | 0.317 | 0.5453 | No |
| 654 | ENSG00000240023 | AL133163.1 |  |  | 25381 | 0.317 | 0.5455 | No |
| 655 | ENSG00000267340 | AC060780.2 |  |  | 25616 | 0.293 | 0.5416 | No |
| 656 | ENSG00000225193 | RPS12P26 |  |  | 25718 | 0.280 | 0.5401 | No |
| 657 | ENSG00000229677 | AC018644.1 |  |  | 25782 | 0.273 | 0.5392 | No |
| 658 | ENSG00000230118 | AC092569.1 |  |  | 25931 | 0.253 | 0.5368 | No |
| 659 | ENSG00000240898 | AC132942.1 |  |  | 26010 | 0.247 | 0.5356 | No |
| 660 | ENSG00000228879 | AL591848.1 |  |  | 26034 | 0.245 | 0.5354 | No |
| 661 | ENSG00000225067 | RPL23AP2 |  |  | 26038 | 0.245 | 0.5356 | No |
| 662 | ENSG00000213152 | RPL7AP60 |  |  | 26078 | 0.240 | 0.5351 | No |
| 663 | ENSG00000270679 | AC138473.1 |  |  | 26087 | 0.240 | 0.5351 | No |
| 664 | ENSG00000231359 | AC072052.1 |  |  | 26126 | 0.238 | 0.5347 | No |
| 665 | ENSG00000228874 | AC090954.1 |  |  | 26141 | 0.236 | 0.5346 | No |
| 666 | ENSG00000226457 | RPL22P3 |  |  | 26144 | 0.236 | 0.5348 | No |
| 667 | ENSG00000254974 | AP001372.4 |  |  | 26177 | 0.232 | 0.5344 | No |
| 668 | ENSG00000219702 | AL078599.1 |  |  | 26199 | 0.230 | 0.5342 | No |
| 669 | ENSG00000226318 | RPS3AP38 |  |  | 26221 | 0.226 | 0.5341 | No |
| 670 | ENSG00000236679 | RPL23AP24 |  |  | 26393 | 0.201 | 0.5312 | No |
| 671 | ENSG00000213253 | RPL12P42 |  |  | 26402 | 0.199 | 0.5312 | No |
| 672 | ENSG00000230929 | AC099513.1 |  |  | 26450 | 0.196 | 0.5306 | No |
| 673 | ENSG00000235328 | AC006946.1 |  |  | 26462 | 0.194 | 0.5305 | No |
| 674 | ENSG00000243101 | RPS3P7 |  |  | 26481 | 0.194 | 0.5304 | No |
| 675 | ENSG00000243225 | AC007686.1 |  |  | 26484 | 0.194 | 0.5305 | No |
| 676 | ENSG00000237418 | AC069257.2 |  |  | 26516 | 0.190 | 0.5301 | No |
| 677 | ENSG00000225447 | RPS15AP10 |  |  | 26532 | 0.188 | 0.5300 | No |
| 678 | ENSG00000213433 | RPLP1P6 |  |  | 26539 | 0.186 | 0.5301 | No |
| 679 | ENSG00000233309 | RPS6P12 |  |  | 26566 | 0.184 | 0.5298 | No |
| 680 | ENSG00000240463 | RPS19P3 |  |  | 26618 | 0.176 | 0.5290 | No |
| 681 | ENSG00000241890 | RPL13P4 |  |  | 26690 | 0.164 | 0.5279 | No |
| 682 | ENSG00000219755 | AL137784.1 |  |  | 26705 | 0.162 | 0.5278 | No |
| 683 | ENSG00000229853 | AL034418.1 |  |  | 26721 | 0.160 | 0.5277 | No |
| 684 | ENSG00000232818 | RPS2P32 |  |  | 26749 | 0.158 | 0.5273 | No |
| 685 | ENSG00000231940 | RPS7P3 |  |  | 26757 | 0.158 | 0.5273 | No |
| 686 | ENSG00000197149 | AC107956.1 |  |  | 26777 | 0.154 | 0.5271 | No |
| 687 | ENSG00000223945 | AC244033.1 |  |  | 26783 | 0.152 | 0.5272 | No |
| 688 | ENSG00000213598 | AL049873.1 |  |  | 26858 | 0.138 | 0.5260 | No |
| 689 | ENSG00000240003 | RPL7P24 |  |  | 26881 | 0.135 | 0.5257 | No |
| 690 | ENSG00000242307 | RPS26P52 |  |  | 26899 | 0.131 | 0.5255 | No |
| 691 | ENSG00000241984 | RPL7AP2 |  |  | 26949 | 0.121 | 0.5247 | No |
| 692 | ENSG00000220920 | AL023807.1 |  |  | 27044 | 0.102 | 0.5232 | No |
| 693 | ENSG00000243431 | RPL5P30 |  |  | 27064 | 0.097 | 0.5229 | No |
| 694 | ENSG00000228349 | RPS26P4 |  |  | 27268 | 0.000 | 0.5193 | No |
| 695 | ENSG00000228776 | AC114492.1 |  |  | 27297 | 0.000 | 0.5188 | No |
| 696 | ENSG00000220614 | AL583834.1 |  |  | 27307 | 0.000 | 0.5187 | No |
| 697 | ENSG00000224064 | AL356390.1 |  |  | 27327 | 0.000 | 0.5184 | No |
| 698 | ENSG00000256552 | AC092745.2 |  |  | 27344 | 0.000 | 0.5181 | No |
| 699 | ENSG00000278586 | AC005088.1 |  |  | 27352 | 0.000 | 0.5179 | No |
| 700 | ENSG00000236804 | RPS3AP12 |  |  | 27362 | 0.000 | 0.5178 | No |
| 701 | ENSG00000236806 | RPL7AP15 |  |  | 27363 | 0.000 | 0.5178 | No |
| 702 | ENSG00000229657 | AL391822.1 |  |  | 27369 | 0.000 | 0.5177 | No |
| 703 | ENSG00000259258 | AC013553.1 |  |  | 27377 | 0.000 | 0.5176 | No |
| 704 | ENSG00000242295 | AC087477.1 |  |  | 27385 | 0.000 | 0.5175 | No |
| 705 | ENSG00000230777 | RPS29P5 |  |  | 27388 | 0.000 | 0.5174 | No |
| 706 | ENSG00000240970 | RPL23AP64 |  |  | 27408 | 0.000 | 0.5171 | No |
| 707 | ENSG00000214067 | AL360182.1 |  |  | 27416 | 0.000 | 0.5170 | No |
| 708 | ENSG00000213950 | RPS10P2 |  |  | 27467 | 0.000 | 0.5161 | No |
| 709 | ENSG00000226855 | RPSAP17 |  |  | 27473 | 0.000 | 0.5160 | No |
| 710 | ENSG00000244722 | RPSAP29 |  |  | 27490 | 0.000 | 0.5157 | No |
| 711 | ENSG00000214612 | RPS19P1 |  |  | 27499 | 0.000 | 0.5156 | No |
| 712 | ENSG00000240436 | AC114982.1 |  |  | 27531 | 0.000 | 0.5150 | No |
| 713 | ENSG00000213315 | AL122020.1 |  |  | 27538 | 0.000 | 0.5149 | No |
| 714 | ENSG00000184111 | RPL11P4 |  |  | 27542 | 0.000 | 0.5149 | No |
| 715 | ENSG00000244378 | RPS2P45 |  |  | 27570 | 0.000 | 0.5144 | No |
| 716 | ENSG00000235045 | RPL7P8 |  |  | 27603 | 0.000 | 0.5138 | No |
| 717 | ENSG00000234319 | RPS12P2 |  |  | 27606 | 0.000 | 0.5138 | No |
| 718 | ENSG00000227383 | AL353662.1 |  |  | 27614 | 0.000 | 0.5137 | No |
| 719 | ENSG00000230175 | AL451074.4 |  |  | 27619 | 0.000 | 0.5136 | No |
| 720 | ENSG00000241749 | RPSAP52 |  |  | 27623 | 0.000 | 0.5135 | No |
| 721 | ENSG00000268105 | AC124856.1 |  |  | 27636 | 0.000 | 0.5133 | No |
| 722 | ENSG00000232946 | AL354707.3 |  |  | 27664 | 0.000 | 0.5129 | No |
| 723 | ENSG00000250140 | AC026801.1 |  |  | 27689 | 0.000 | 0.5124 | No |
| 724 | ENSG00000218757 | AL121952.1 |  |  | 27725 | 0.000 | 0.5118 | No |
| 725 | ENSG00000240995 | AC069454.1 |  |  | 27729 | 0.000 | 0.5118 | No |
| 726 | ENSG00000216977 | RPL21P65 |  |  | 27735 | 0.000 | 0.5117 | No |
| 727 | ENSG00000243265 | RPL23AP55 |  |  | 27766 | 0.000 | 0.5112 | No |
| 728 | ENSG00000214374 | RPLP2P1 |  |  | 27769 | 0.000 | 0.5111 | No |
| 729 | ENSG00000236330 | RPL5P9 |  |  | 27799 | 0.000 | 0.5106 | No |
| 730 | ENSG00000250622 | AC093325.1 |  |  | 27846 | 0.000 | 0.5098 | No |
| 731 | ENSG00000225591 | BX248409.1 |  |  | 27861 | 0.000 | 0.5095 | No |
| 732 | ENSG00000216813 | AL358178.1 |  |  | 27872 | 0.000 | 0.5094 | No |
| 733 | ENSG00000224839 | RPL12P17 |  |  | 27941 | 0.000 | 0.5082 | No |
| 734 | ENSG00000262959 | RPL23AP86 |  |  | 27945 | 0.000 | 0.5081 | No |
| 735 | ENSG00000242661 | RPS3AP43 |  |  | 27977 | 0.000 | 0.5076 | No |
| 736 | ENSG00000238180 | AC017079.2 |  |  | 27982 | 0.000 | 0.5075 | No |
| 737 | ENSG00000216548 | AL357084.1 |  |  | 27983 | 0.000 | 0.5075 | No |
| 738 | ENSG00000214203 | RPS4XP1 |  |  | 27986 | 0.000 | 0.5075 | No |
| 739 | ENSG00000239393 | AC146507.1 |  |  | 27988 | 0.000 | 0.5075 | No |
| 740 | ENSG00000213669 | AL137074.1 |  |  | 27991 | 0.000 | 0.5074 | No |
| 741 | ENSG00000239215 | RPL27P12 |  |  | 28042 | 0.000 | 0.5065 | No |
| 742 | ENSG00000229468 | RPL39P18 |  |  | 28045 | 0.000 | 0.5065 | No |
| 743 | ENSG00000226701 | RPL15P14 |  |  | 28051 | 0.000 | 0.5064 | No |
| 744 | ENSG00000242727 | AC104687.1 |  |  | 28058 | 0.000 | 0.5063 | No |
| 745 | ENSG00000229700 | AL008627.1 |  |  | 28085 | 0.000 | 0.5058 | No |
| 746 | ENSG00000214289 | RPL39P5 |  |  | 28123 | 0.000 | 0.5052 | No |
| 747 | ENSG00000236073 | AL627311.1 |  |  | 28127 | 0.000 | 0.5051 | No |
| 748 | ENSG00000239524 | RPL32P34 |  |  | 28132 | 0.000 | 0.5051 | No |
| 749 | ENSG00000213876 | RPL7AP64 |  |  | 28192 | 0.000 | 0.5040 | No |
| 750 | ENSG00000270800 | RPS10-NUDT3 |  |  | 28255 | 0.000 | 0.5029 | No |
| 751 | ENSG00000220744 | RPL5P18 |  |  | 28259 | 0.000 | 0.5029 | No |
| 752 | ENSG00000235440 | AC011742.3 |  |  | 28306 | 0.000 | 0.5021 | No |
| 753 | ENSG00000232493 | RPL12P11 |  |  | 28307 | 0.000 | 0.5021 | No |
| 754 | ENSG00000216471 | RPSAP43 |  |  | 28313 | 0.000 | 0.5020 | No |
| 755 | ENSG00000240167 | RPS7P7 |  |  | 28345 | 0.000 | 0.5014 | No |
| 756 | ENSG00000243607 | RPL35AP26 |  |  | 28391 | 0.000 | 0.5007 | No |
| 757 | ENSG00000240554 | RPL7AP58 |  |  | 28393 | 0.000 | 0.5006 | No |
| 758 | ENSG00000243234 | AC010326.1 |  |  | 28413 | 0.000 | 0.5003 | No |
| 759 | ENSG00000271343 | AL355598.1 |  |  | 28434 | 0.000 | 0.4999 | No |
| 760 | ENSG00000242882 | RPL5P11 |  |  | 28437 | 0.000 | 0.4999 | No |
| 761 | ENSG00000236905 | AC104333.3 |  |  | 28492 | 0.000 | 0.4990 | No |
| 762 | ENSG00000235267 | AC074117.2 |  |  | 28561 | 0.000 | 0.4978 | No |
| 763 | ENSG00000227401 | RPL37P1 |  |  | 28582 | 0.000 | 0.4974 | No |
| 764 | ENSG00000239473 | RPL7P38 |  |  | 28620 | 0.000 | 0.4968 | No |
| 765 | ENSG00000266959 | AC005786.1 |  |  | 28721 | 0.000 | 0.4950 | No |
| 766 | ENSG00000234277 | LINC01641 |  |  | 28738 | 0.000 | 0.4947 | No |
| 767 | ENSG00000234272 | RPL30P2 |  |  | 28739 | 0.000 | 0.4947 | No |
| 768 | ENSG00000242706 | RPS27AP9 |  |  | 28778 | 0.000 | 0.4940 | No |
| 769 | ENSG00000262623 | AC004584.2 |  |  | 28784 | 0.000 | 0.4940 | No |
| 770 | ENSG00000243175 | RPSAP36 |  |  | 28818 | 0.000 | 0.4934 | No |
| 771 | ENSG00000243280 | AC093663.2 |  |  | 28822 | 0.000 | 0.4933 | No |
| 772 | ENSG00000241464 | RPL39P38 |  |  | 28825 | 0.000 | 0.4933 | No |
| 773 | ENSG00000213307 | RPL18P11 |  |  | 28849 | 0.000 | 0.4929 | No |
| 774 | ENSG00000218643 | RPL5P20 |  |  | 28876 | 0.000 | 0.4924 | No |
| 775 | ENSG00000240305 | AC091152.1 |  |  | 28897 | 0.000 | 0.4921 | No |
| 776 | ENSG00000244535 | AL049714.1 |  |  | 28938 | 0.000 | 0.4914 | No |
| 777 | ENSG00000227399 | AL391219.1 |  |  | 28941 | 0.000 | 0.4913 | No |
| 778 | ENSG00000243312 | AC093827.1 |  |  | 28985 | 0.000 | 0.4906 | No |
| 779 | ENSG00000214093 | AL096701.1 |  |  | 28997 | 0.000 | 0.4904 | No |
| 780 | ENSG00000240974 | RPS27AP10 |  |  | 29001 | 0.000 | 0.4903 | No |
| 781 | ENSG00000240808 | AC126389.1 |  |  | 29030 | 0.000 | 0.4898 | No |
| 782 | ENSG00000249509 | AC023886.1 |  |  | 29066 | 0.000 | 0.4892 | No |
| 783 | ENSG00000269637 | RPL12P41 |  |  | 29122 | 0.000 | 0.4883 | No |
| 784 | ENSG00000243129 | AC023790.1 |  |  | 29127 | 0.000 | 0.4882 | No |
| 785 | ENSG00000227852 | RPS29P17 |  |  | 29176 | 0.000 | 0.4873 | No |
| 786 | ENSG00000236844 | AC069213.3 |  |  | 29204 | 0.000 | 0.4869 | No |
| 787 | ENSG00000243417 | AC023424.2 |  |  | 29220 | 0.000 | 0.4866 | No |
| 788 | ENSG00000243353 | RPS29P19 |  |  | 29241 | 0.000 | 0.4862 | No |
| 789 | ENSG00000214024 | RPL23AP29 |  |  | 29247 | 0.000 | 0.4862 | No |
| 790 | ENSG00000224476 | RPL36P16 |  |  | 29349 | 0.000 | 0.4844 | No |
| 791 | ENSG00000241003 | AC108860.1 |  |  | 29414 | 0.000 | 0.4833 | No |
| 792 | ENSG00000223505 | AC114402.1 |  |  | 29438 | 0.000 | 0.4828 | No |
| 793 | ENSG00000242527 | AC100771.1 |  |  | 29454 | 0.000 | 0.4826 | No |
| 794 | ENSG00000229920 | RPS4XP5 |  |  | 29456 | 0.000 | 0.4826 | No |
| 795 | ENSG00000213109 | AL365223.1 |  |  | 29457 | 0.000 | 0.4826 | No |
| 796 | ENSG00000214593 | AL354710.1 |  |  | 29468 | 0.000 | 0.4824 | No |
| 797 | ENSG00000233609 | RPL10P19 |  |  | 29472 | 0.000 | 0.4823 | No |
| 798 | ENSG00000244753 | RPL15P21 |  |  | 29485 | 0.000 | 0.4821 | No |
| 799 | ENSG00000244756 | AL049874.2 |  |  | 29486 | 0.000 | 0.4821 | No |
| 800 | ENSG00000269600 | AC016629.2 |  |  | 29519 | 0.000 | 0.4816 | No |
| 801 | ENSG00000240874 | AC025271.1 |  |  | 29545 | 0.000 | 0.4811 | No |
| 802 | ENSG00000223583 | AL513365.1 |  |  | 29569 | 0.000 | 0.4807 | No |
| 803 | ENSG00000213394 | RPSAP46 |  |  | 29641 | 0.000 | 0.4795 | No |
| 804 | ENSG00000243422 | RPL23AP49 |  |  | 29643 | 0.000 | 0.4794 | No |
| 805 | ENSG00000233163 | RPS12P17 |  |  | 29668 | 0.000 | 0.4790 | No |
| 806 | ENSG00000213703 | AL138847.1 |  |  | 29669 | 0.000 | 0.4790 | No |
| 807 | ENSG00000229037 | BX323845.3 |  |  | 29684 | 0.000 | 0.4788 | No |
| 808 | ENSG00000255074 | AC018523.1 |  |  | 29703 | 0.000 | 0.4785 | No |
| 809 | ENSG00000227337 | RPL23AP43 |  |  | 29744 | 0.000 | 0.4778 | No |
| 810 | ENSG00000243802 | AC090589.1 |  |  | 29752 | 0.000 | 0.4776 | No |
| 811 | ENSG00000268884 | AC010323.2 |  |  | 29753 | 0.000 | 0.4776 | No |
| 812 | ENSG00000232059 | AL451007.1 |  |  | 29849 | 0.000 | 0.4760 | No |
| 813 | ENSG00000243537 | KC877373.1 |  |  | 29855 | 0.000 | 0.4759 | No |
| 814 | ENSG00000227625 | AL359510.1 |  |  | 29872 | 0.000 | 0.4756 | No |
| 815 | ENSG00000230166 | RPL35AP24 |  |  | 29892 | 0.000 | 0.4753 | No |
| 816 | ENSG00000229914 | AL138899.2 |  |  | 29894 | 0.000 | 0.4752 | No |
| 817 | ENSG00000243730 | RPL29P3 |  |  | 29896 | 0.000 | 0.4752 | No |
| 818 | ENSG00000271002 | AC129492.5 |  |  | 29903 | 0.000 | 0.4751 | No |
| 819 | ENSG00000235670 | RPL21P40 |  |  | 29921 | 0.000 | 0.4748 | No |
| 820 | ENSG00000227474 | RPL6P24 |  |  | 29935 | 0.000 | 0.4746 | No |
| 821 | ENSG00000237382 | RPL21P121 |  |  | 30023 | 0.000 | 0.4731 | No |
| 822 | ENSG00000241923 | RPL14P3 |  |  | 30034 | 0.000 | 0.4729 | No |
| 823 | ENSG00000231164 | RPL7P56 |  |  | 30078 | 0.000 | 0.4721 | No |
| 824 | ENSG00000242602 | AC008953.1 |  |  | 30101 | 0.000 | 0.4717 | No |
| 825 | ENSG00000242607 | RPS3AP34 |  |  | 30102 | 0.000 | 0.4717 | No |
| 826 | ENSG00000213979 | RPL7AP14 |  |  | 30125 | 0.000 | 0.4713 | No |
| 827 | ENSG00000240729 | AC146507.2 |  |  | 30150 | 0.000 | 0.4709 | No |
| 828 | ENSG00000244585 | RPL12P33 |  |  | 30162 | 0.000 | 0.4707 | No |
| 829 | ENSG00000236940 | AL589765.3 |  |  | 30213 | 0.000 | 0.4698 | No |
| 830 | ENSG00000239670 | AL355864.2 |  |  | 30215 | 0.000 | 0.4698 | No |
| 831 | ENSG00000242683 | RPL12P21 |  |  | 30219 | 0.000 | 0.4698 | No |
| 832 | ENSG00000243648 | AC109454.1 |  |  | 30230 | 0.000 | 0.4696 | No |
| 833 | ENSG00000268785 | RPL7P50 |  |  | 30281 | 0.000 | 0.4687 | No |
| 834 | ENSG00000235225 | AC016712.1 |  |  | 30287 | 0.000 | 0.4686 | No |
| 835 | ENSG00000176343 | RPL37AP8 |  |  | 30334 | 0.000 | 0.4678 | No |
| 836 | ENSG00000213557 | AC068050.1 |  |  | 30361 | 0.000 | 0.4674 | No |
| 837 | ENSG00000228884 | RPL23AP26 |  |  | 30364 | 0.000 | 0.4673 | No |
| 838 | ENSG00000241026 | AC105910.1 |  |  | 30375 | 0.000 | 0.4672 | No |
| 839 | ENSG00000244018 | RPL35P6 |  |  | 30376 | 0.000 | 0.4672 | No |
| 840 | ENSG00000239528 | RPS14P8 |  |  | 30418 | 0.000 | 0.4664 | No |
| 841 | ENSG00000241829 | RPL21P54 |  |  | 30474 | 0.000 | 0.4655 | No |
| 842 | ENSG00000226984 | AL035410.1 |  |  | 30488 | 0.000 | 0.4652 | No |
| 843 | ENSG00000213176 | RPL13P6 |  |  | 30532 | 0.000 | 0.4645 | No |
| 844 | ENSG00000231544 | RSL24D1P11 |  |  | 30545 | 0.000 | 0.4643 | No |
| 845 | ENSG00000231546 | RPL3P5 |  |  | 30546 | 0.000 | 0.4643 | No |
| 846 | ENSG00000243402 | AC022973.1 |  |  | 30574 | 0.000 | 0.4638 | No |
| 847 | ENSG00000240925 | RPS20P31 |  |  | 30590 | 0.000 | 0.4635 | No |
| 848 | ENSG00000233106 | RPL12P3 |  |  | 30598 | 0.000 | 0.4634 | No |
| 849 | ENSG00000271215 | RPL21P136 |  |  | 30599 | 0.000 | 0.4634 | No |
| 850 | ENSG00000223730 | Z83838.1 |  |  | 30648 | 0.000 | 0.4626 | No |
| 851 | ENSG00000226082 | AC096915.1 |  |  | 30651 | 0.000 | 0.4625 | No |
| 852 | ENSG00000240441 | AC007622.1 |  |  | 30664 | 0.000 | 0.4623 | No |
| 853 | ENSG00000240454 | RPL39P26 |  |  | 30667 | 0.000 | 0.4623 | No |
| 854 | ENSG00000234718 | AC007161.1 |  |  | 30735 | 0.000 | 0.4611 | No |
| 855 | ENSG00000270549 | AL451044.1 |  |  | 30737 | 0.000 | 0.4611 | No |
| 856 | ENSG00000215472 | RPL17-C18orf32 |  |  | 30807 | 0.000 | 0.4599 | No |
| 857 | ENSG00000250562 | RPL38P4 |  |  | 30855 | NaN | 0.4590 | No |
| 858 | ENSG00000259160 | AL049869.4 |  |  | 30878 | NaN | 0.4587 | No |
| 859 | ENSG00000213851 | AC098590.1 |  |  | 30882 | NaN | 0.4586 | No |
| 860 | ENSG00000226331 | RPL23AP28 |  |  | 30924 | NaN | 0.4579 | No |
| 861 | ENSG00000236425 | AC069213.2 |  |  | 31023 | NaN | 0.4562 | No |
| 862 | ENSG00000234031 | RPS3AP44 |  |  | 31046 | NaN | 0.4558 | No |
| 863 | ENSG00000248843 | AL645949.2 |  |  | 31073 | NaN | 0.4554 | No |
| 864 | ENSG00000227224 | AC092035.1 |  |  | 31084 | NaN | 0.4552 | No |
| 865 | ENSG00000217026 | RPL10P1 |  |  | 31154 | NaN | 0.4540 | No |
| 866 | ENSG00000217684 | RPS3AP24 |  |  | 31172 | NaN | 0.4537 | No |
| 867 | ENSG00000229567 | AL139421.1 |  |  | 31185 | NaN | 0.4535 | No |
| 868 | ENSG00000226646 | RPL7P37 |  |  | 31241 | NaN | 0.4525 | No |
| 869 | ENSG00000239924 | RPL29P22 |  |  | 31251 | NaN | 0.4524 | No |
| 870 | ENSG00000233921 | RPS15AP40 |  |  | 31254 | NaN | 0.4524 | No |
| 871 | ENSG00000241257 | AC124945.1 |  |  | 31268 | NaN | 0.4521 | No |
| 872 | ENSG00000241251 | AC090525.1 |  |  | 31287 | NaN | 0.4518 | No |
| 873 | ENSG00000235804 | AL357078.2 |  |  | 31293 | NaN | 0.4517 | No |
| 874 | ENSG00000242747 | AC090515.1 |  |  | 31328 | NaN | 0.4512 | No |
| 875 | ENSG00000230384 | RPSAP25 |  |  | 31336 | NaN | 0.4510 | No |
| 876 | ENSG00000240068 | RPL21P42 |  |  | 31352 | NaN | 0.4508 | No |
| 877 | ENSG00000241334 | RPL35AP33 |  |  | 31383 | NaN | 0.4503 | No |
| 878 | ENSG00000268993 | RPL22P6 |  |  | 31385 | NaN | 0.4503 | No |
| 879 | ENSG00000235544 | RPS11P1 |  |  | 31409 | NaN | 0.4499 | No |
| 880 | ENSG00000215369 | RPL37P3 |  |  | 31474 | NaN | 0.4487 | No |
| 881 | ENSG00000226339 | RPS26P56 |  |  | 31485 | NaN | 0.4486 | No |
| 882 | ENSG00000267408 | AC011498.5 |  |  | 31513 | NaN | 0.4481 | No |
| 883 | ENSG00000223903 | RPL23P4 |  |  | 31515 | NaN | 0.4481 | No |
| 884 | ENSG00000218208 | RPS27AP11 |  |  | 31518 | NaN | 0.4481 | No |
| 885 | ENSG00000242318 | AC058823.1 |  |  | 31519 | NaN | 0.4481 | No |
| 886 | ENSG00000242314 | RPL12P32 |  |  | 31520 | NaN | 0.4481 | No |
| 887 | ENSG00000239344 | AC090686.1 |  |  | 31536 | NaN | 0.4478 | No |
| 888 | ENSG00000270378 | AC020891.4 |  |  | 31616 | NaN | 0.4464 | No |
| 889 | ENSG00000244249 | AC063955.1 |  |  | 31669 | NaN | 0.4455 | No |
| 890 | ENSG00000230480 | AC093142.1 |  |  | 31688 | NaN | 0.4452 | No |
| 891 | ENSG00000271543 | AC021443.1 |  |  | 31701 | NaN | 0.4450 | No |
| 892 | ENSG00000236111 | AL603882.1 |  |  | 31705 | NaN | 0.4450 | No |
| 893 | ENSG00000236807 | AC092066.1 |  |  | 31750 | NaN | 0.4442 | No |
| 894 | ENSG00000225722 | BX890604.2 |  |  | 31792 | NaN | 0.4435 | No |
| 895 | ENSG00000244432 | RPL39P28 |  |  | 31806 | NaN | 0.4433 | No |
| 896 | ENSG00000258613 | RPL21P7 |  |  | 31829 | NaN | 0.4429 | No |
| 897 | ENSG00000258618 | RPL21P9 |  |  | 31830 | NaN | 0.4429 | No |
| 898 | ENSG00000255763 | MRPS18CP4 |  |  | 31853 | NaN | 0.4425 | No |
| 899 | ENSG00000242293 | RPS29P1 |  |  | 31860 | NaN | 0.4424 | No |
| 900 | ENSG00000242292 | AC010631.1 |  |  | 31861 | NaN | 0.4424 | No |
| 901 | ENSG00000242291 | RPL36AP51 |  |  | 31862 | NaN | 0.4424 | No |
| 902 | ENSG00000253646 | AC010476.1 |  |  | 31873 | NaN | 0.4423 | No |
| 903 | ENSG00000230779 | AL161636.2 |  |  | 31882 | NaN | 0.4421 | No |
| 904 | ENSG00000243988 | RPS24P17 |  |  | 31894 | NaN | 0.4420 | No |
| 905 | ENSG00000216718 | AL024509.2 |  |  | 31900 | NaN | 0.4419 | No |
| 906 | ENSG00000237205 | RPL7P34 |  |  | 31916 | NaN | 0.4416 | No |
| 907 | ENSG00000240180 | RPL12P28 |  |  | 31921 | NaN | 0.4416 | No |
| 908 | ENSG00000240861 | AC009153.1 |  |  | 31923 | NaN | 0.4416 | No |
| 909 | ENSG00000239315 | RPL19P13 |  |  | 31933 | NaN | 0.4414 | No |
| 910 | ENSG00000270780 | AL591742.1 |  |  | 31945 | NaN | 0.4412 | No |
| 911 | ENSG00000271705 | RPL17P3 |  |  | 31953 | NaN | 0.4411 | No |
| 912 | ENSG00000240975 | AC021723.1 |  |  | 32015 | NaN | 0.4400 | No |
| 913 | ENSG00000224796 | RPL32P1 |  |  | 32021 | NaN | 0.4400 | No |
| 914 | ENSG00000271250 | AL158032.1 |  |  | 32076 | NaN | 0.4390 | No |
| 915 | ENSG00000214064 | RPL6P5 |  |  | 32089 | NaN | 0.4388 | No |
| 916 | ENSG00000233158 | RPS24P6 |  |  | 32095 | NaN | 0.4387 | No |
| 917 | ENSG00000219863 | RPL31P28 |  |  | 32109 | NaN | 0.4385 | No |
| 918 | ENSG00000242358 | RPS21P4 |  |  | 32137 | NaN | 0.4380 | No |
| 919 | ENSG00000254305 | MRPL9P1 |  |  | 32138 | NaN | 0.4381 | No |
| 920 | ENSG00000228085 | AC009296.1 |  |  | 32155 | NaN | 0.4378 | No |
| 921 | ENSG00000184423 | RPL23AP38 |  |  | 32169 | NaN | 0.4376 | No |
| 922 | ENSG00000225912 | AL121871.1 |  |  | 32193 | NaN | 0.4372 | No |
| 923 | ENSG00000225918 | RPL7P59 |  |  | 32197 | NaN | 0.4371 | No |
| 924 | ENSG00000229022 | AL365366.1 |  |  | 32198 | NaN | 0.4371 | No |
| 925 | ENSG00000227514 | AC107890.1 |  |  | 32204 | NaN | 0.4370 | No |
| 926 | ENSG00000244662 | AC087312.1 |  |  | 32212 | NaN | 0.4369 | No |
| 927 | ENSG00000244669 | AC104689.1 |  |  | 32213 | NaN | 0.4369 | No |
| 928 | ENSG00000228860 | AL731547.1 |  |  | 32247 | NaN | 0.4364 | No |
| 929 | ENSG00000228861 | RPL23AP12 |  |  | 32248 | NaN | 0.4364 | No |
| 930 | ENSG00000231302 | RPL36P2 |  |  | 32256 | NaN | 0.4363 | No |
| 931 | ENSG00000231301 | RPL13AP |  |  | 32257 | NaN | 0.4363 | No |
| 932 | ENSG00000255535 | AP003041.2 |  |  | 32278 | NaN | 0.4359 | No |
| 933 | ENSG00000239870 | AC112206.1 |  |  | 32306 | NaN | 0.4355 | No |
| 934 | ENSG00000271268 | AC015849.6 |  |  | 32346 | NaN | 0.4348 | No |
| 935 | ENSG00000270865 | RPL12P48 |  |  | 32349 | NaN | 0.4348 | No |
| 936 | ENSG00000269888 | AC112491.1 |  |  | 32420 | NaN | 0.4335 | No |
| 937 | ENSG00000232627 | AC021876.1 |  |  | 32441 | NaN | 0.4332 | No |
| 938 | ENSG00000213488 | AC008716.1 |  |  | 32451 | NaN | 0.4330 | No |
| 939 | ENSG00000213486 | AC108039.1 |  |  | 32454 | NaN | 0.4330 | No |
| 940 | ENSG00000214073 | RPL21P17 |  |  | 32455 | NaN | 0.4330 | No |
| 941 | ENSG00000242423 | RPL12P36 |  |  | 32463 | NaN | 0.4329 | No |
| 942 | ENSG00000223987 | RPL26P28 |  |  | 32468 | NaN | 0.4328 | No |
| 943 | ENSG00000242390 | RPL6P9 |  |  | 32481 | NaN | 0.4326 | No |
| 944 | ENSG00000242399 | RPS20P23 |  |  | 32485 | NaN | 0.4326 | No |
| 945 | ENSG00000218173 | AL356967.1 |  |  | 32494 | NaN | 0.4325 | No |
| 946 | ENSG00000244400 | RPS4XP12 |  |  | 32508 | NaN | 0.4322 | No |
| 947 | ENSG00000234806 | RPL26P29 |  |  | 32510 | NaN | 0.4322 | No |
| 948 | ENSG00000242444 | AC007207.1 |  |  | 32529 | NaN | 0.4319 | No |
| 949 | ENSG00000271456 | AC108081.1 |  |  | 32543 | NaN | 0.4317 | No |
| 950 | ENSG00000230194 | AC000081.1 |  |  | 32561 | NaN | 0.4314 | No |
| 951 | ENSG00000204196 | RPL12P16 |  |  | 32576 | NaN | 0.4312 | No |
| 952 | ENSG00000204194 | RPL12P1 |  |  | 32577 | NaN | 0.4312 | No |
| 953 | ENSG00000239840 | RPL23AP72 |  |  | 32585 | NaN | 0.4311 | No |
| 954 | ENSG00000230353 | AC114755.3 |  |  | 32601 | NaN | 0.4308 | No |
| 955 | ENSG00000227309 | AC140076.1 |  |  | 32624 | NaN | 0.4304 | No |
| 956 | ENSG00000243167 | RPS10P28 |  |  | 32673 | NaN | 0.4296 | No |
| 957 | ENSG00000243164 | AC087879.1 |  |  | 32674 | NaN | 0.4296 | No |
| 958 | ENSG00000243160 | AC093270.2 |  |  | 32677 | NaN | 0.4296 | No |
| 959 | ENSG00000243297 | RPL31P61 |  |  | 32695 | NaN | 0.4293 | No |
| 960 | ENSG00000277447 | AP005431.1 |  |  | 32721 | NaN | 0.4288 | No |
| 961 | ENSG00000236735 | RPL31P63 |  |  | 32765 | NaN | 0.4281 | No |
| 962 | ENSG00000235420 | RPL29P30 |  |  | 32770 | NaN | 0.4280 | No |
| 963 | ENSG00000235429 | AC083875.1 |  |  | 32775 | NaN | 0.4280 | No |
| 964 | ENSG00000236888 | RPS20P5 |  |  | 32782 | NaN | 0.4279 | No |
| 965 | ENSG00000234488 | AC096664.2 |  |  | 32811 | NaN | 0.4274 | No |
| 966 | ENSG00000226279 | RPL12P10 |  |  | 32839 | NaN | 0.4269 | No |
| 967 | ENSG00000185631 | AC017079.1 |  |  | 32859 | NaN | 0.4266 | No |
| 968 | ENSG00000254850 | AC004923.2 |  |  | 32902 | NaN | 0.4259 | No |
| 969 | ENSG00000228360 | AC009220.1 |  |  | 32906 | NaN | 0.4258 | No |
| 970 | ENSG00000239261 | RPL31P41 |  |  | 32912 | NaN | 0.4257 | No |
| 971 | ENSG00000213312 | RPL7AP34 |  |  | 32935 | NaN | 0.4254 | No |
| 972 | ENSG00000260514 | AC009086.1 |  |  | 32948 | NaN | 0.4252 | No |
| 973 | ENSG00000239465 | AC090543.1 |  |  | 32957 | NaN | 0.4250 | No |
| 974 | ENSG00000256627 | AC073651.2 |  |  | 33044 | NaN | 0.4235 | No |
| 975 | ENSG00000231494 | AC104634.1 |  |  | 33055 | NaN | 0.4234 | No |
| 976 | ENSG00000214917 | AC011825.1 |  |  | 33076 | NaN | 0.4230 | No |
| 977 | ENSG00000214914 | RPL23AP3 |  |  | 33082 | NaN | 0.4229 | No |
| 978 | ENSG00000271429 | RPL17P51 |  |  | 33116 | NaN | 0.4224 | No |
| 979 | ENSG00000188850 | AC114947.1 |  |  | 33117 | NaN | 0.4224 | No |
| 980 | ENSG00000229032 | AL445685.2 |  |  | 33126 | NaN | 0.4222 | No |
| 981 | ENSG00000270776 | AC012443.1 |  |  | 33146 | NaN | 0.4219 | No |
| 982 | ENSG00000234524 | RPL12P43 |  |  | 33162 | NaN | 0.4217 | No |
| 983 | ENSG00000225443 | AC004938.1 |  |  | 33185 | NaN | 0.4213 | No |
| 984 | ENSG00000229753 | RPS27AP15 |  |  | 33190 | NaN | 0.4212 | No |
| 985 | ENSG00000229752 | RPL7P10 |  |  | 33191 | NaN | 0.4212 | No |
| 986 | ENSG00000229757 | AL031736.2 |  |  | 33193 | NaN | 0.4212 | No |
| 987 | ENSG00000229756 | RPL31P20 |  |  | 33194 | NaN | 0.4212 | No |
| 988 | ENSG00000225991 | RPL23AP34 |  |  | 33206 | NaN | 0.4210 | No |
| 989 | ENSG00000215450 | AL022342.1 |  |  | 33217 | NaN | 0.4209 | No |
| 990 | ENSG00000217327 | RPS7P5 |  |  | 33280 | NaN | 0.4198 | No |
| 991 | ENSG00000243664 | RPS29P12 |  |  | 33305 | NaN | 0.4194 | No |
| 992 | ENSG00000243663 | RPS4XP14 |  |  | 33307 | NaN | 0.4194 | No |
| 993 | ENSG00000243669 | RPL34P23 |  |  | 33308 | NaN | 0.4194 | No |
| 994 | ENSG00000233717 | RPS29P29 |  |  | 33383 | NaN | 0.4181 | No |
| 995 | ENSG00000231029 | AL031733.1 |  |  | 33414 | NaN | 0.4176 | No |
| 996 | ENSG00000231316 | AL591419.1 |  |  | 33422 | NaN | 0.4174 | No |
| 997 | ENSG00000230993 | RPL12P15 |  |  | 33430 | NaN | 0.4173 | No |
| 998 | ENSG00000230370 | RPL23AP52 |  |  | 33444 | NaN | 0.4171 | No |
| 999 | ENSG00000235828 | RPL36AP26 |  |  | 33459 | NaN | 0.4169 | No |
| 1000 | ENSG00000243504 | RPS23P1 |  |  | 33461 | NaN | 0.4169 | No |
| 1001 | ENSG00000240328 | AC091805.1 |  |  | 33541 | NaN | 0.4155 | No |
| 1002 | ENSG00000240775 | AC021205.1 |  |  | 33546 | NaN | 0.4154 | No |
| 1003 | ENSG00000240846 | RPS15P9 |  |  | 33560 | NaN | 0.4152 | No |
| 1004 | ENSG00000267135 | AD000091.1 |  |  | 33593 | NaN | 0.4146 | No |
| 1005 | ENSG00000259674 | AC092868.1 |  |  | 33630 | NaN | 0.4140 | No |
| 1006 | ENSG00000244053 | RPL13AP2 |  |  | 33650 | NaN | 0.4137 | No |
| 1007 | ENSG00000241744 | AC079953.1 |  |  | 33704 | NaN | 0.4128 | No |
| 1008 | ENSG00000241746 | AC104212.2 |  |  | 33705 | NaN | 0.4128 | No |
| 1009 | ENSG00000240540 | RPL3P9 |  |  | 33719 | NaN | 0.4125 | No |
| 1010 | ENSG00000240738 | AF279873.2 |  |  | 33720 | NaN | 0.4126 | No |
| 1011 | ENSG00000215278 | RPS7P6 |  |  | 33753 | NaN | 0.4120 | No |
| 1012 | ENSG00000224439 | RPSAP10 |  |  | 33761 | NaN | 0.4119 | No |
| 1013 | ENSG00000224438 | RPL23AP14 |  |  | 33762 | NaN | 0.4119 | No |
| 1014 | ENSG00000284670 | AC092128.1 |  |  | 33815 | NaN | 0.4110 | No |
| 1015 | ENSG00000214889 | RPS9P1 |  |  | 33821 | NaN | 0.4109 | No |
| 1016 | ENSG00000180172 | RPS12P23 |  |  | 33825 | NaN | 0.4109 | No |
| 1017 | ENSG00000219902 | RPL35P3 |  |  | 33832 | NaN | 0.4108 | No |
| 1018 | ENSG00000237068 | RPL5P2 |  |  | 33858 | NaN | 0.4103 | No |
| 1019 | ENSG00000239200 | RPL22P20 |  |  | 33893 | NaN | 0.4097 | No |
| 1020 | ENSG00000214457 | RPL7P29 |  |  | 33895 | NaN | 0.4097 | No |
| 1021 | ENSG00000213393 | AC091980.1 |  |  | 33921 | NaN | 0.4093 | No |
| 1022 | ENSG00000226489 | AC104308.1 |  |  | 33955 | NaN | 0.4087 | No |
| 1023 | ENSG00000228462 | RPS19P7 |  |  | 33963 | NaN | 0.4086 | No |
| 1024 | ENSG00000225286 | AC005105.1 |  |  | 33969 | NaN | 0.4085 | No |
| 1025 | ENSG00000225823 | RPL7P45 |  |  | 33988 | NaN | 0.4082 | No |
| 1026 | ENSG00000244717 | RPS27P14 |  |  | 34007 | NaN | 0.4079 | No |
| 1027 | ENSG00000243396 | AC084198.1 |  |  | 34058 | NaN | 0.4070 | No |
| 1028 | ENSG00000253945 | AC024995.1 |  |  | 34067 | NaN | 0.4069 | No |
| 1029 | ENSG00000253946 | AC008489.1 |  |  | 34069 | NaN | 0.4069 | No |
| 1030 | ENSG00000238150 | AC008753.1 |  |  | 34073 | NaN | 0.4069 | No |
| 1031 | ENSG00000237109 | RPL21P111 |  |  | 34116 | NaN | 0.4061 | No |
| 1032 | ENSG00000236477 | RPS24P1 |  |  | 34131 | NaN | 0.4059 | No |
| 1033 | ENSG00000230867 | AL157884.1 |  |  | 34171 | NaN | 0.4052 | No |
| 1034 | ENSG00000226132 | RPS3AP46 |  |  | 34197 | NaN | 0.4048 | No |
| 1035 | ENSG00000242088 | AC090602.1 |  |  | 34248 | NaN | 0.4039 | No |
| 1036 | ENSG00000242080 | AC008571.1 |  |  | 34251 | NaN | 0.4039 | No |
| 1037 | ENSG00000218596 | AL162578.1 |  |  | 34274 | NaN | 0.4035 | No |
| 1038 | ENSG00000223877 | RPS8P10 |  |  | 34279 | NaN | 0.4034 | No |
| 1039 | ENSG00000213216 | AC007066.1 |  |  | 34285 | NaN | 0.4034 | No |
| 1040 | ENSG00000239780 | RPLP0P11 |  |  | 34323 | NaN | 0.4027 | No |
| 1041 | ENSG00000233979 | RPL22P21 |  |  | 34343 | NaN | 0.4024 | No |
| 1042 | ENSG00000233971 | RPS20P10 |  |  | 34347 | NaN | 0.4023 | No |
| 1043 | ENSG00000240159 | AC079080.1 |  |  | 34372 | NaN | 0.4019 | No |
| 1044 | ENSG00000234566 | RPL7AP71 |  |  | 34391 | NaN | 0.4016 | No |
| 1045 | ENSG00000234637 | RPS19P6 |  |  | 34396 | NaN | 0.4016 | No |
| 1046 | ENSG00000244192 | AC113367.1 |  |  | 34414 | NaN | 0.4013 | No |
| 1047 | ENSG00000241870 | AC008620.1 |  |  | 34415 | NaN | 0.4013 | No |
| 1048 | ENSG00000241877 | AC095041.1 |  |  | 34416 | NaN | 0.4013 | No |
| 1049 | ENSG00000219559 | AL512430.3 |  |  | 34428 | NaN | 0.4011 | No |
| 1050 | ENSG00000248200 | AC093770.1 |  |  | 34447 | NaN | 0.4008 | No |
| 1051 | ENSG00000240052 | AC108114.1 |  |  | 34451 | NaN | 0.4007 | No |
| 1052 | ENSG00000214255 | RPS2P6 |  |  | 34470 | NaN | 0.4004 | No |
| 1053 | ENSG00000232723 | AC013399.1 |  |  | 34481 | NaN | 0.4003 | No |
| 1054 | ENSG00000218754 | RPL18AP8 |  |  | 34491 | NaN | 0.4001 | No |
| 1055 | ENSG00000216616 | AL356421.1 |  |  | 34494 | NaN | 0.4001 | No |
| 1056 | ENSG00000223540 | RPS15AP5 |  |  | 34500 | NaN | 0.4000 | No |
| 1057 | ENSG00000174418 | RPL35P9 |  |  | 34504 | NaN | 0.4000 | No |
| 1058 | ENSG00000224019 | RPL21P32 |  |  | 34520 | NaN | 0.3997 | No |
| 1059 | ENSG00000244237 | AL133445.1 |  |  | 34540 | NaN | 0.3994 | No |
| 1060 | ENSG00000229143 | AC009480.2 |  |  | 34544 | NaN | 0.3993 | No |
| 1061 | ENSG00000241281 | AC010683.1 |  |  | 34548 | NaN | 0.3993 | No |
| 1062 | ENSG00000259369 | AC009997.1 |  |  | 34562 | NaN | 0.3991 | No |
| 1063 | ENSG00000243499 | RPS6P21 |  |  | 34567 | NaN | 0.3990 | No |
| 1064 | ENSG00000213470 | AC073594.1 |  |  | 34594 | NaN | 0.3986 | No |
| 1065 | ENSG00000242321 | RPL23AP40 |  |  | 34628 | NaN | 0.3980 | No |
| 1066 | ENSG00000242327 | AC023906.1 |  |  | 34629 | NaN | 0.3980 | No |
| 1067 | ENSG00000242329 | AC007215.1 |  |  | 34631 | NaN | 0.3980 | No |
| 1068 | ENSG00000197575 | RPS17P2 |  |  | 34633 | NaN | 0.3980 | No |
| 1069 | ENSG00000229965 | AL359704.1 |  |  | 34640 | NaN | 0.3979 | No |
| 1070 | ENSG00000243744 | AC016546.1 |  |  | 34648 | NaN | 0.3978 | No |
| 1071 | ENSG00000240210 | AL122013.1 |  |  | 34679 | NaN | 0.3972 | No |
| 1072 | ENSG00000259545 | AC013452.1 |  |  | 34688 | NaN | 0.3971 | No |
| 1073 | ENSG00000232873 | RPL23AP93 |  |  | 34742 | NaN | 0.3962 | No |
| 1074 | ENSG00000241932 | AC092324.1 |  |  | 34748 | NaN | 0.3961 | No |
| 1075 | ENSG00000226336 | MRPS16P3 |  |  | 34756 | NaN | 0.3960 | No |
| 1076 | ENSG00000236148 | RPL23AP37 |  |  | 34777 | NaN | 0.3956 | No |
| 1077 | ENSG00000264217 | RPL35AP25 |  |  | 34781 | NaN | 0.3956 | No |
| 1078 | ENSG00000228657 | RPL23AP58 |  |  | 34795 | NaN | 0.3954 | No |
| 1079 | ENSG00000231788 | RPL31P50 |  |  | 34798 | NaN | 0.3954 | No |
| 1080 | ENSG00000217776 | AL138827.1 |  |  | 34801 | NaN | 0.3953 | No |
| 1081 | ENSG00000235729 | AL500522.2 |  |  | 34809 | NaN | 0.3952 | No |
| 1082 | ENSG00000251285 | AC097484.2 |  |  | 34826 | NaN | 0.3949 | No |
| 1083 | ENSG00000234457 | AC006960.1 |  |  | 34856 | NaN | 0.3944 | No |
| 1084 | ENSG00000213400 | RPL12P18 |  |  | 34864 | NaN | 0.3943 | No |
| 1085 | ENSG00000240601 | RPL9P15 |  |  | 34866 | NaN | 0.3943 | No |
| 1086 | ENSG00000227753 | AC023194.1 |  |  | 34903 | NaN | 0.3937 | No |
| 1087 | ENSG00000242829 | RPS26P21 |  |  | 34916 | NaN | 0.3935 | No |
| 1088 | ENSG00000266978 | AC011511.2 |  |  | 34944 | NaN | 0.3930 | No |
| 1089 | ENSG00000240627 | AC026798.1 |  |  | 35002 | NaN | 0.3920 | No |
| 1090 | ENSG00000237186 | AC092418.1 |  |  | 35046 | NaN | 0.3913 | No |
| 1091 | ENSG00000270488 | AC079587.1 |  |  | 35080 | NaN | 0.3907 | No |
| 1092 | ENSG00000242993 | AP000870.1 |  |  | 35113 | NaN | 0.3901 | No |
| 1093 | ENSG00000259695 | AC090164.1 |  |  | 35166 | NaN | 0.3892 | No |
| 1094 | ENSG00000218049 | RPL31P33 |  |  | 35176 | NaN | 0.3891 | No |
| 1095 | ENSG00000213744 | RPS10P14 |  |  | 35204 | NaN | 0.3886 | No |
| 1096 | ENSG00000223733 | RPL18AP14 |  |  | 35208 | NaN | 0.3886 | No |
| 1097 | ENSG00000223739 | AC007389.2 |  |  | 35219 | NaN | 0.3884 | No |
| 1098 | ENSG00000224706 | RPS17P13 |  |  | 35225 | NaN | 0.3883 | No |
| 1099 | ENSG00000213291 | AC010099.1 |  |  | 35226 | NaN | 0.3883 | No |
| 1100 | ENSG00000213293 | AC012618.1 |  |  | 35227 | NaN | 0.3883 | No |
| 1101 | ENSG00000213295 | RPL22P10 |  |  | 35229 | NaN | 0.3883 | No |
| 1102 | novel.278 | - |  |  | 35242 | NaN | 0.3881 | No |
| 1103 | ENSG00000239683 | AC007998.1 |  |  | 35266 | NaN | 0.3877 | No |
| 1104 | ENSG00000220131 | AL121835.1 |  |  | 35269 | NaN | 0.3877 | No |
| 1105 | ENSG00000225947 | AL596448.1 |  |  | 35300 | NaN | 0.3872 | No |
| 1106 | ENSG00000233291 | RPL7AP61 |  |  | 35309 | NaN | 0.3871 | No |
| 1107 | ENSG00000243053 | RPL31P58 |  |  | 35368 | NaN | 0.3860 | No |
| 1108 | ENSG00000243058 | RPL39P33 |  |  | 35374 | NaN | 0.3860 | No |
| 1109 | ENSG00000230559 | RPL17P12 |  |  | 35392 | NaN | 0.3857 | No |
| 1110 | ENSG00000248288 | AC008868.1 |  |  | 35414 | NaN | 0.3853 | No |
| 1111 | ENSG00000241367 | RPL7AP23 |  |  | 35427 | NaN | 0.3851 | No |
| 1112 | ENSG00000237024 | RPL21P33 |  |  | 35434 | NaN | 0.3850 | No |
| 1113 | ENSG00000270830 | AC022441.3 |  |  | 35450 | NaN | 0.3848 | No |
| 1114 | ENSG00000242995 | AL161757.1 |  |  | 35516 | NaN | 0.3836 | No |
| 1115 | ENSG00000242990 | RPL13AP23 |  |  | 35518 | NaN | 0.3836 | No |
| 1116 | ENSG00000240388 | AC008799.1 |  |  | 35533 | NaN | 0.3834 | No |
| 1117 | ENSG00000241130 | AC012100.1 |  |  | 35538 | NaN | 0.3833 | No |
| 1118 | ENSG00000227034 | AL445433.1 |  |  | 35565 | NaN | 0.3829 | No |
| 1119 | ENSG00000235605 | AL355472.2 |  |  | 35580 | NaN | 0.3826 | No |
| 1120 | ENSG00000260109 | AC135628.1 |  |  | 35604 | NaN | 0.3822 | No |
| 1121 | ENSG00000213157 | AC108734.1 |  |  | 35628 | NaN | 0.3818 | No |
| 1122 | ENSG00000220076 | AL034345.1 |  |  | 35652 | NaN | 0.3814 | No |
| 1123 | ENSG00000251334 | AC020593.1 |  |  | 35662 | NaN | 0.3813 | No |
| 1124 | ENSG00000225595 | LINC01623 |  |  | 35674 | NaN | 0.3811 | No |
| 1125 | ENSG00000244235 | AC103724.1 |  |  | 35679 | NaN | 0.3810 | No |
| 1126 | ENSG00000233839 | AL357055.1 |  |  | 35711 | NaN | 0.3805 | No |
| 1127 | ENSG00000226156 | RPL23AP76 |  |  | 35748 | NaN | 0.3799 | No |
| 1128 | ENSG00000248120 | AC114967.1 |  |  | 35809 | NaN | 0.3788 | No |
| 1129 | ENSG00000237924 | RPL21P112 |  |  | 35836 | NaN | 0.3784 | No |
| 1130 | ENSG00000233318 | RPL31P45 |  |  | 35837 | NaN | 0.3784 | No |
| 1131 | ENSG00000177452 | AL161742.1 |  |  | 35848 | NaN | 0.3782 | No |
| 1132 | ENSG00000214560 | RPL21P41 |  |  | 35849 | NaN | 0.3782 | No |
| 1133 | ENSG00000228780 | Z92545.1 |  |  | 35852 | NaN | 0.3782 | No |
| 1134 | ENSG00000241499 | AL132838.2 |  |  | 35858 | NaN | 0.3781 | No |
| 1135 | ENSG00000235414 | RPSAP24 |  |  | 35866 | NaN | 0.3780 | No |
| 1136 | ENSG00000244436 | AC023050.1 |  |  | 35867 | NaN | 0.3780 | No |
| 1137 | ENSG00000232199 | RPL3P8 |  |  | 35878 | NaN | 0.3778 | No |
| 1138 | ENSG00000215893 | RPL23AP17 |  |  | 35906 | NaN | 0.3774 | No |
| 1139 | ENSG00000225758 | RPS17P17 |  |  | 35915 | NaN | 0.3772 | No |
| 1140 | ENSG00000218351 | RPS3AP23 |  |  | 35991 | NaN | 0.3759 | No |
| 1141 | ENSG00000228992 | RPL5P32 |  |  | 35997 | NaN | 0.3758 | No |
| 1142 | ENSG00000242176 | RPL39P31 |  |  | 36005 | NaN | 0.3757 | No |
| 1143 | ENSG00000228331 | RPL17P43 |  |  | 36022 | NaN | 0.3755 | No |
| 1144 | ENSG00000237273 | RSL24D1P8 |  |  | 36034 | NaN | 0.3753 | No |
| 1145 | ENSG00000225251 | RPL5P25 |  |  | 36061 | NaN | 0.3748 | No |
| 1146 | ENSG00000255903 | RPL12P46 |  |  | 36079 | NaN | 0.3745 | No |
| 1147 | ENSG00000227950 | AL590128.1 |  |  | 36113 | NaN | 0.3740 | No |
| 1148 | ENSG00000271656 | AC007262.1 |  |  | 36132 | NaN | 0.3737 | No |
| 1149 | ENSG00000230249 | AC112653.1 |  |  | 36146 | NaN | 0.3734 | No |
| 1150 | ENSG00000237730 | AC078991.1 |  |  | 36192 | NaN | 0.3726 | No |
| 1151 | ENSG00000271415 | RPL23AP90 |  |  | 36219 | NaN | 0.3722 | No |
| 1152 | ENSG00000223782 | RPS21P8 |  |  | 36244 | NaN | 0.3718 | No |
| 1153 | ENSG00000220125 | MRPL32P1 |  |  | 36256 | NaN | 0.3716 | No |
| 1154 | ENSG00000223433 | RPS29P9 |  |  | 36262 | NaN | 0.3715 | No |
| 1155 | ENSG00000228030 | AC019084.1 |  |  | 36275 | NaN | 0.3713 | No |
| 1156 | ENSG00000228781 | AL353705.1 |  |  | 36291 | NaN | 0.3711 | No |
| 1157 | ENSG00000225499 | RPL15P4 |  |  | 36299 | NaN | 0.3709 | No |
| 1158 | ENSG00000243303 | AC103987.1 |  |  | 36316 | NaN | 0.3707 | No |
| 1159 | ENSG00000242692 | RPS27AP1 |  |  | 36358 | NaN | 0.3700 | No |
| 1160 | ENSG00000242697 | RPL5P12 |  |  | 36359 | NaN | 0.3700 | No |
| 1161 | ENSG00000242034 | AC097493.1 |  |  | 36395 | NaN | 0.3694 | No |
| 1162 | ENSG00000241678 | AC091564.1 |  |  | 36457 | NaN | 0.3683 | No |
| 1163 | ENSG00000217379 | AL589647.1 |  |  | 36461 | NaN | 0.3682 | No |
| 1164 | ENSG00000240813 | AC015908.1 |  |  | 36487 | NaN | 0.3678 | No |
| 1165 | ENSG00000241097 | AP001324.2 |  |  | 36488 | NaN | 0.3678 | No |
| 1166 | ENSG00000224573 | AC083863.1 |  |  | 36501 | NaN | 0.3676 | No |
| 1167 | ENSG00000235377 | AL158047.1 |  |  | 36504 | NaN | 0.3676 | No |
| 1168 | ENSG00000235378 | MRPS10P1 |  |  | 36506 | NaN | 0.3676 | No |
| 1169 | ENSG00000235379 | RPL7P31 |  |  | 36507 | NaN | 0.3676 | No |
| 1170 | ENSG00000244621 | RPS17P11 |  |  | 36508 | NaN | 0.3676 | No |
| 1171 | ENSG00000235817 | AL158214.1 |  |  | 36550 | NaN | 0.3669 | No |
| 1172 | ENSG00000238183 | RPS27P5 |  |  | 36582 | NaN | 0.3663 | No |
| 1173 | ENSG00000218872 | AL445189.1 |  |  | 36603 | NaN | 0.3660 | No |
| 1174 | ENSG00000225392 | AC016700.1 |  |  | 36608 | NaN | 0.3659 | No |
| 1175 | ENSG00000269244 | RPL23AP78 |  |  | 36620 | NaN | 0.3658 | No |
| 1176 | ENSG00000219993 | AL138878.1 |  |  | 36629 | NaN | 0.3656 | No |
| 1177 | ENSG00000228599 | RPL7P52 |  |  | 36652 | NaN | 0.3652 | No |
| 1178 | ENSG00000213613 | RPL11P3 |  |  | 36661 | NaN | 0.3651 | No |
| 1179 | ENSG00000258627 | AC103996.1 |  |  | 36676 | NaN | 0.3649 | No |
| 1180 | ENSG00000244551 | RPL34P29 |  |  | 36680 | NaN | 0.3648 | No |
| 1181 | ENSG00000244559 | AC091179.2 |  |  | 36681 | NaN | 0.3648 | No |
| 1182 | ENSG00000213075 | RPL31P11 |  |  | 36709 | NaN | 0.3644 | No |
| 1183 | ENSG00000231699 | AC020550.1 |  |  | 36752 | NaN | 0.3636 | No |
| 1184 | ENSG00000213058 | AL365357.1 |  |  | 36773 | NaN | 0.3633 | No |
| 1185 | ENSG00000241673 | RPS27P12 |  |  | 36774 | NaN | 0.3633 | No |
| 1186 | ENSG00000237347 | AC004461.1 |  |  | 36792 | NaN | 0.3630 | No |
| 1187 | ENSG00000224469 | AC009517.1 |  |  | 36838 | NaN | 0.3622 | No |
| 1188 | ENSG00000231930 | AF228730.1 |  |  | 36857 | NaN | 0.3619 | No |
| 1189 | ENSG00000242248 | RPL7AP57 |  |  | 36920 | NaN | 0.3608 | No |
| 1190 | ENSG00000238111 | AC066692.1 |  |  | 36944 | NaN | 0.3604 | No |
| 1191 | ENSG00000236243 | RPL6P29 |  |  | 36956 | NaN | 0.3603 | No |
| 1192 | ENSG00000242208 | RPL5P29 |  |  | 36974 | NaN | 0.3600 | No |
| 1193 | ENSG00000242209 | AC006445.1 |  |  | 36975 | NaN | 0.3600 | No |
| 1194 | ENSG00000213363 | RPS3P6 |  |  | 36986 | NaN | 0.3598 | No |
| 1195 | ENSG00000240426 | AC096743.1 |  |  | 36996 | NaN | 0.3597 | No |
| 1196 | ENSG00000237490 | RPS27AP17 |  |  | 37089 | NaN | 0.3580 | No |
| 1197 | ENSG00000240983 | AC090311.1 |  |  | 37093 | NaN | 0.3580 | No |
| 1198 | ENSG00000242729 | AC021006.1 |  |  | 37102 | NaN | 0.3579 | No |
| 1199 | ENSG00000243779 | AP001086.1 |  |  | 37117 | NaN | 0.3576 | No |
| 1200 | ENSG00000214743 | MRPS5P3 |  |  | 37158 | NaN | 0.3569 | No |
| 1201 | ENSG00000249970 | AC074250.1 |  |  | 37161 | NaN | 0.3569 | No |
| 1202 | ENSG00000253912 | AC018442.3 |  |  | 37168 | NaN | 0.3568 | No |
| 1203 | ENSG00000241562 | RPL7P5 |  |  | 37201 | NaN | 0.3563 | No |
| 1204 | ENSG00000220694 | AL096711.1 |  |  | 37263 | NaN | 0.3552 | No |
| 1205 | ENSG00000224161 | RPS26P54 |  |  | 37277 | NaN | 0.3550 | No |
| 1206 | ENSG00000219722 | RPL26P20 |  |  | 37320 | NaN | 0.3542 | No |
| 1207 | ENSG00000244485 | RPL18P13 |  |  | 37412 | NaN | 0.3526 | No |
| 1208 | ENSG00000225803 | MRPS11P1 |  |  | 37414 | NaN | 0.3526 | No |
| 1209 | ENSG00000240364 | RPL31P59 |  |  | 37415 | NaN | 0.3526 | No |
| 1210 | ENSG00000225354 | RPL7AP52 |  |  | 37439 | NaN | 0.3522 | No |
| 1211 | ENSG00000244061 | AC093278.1 |  |  | 37444 | NaN | 0.3522 | No |
| 1212 | ENSG00000242123 | AC053481.1 |  |  | 37472 | NaN | 0.3517 | No |
| 1213 | ENSG00000237676 | RPL30P4 |  |  | 37565 | NaN | 0.3501 | No |
| 1214 | ENSG00000248234 | AC091839.1 |  |  | 37568 | NaN | 0.3501 | No |
| 1215 | ENSG00000232751 | AL645568.3 |  |  | 37604 | NaN | 0.3495 | No |
| 1216 | ENSG00000223570 | AL592045.1 |  |  | 37627 | NaN | 0.3491 | No |
| 1217 | ENSG00000239659 | RPL9P31 |  |  | 37646 | NaN | 0.3488 | No |
| 1218 | ENSG00000228664 | AL513480.1 |  |  | 37652 | NaN | 0.3487 | No |
| 1219 | ENSG00000228665 | BX679664.1 |  |  | 37653 | NaN | 0.3487 | No |
| 1220 | ENSG00000239527 | RPS23P7 |  |  | 37656 | NaN | 0.3487 | No |
| 1221 | ENSG00000229133 | RPS7P4 |  |  | 37676 | NaN | 0.3484 | No |
| 1222 | ENSG00000227663 | RPL7P2 |  |  | 37704 | NaN | 0.3479 | No |
| 1223 | ENSG00000223968 | AC098614.2 |  |  | 37707 | NaN | 0.3479 | No |
| 1224 | ENSG00000223962 | UBBP3 |  |  | 37711 | NaN | 0.3478 | No |
| 1225 | ENSG00000217239 | AL136968.2 |  |  | 37743 | NaN | 0.3473 | No |
| 1226 | ENSG00000243771 | AC010618.1 |  |  | 37771 | NaN | 0.3468 | No |
| 1227 | ENSG00000243777 | AP000942.1 |  |  | 37772 | NaN | 0.3468 | No |
| 1228 | ENSG00000237475 | RPL7P53 |  |  | 37788 | NaN | 0.3466 | No |
| 1229 | ENSG00000205767 | AL136038.1 |  |  | 37807 | NaN | 0.3463 | No |
| 1230 | ENSG00000249520 | AC093824.1 |  |  | 37808 | NaN | 0.3463 | No |
| 1231 | ENSG00000241739 | AC010469.2 |  |  | 37814 | NaN | 0.3462 | No |
| 1232 | ENSG00000241011 | AC108073.2 |  |  | 37836 | NaN | 0.3458 | No |
| 1233 | ENSG00000213664 | RPS16P8 |  |  | 37852 | NaN | 0.3456 | No |
| 1234 | ENSG00000232228 | AC092431.2 |  |  | 37865 | NaN | 0.3454 | No |
| 1235 | ENSG00000232514 | AL356968.1 |  |  | 37867 | NaN | 0.3454 | No |
| 1236 | ENSG00000234109 | RPL7P36 |  |  | 37886 | NaN | 0.3450 | No |
| 1237 | ENSG00000224664 | RPL36AP53 |  |  | 37902 | NaN | 0.3448 | No |
| 1238 | ENSG00000215457 | RPS8P3 |  |  | 37919 | NaN | 0.3445 | No |
| 1239 | ENSG00000243916 | AC092183.1 |  |  | 37954 | NaN | 0.3439 | No |
| 1240 | ENSG00000243914 | RPL5P14 |  |  | 37959 | NaN | 0.3439 | No |
| 1241 | ENSG00000235110 | AC107421.1 |  |  | 37965 | NaN | 0.3438 | No |
| 1242 | ENSG00000213051 | RPL5P5 |  |  | 37969 | NaN | 0.3437 | No |
| 1243 | ENSG00000242807 | RPL26P31 |  |  | 37971 | NaN | 0.3437 | No |
| 1244 | ENSG00000227032 | RPS2P36 |  |  | 37977 | NaN | 0.3436 | No |
| 1245 | ENSG00000268074 | RPL22P5 |  |  | 38018 | NaN | 0.3430 | No |
| 1246 | ENSG00000225124 | RPL23AP36 |  |  | 38021 | NaN | 0.3429 | No |
| 1247 | ENSG00000226470 | RPS29P33 |  |  | 38024 | NaN | 0.3429 | No |
| 1248 | ENSG00000230283 | RPS12P24 |  |  | 38029 | NaN | 0.3428 | No |
| 1249 | ENSG00000230284 | AL160275.1 |  |  | 38032 | NaN | 0.3428 | No |
| 1250 | ENSG00000259105 | RPS3AP4 |  |  | 38059 | NaN | 0.3424 | No |
| 1251 | ENSG00000213875 | AC005019.1 |  |  | 38061 | NaN | 0.3424 | No |
| 1252 | ENSG00000227433 | AC090018.1 |  |  | 38084 | NaN | 0.3420 | No |
| 1253 | ENSG00000227437 | RPS8P4 |  |  | 38086 | NaN | 0.3420 | No |
| 1254 | ENSG00000228965 | RPSAP60 |  |  | 38110 | NaN | 0.3416 | No |
| 1255 | ENSG00000253621 | RPSAP74 |  |  | 38137 | NaN | 0.3411 | No |
| 1256 | ENSG00000253995 | RPL10P18 |  |  | 38147 | NaN | 0.3410 | No |
| 1257 | ENSG00000243929 | AC104784.1 |  |  | 38160 | NaN | 0.3408 | No |
| 1258 | ENSG00000243925 | RPS24P18 |  |  | 38163 | NaN | 0.3407 | No |
| 1259 | ENSG00000240698 | RPS2P39 |  |  | 38167 | NaN | 0.3407 | No |
| 1260 | ENSG00000274308 | AC244093.1 |  |  | 38182 | NaN | 0.3405 | No |
| 1261 | ENSG00000232166 | AL122008.2 |  |  | 38186 | NaN | 0.3404 | No |
| 1262 | ENSG00000240652 | AP001024.1 |  |  | 38252 | NaN | 0.3393 | No |
| 1263 | ENSG00000213210 | AC005229.2 |  |  | 38257 | NaN | 0.3392 | No |
| 1264 | ENSG00000239830 | RPS4XP22 |  |  | 38270 | NaN | 0.3390 | No |
| 1265 | ENSG00000244031 | RPS3AP17 |  |  | 38311 | NaN | 0.3383 | No |
| 1266 | ENSG00000213147 | RPL23AP60 |  |  | 38312 | NaN | 0.3383 | No |
| 1267 | ENSG00000235922 | AL354766.1 |  |  | 38327 | NaN | 0.3381 | No |
| 1268 | ENSG00000235926 | RPS29P15 |  |  | 38329 | NaN | 0.3381 | No |
| 1269 | ENSG00000213798 | AC004129.1 |  |  | 38387 | NaN | 0.3371 | No |
| 1270 | ENSG00000229500 | RPL17P1 |  |  | 38395 | NaN | 0.3370 | No |
| 1271 | ENSG00000237818 | RPS3AP29 |  |  | 38419 | NaN | 0.3366 | No |
| 1272 | ENSG00000228754 | RPL13AP19 |  |  | 38433 | NaN | 0.3363 | No |
| 1273 | ENSG00000226621 | AC083855.1 |  |  | 38442 | NaN | 0.3362 | No |
| 1274 | ENSG00000226624 | AC005099.1 |  |  | 38444 | NaN | 0.3362 | No |
| 1275 | ENSG00000229949 | AC005094.1 |  |  | 38458 | NaN | 0.3360 | No |
| 1276 | ENSG00000233752 | AC009414.1 |  |  | 38467 | NaN | 0.3358 | No |
| 1277 | ENSG00000241179 | AC010301.1 |  |  | 38485 | NaN | 0.3356 | No |
| 1278 | ENSG00000242640 | RPS29P11 |  |  | 38488 | NaN | 0.3355 | No |
| 1279 | ENSG00000215734 | MRPL20P1 |  |  | 38526 | NaN | 0.3349 | No |
| 1280 | ENSG00000219529 | AP000580.1 |  |  | 38553 | NaN | 0.3344 | No |
| 1281 | ENSG00000240083 | RPS3AP22 |  |  | 38555 | NaN | 0.3344 | No |
| 1282 | ENSG00000230507 | RPL7AP8 |  |  | 38558 | NaN | 0.3344 | No |
| 1283 | ENSG00000243686 | RPLP1P11 |  |  | 38561 | NaN | 0.3344 | No |
| 1284 | ENSG00000230508 | RPL19P21 |  |  | 38562 | NaN | 0.3344 | No |
| 1285 | ENSG00000241354 | AL049874.1 |  |  | 38585 | NaN | 0.3340 | No |
| 1286 | ENSG00000224594 | RPL29P19 |  |  | 38620 | NaN | 0.3334 | No |
| 1287 | ENSG00000234619 | RPL7P11 |  |  | 38634 | NaN | 0.3332 | No |
| 1288 | ENSG00000274493 | MRPS17P5 |  |  | 38665 | NaN | 0.3327 | No |
| 1289 | ENSG00000219776 | RPL21P67 |  |  | 38669 | NaN | 0.3326 | No |
| 1290 | ENSG00000180019 | AC079741.1 |  |  | 38678 | NaN | 0.3325 | No |
| 1291 | ENSG00000213421 | AC099670.1 |  |  | 38686 | NaN | 0.3324 | No |
| 1292 | ENSG00000218198 | RPS20P32 |  |  | 38695 | NaN | 0.3322 | No |
| 1293 | ENSG00000233159 | AC007390.1 |  |  | 38713 | NaN | 0.3320 | No |
| 1294 | ENSG00000241722 | AC083795.1 |  |  | 38714 | NaN | 0.3320 | No |
| 1295 | ENSG00000241723 | AC074276.1 |  |  | 38715 | NaN | 0.3320 | No |
| 1296 | ENSG00000233157 | Z97055.1 |  |  | 38720 | NaN | 0.3319 | No |
| 1297 | ENSG00000213169 | AC069218.1 |  |  | 38747 | NaN | 0.3315 | No |
| 1298 | ENSG00000240246 | AC108751.2 |  |  | 38749 | NaN | 0.3315 | No |
| 1299 | ENSG00000240298 | RPL36AP40 |  |  | 38772 | NaN | 0.3311 | No |
| 1300 | ENSG00000226016 | RPL36AP55 |  |  | 38808 | NaN | 0.3305 | No |
| 1301 | ENSG00000231017 | AC013404.1 |  |  | 38813 | NaN | 0.3304 | No |
| 1302 | ENSG00000241961 | AC011037.1 |  |  | 38866 | NaN | 0.3295 | No |
| 1303 | ENSG00000241965 | RPS2P25 |  |  | 38869 | NaN | 0.3295 | No |
| 1304 | ENSG00000224776 | RPSAP50 |  |  | 38881 | NaN | 0.3293 | No |
| 1305 | ENSG00000233329 | AC068287.1 |  |  | 38926 | NaN | 0.3285 | No |
| 1306 | ENSG00000232145 | AC099548.1 |  |  | 38942 | NaN | 0.3283 | No |
| 1307 | ENSG00000236863 | RPL23AP23 |  |  | 38969 | NaN | 0.3278 | No |
| 1308 | ENSG00000236862 | RPS20P24 |  |  | 38970 | NaN | 0.3278 | No |
| 1309 | ENSG00000236860 | RPL39P29 |  |  | 38971 | NaN | 0.3278 | No |
| 1310 | ENSG00000242850 | RPL23AP68 |  |  | 39055 | NaN | 0.3264 | No |
| 1311 | ENSG00000242858 | AC020899.1 |  |  | 39057 | NaN | 0.3264 | No |
| 1312 | ENSG00000267585 | AC013290.1 |  |  | 39076 | NaN | 0.3261 | No |
| 1313 | ENSG00000258632 | AC135626.1 |  |  | 39077 | NaN | 0.3261 | No |
| 1314 | ENSG00000267589 | AC011444.4 |  |  | 39080 | NaN | 0.3260 | No |
| 1315 | ENSG00000237226 | RPS3AP39 |  |  | 39120 | NaN | 0.3254 | No |
| 1316 | ENSG00000227583 | RPS3AP37 |  |  | 39127 | NaN | 0.3253 | No |
| 1317 | ENSG00000236768 | AC124916.1 |  |  | 39136 | NaN | 0.3251 | No |
| 1318 | ENSG00000232925 | MRPS16P2 |  |  | 39160 | NaN | 0.3247 | No |
| 1319 | ENSG00000270458 | AC009779.6 |  |  | 39174 | NaN | 0.3245 | No |
| 1320 | ENSG00000214657 | AC113608.1 |  |  | 39184 | NaN | 0.3244 | No |
| 1321 | ENSG00000240954 | RPL4P1 |  |  | 39199 | NaN | 0.3241 | No |
| 1322 | ENSG00000217089 | RPS29P13 |  |  | 39237 | NaN | 0.3235 | No |
| 1323 | ENSG00000242735 | RPSAP26 |  |  | 39242 | NaN | 0.3234 | No |
| 1324 | ENSG00000219146 | RPS4XP8 |  |  | 39250 | NaN | 0.3233 | No |
| 1325 | ENSG00000213717 | AC004692.1 |  |  | 39296 | NaN | 0.3225 | No |
| 1326 | ENSG00000223402 | AC074121.1 |  |  | 39317 | NaN | 0.3222 | No |
| 1327 | ENSG00000261702 | AC022254.1 |  |  | 39323 | NaN | 0.3221 | No |
| 1328 | ENSG00000220154 | AL355796.1 |  |  | 39342 | NaN | 0.3218 | No |
| 1329 | ENSG00000239983 | AC016687.1 |  |  | 39351 | NaN | 0.3217 | No |
| 1330 | ENSG00000271587 | AC004805.3 |  |  | 39354 | NaN | 0.3216 | No |
| 1331 | ENSG00000214903 | RPS15AP3 |  |  | 39370 | NaN | 0.3214 | No |
| 1332 | ENSG00000225933 | AC133965.1 |  |  | 39376 | NaN | 0.3213 | No |
| 1333 | ENSG00000244390 | RPS6P22 |  |  | 39377 | NaN | 0.3213 | No |
| 1334 | ENSG00000244640 | AC096922.1 |  |  | 39392 | NaN | 0.3211 | No |
| 1335 | ENSG00000244641 | RPS26P43 |  |  | 39393 | NaN | 0.3211 | No |
| 1336 | ENSG00000228844 | AL358176.3 |  |  | 39416 | NaN | 0.3207 | No |
| 1337 | ENSG00000228848 | AC105402.2 |  |  | 39419 | NaN | 0.3207 | No |
| 1338 | ENSG00000231322 | RPL13AP17 |  |  | 39429 | NaN | 0.3205 | No |
| 1339 | ENSG00000243675 | AC080013.2 |  |  | 39468 | NaN | 0.3199 | No |
| 1340 | ENSG00000230580 | AC021016.1 |  |  | 39475 | NaN | 0.3198 | No |
| 1341 | ENSG00000243601 | RPL12P24 |  |  | 39477 | NaN | 0.3197 | No |
| 1342 | ENSG00000243609 | RPS2P44 |  |  | 39479 | NaN | 0.3197 | No |
| 1343 | ENSG00000237583 | AC093166.4 |  |  | 39492 | NaN | 0.3195 | No |
| 1344 | ENSG00000233778 | AC104339.1 |  |  | 39516 | NaN | 0.3191 | No |
| 1345 | ENSG00000249617 | AC106872.7 |  |  | 39562 | NaN | 0.3184 | No |
| 1346 | ENSG00000235467 | RPL10AP5 |  |  | 39572 | NaN | 0.3182 | No |
| 1347 | ENSG00000213935 | RPL22P16 |  |  | 39604 | NaN | 0.3177 | No |
| 1348 | ENSG00000242405 | AC007537.2 |  |  | 39626 | NaN | 0.3173 | No |
| 1349 | ENSG00000205644 | AC025186.1 |  |  | 39640 | NaN | 0.3171 | No |
| 1350 | ENSG00000234985 | AC074085.2 |  |  | 39678 | NaN | 0.3164 | No |
| 1351 | ENSG00000236395 | AC093106.2 |  |  | 39695 | NaN | 0.3162 | No |
| 1352 | ENSG00000261813 | AC015720.2 |  |  | 39715 | NaN | 0.3158 | No |
| 1353 | ENSG00000228568 | AC006461.1 |  |  | 39752 | NaN | 0.3152 | No |
| 1354 | ENSG00000239861 | AP001486.1 |  |  | 39759 | NaN | 0.3151 | No |
| 1355 | ENSG00000226093 | RPS28P8 |  |  | 39764 | NaN | 0.3151 | No |
| 1356 | ENSG00000232883 | AL451081.2 |  |  | 39789 | NaN | 0.3146 | No |
| 1357 | ENSG00000267558 | AC087685.1 |  |  | 39805 | NaN | 0.3144 | No |
| 1358 | ENSG00000255459 | AC105233.3 |  |  | 39815 | NaN | 0.3142 | No |
| 1359 | ENSG00000240459 | AC093663.1 |  |  | 39829 | NaN | 0.3140 | No |
| 1360 | ENSG00000237930 | AC007563.3 |  |  | 39851 | NaN | 0.3137 | No |
| 1361 | ENSG00000241411 | AC104619.1 |  |  | 39878 | NaN | 0.3132 | No |
| 1362 | ENSG00000270699 | AC012493.3 |  |  | 39922 | NaN | 0.3125 | No |
| 1363 | ENSG00000224497 | RPL36P4 |  |  | 39933 | NaN | 0.3123 | No |
| 1364 | ENSG00000237107 | AL591473.1 |  |  | 39945 | NaN | 0.3121 | No |
| 1365 | ENSG00000230169 | RPL30P16 |  |  | 39976 | NaN | 0.3116 | No |
| 1366 | ENSG00000258506 | AL163642.1 |  |  | 40010 | NaN | 0.3110 | No |
| 1367 | ENSG00000218300 | RPL22P14 |  |  | 40047 | NaN | 0.3104 | No |
| 1368 | ENSG00000228341 | AC002381.1 |  |  | 40054 | NaN | 0.3103 | No |
| 1369 | ENSG00000239210 | RPS26P55 |  |  | 40076 | NaN | 0.3099 | No |
| 1370 | ENSG00000213087 | AL356535.1 |  |  | 40077 | NaN | 0.3099 | No |
| 1371 | ENSG00000241250 | RPL17P20 |  |  | 40125 | NaN | 0.3091 | No |
| 1372 | ENSG00000184139 | RPL7AP28 |  |  | 40128 | NaN | 0.3091 | No |
| 1373 | ENSG00000244159 | RPS27AP13 |  |  | 40136 | NaN | 0.3090 | No |
| 1374 | ENSG00000227187 | AC007435.1 |  |  | 40191 | NaN | 0.3080 | No |
| 1375 | ENSG00000220370 | AL078595.1 |  |  | 40202 | NaN | 0.3079 | No |
| 1376 | ENSG00000239317 | AC091959.1 |  |  | 40210 | NaN | 0.3077 | No |
| 1377 | ENSG00000231747 | AC079922.1 |  |  | 40222 | NaN | 0.3076 | No |
| 1378 | ENSG00000237745 | RPL22P8 |  |  | 40237 | NaN | 0.3073 | No |
| 1379 | ENSG00000213361 | AC004884.1 |  |  | 40257 | NaN | 0.3070 | No |
| 1380 | ENSG00000254014 | AC009902.1 |  |  | 40261 | NaN | 0.3070 | No |
| 1381 | ENSG00000249692 | AC009567.2 |  |  | 40267 | NaN | 0.3069 | No |
| 1382 | ENSG00000224864 | AC011447.1 |  |  | 40314 | NaN | 0.3061 | No |
| 1383 | ENSG00000232625 | RPL23AP31 |  |  | 40331 | NaN | 0.3058 | No |
| 1384 | ENSG00000232622 | AC114482.1 |  |  | 40332 | NaN | 0.3058 | No |
| 1385 | ENSG00000224282 | AC010469.1 |  |  | 40345 | NaN | 0.3056 | No |
| 1386 | ENSG00000241809 | AC022133.1 |  |  | 40369 | NaN | 0.3052 | No |
| 1387 | ENSG00000229087 | AC007738.1 |  |  | 40383 | NaN | 0.3050 | No |
| 1388 | ENSG00000216854 | RPL17P26 |  |  | 40401 | NaN | 0.3047 | No |
| 1389 | ENSG00000213519 | AC132008.1 |  |  | 40420 | NaN | 0.3044 | No |
| 1390 | ENSG00000242060 | RPS3AP49 |  |  | 40446 | NaN | 0.3040 | No |
| 1391 | ENSG00000242061 | AL160314.1 |  |  | 40447 | NaN | 0.3040 | No |
| 1392 | ENSG00000242067 | RPL9P28 |  |  | 40452 | NaN | 0.3039 | No |
| 1393 | ENSG00000226948 | RPS4XP2 |  |  | 40483 | NaN | 0.3034 | No |
| 1394 | ENSG00000271609 | AC005632.3 |  |  | 40527 | NaN | 0.3026 | No |
| 1395 | ENSG00000244052 | RPL5P24 |  |  | 40535 | NaN | 0.3025 | No |
| 1396 | ENSG00000233990 | AL353754.1 |  |  | 40565 | NaN | 0.3020 | No |
| 1397 | ENSG00000232701 | RPL21P108 |  |  | 40591 | NaN | 0.3016 | No |
| 1398 | ENSG00000234624 | AC016894.1 |  |  | 40597 | NaN | 0.3015 | No |
| 1399 | ENSG00000243521 | RPL5P33 |  |  | 40626 | NaN | 0.3010 | No |
| 1400 | ENSG00000236004 | AL139156.3 |  |  | 40649 | NaN | 0.3006 | No |
| 1401 | ENSG00000214835 | RPL23AP6 |  |  | 40673 | NaN | 0.3002 | No |
| 1402 | ENSG00000216676 | AL031229.1 |  |  | 40694 | NaN | 0.2999 | No |
| 1403 | ENSG00000226242 | RPS17P8 |  |  | 40734 | NaN | 0.2992 | No |
| 1404 | ENSG00000244295 | RPS20P21 |  |  | 40738 | NaN | 0.2992 | No |
| 1405 | ENSG00000226545 | AL357552.1 |  |  | 40743 | NaN | 0.2991 | No |
| 1406 | ENSG00000284116 | AL772307.1 |  |  | 40808 | NaN | 0.2980 | No |
| 1407 | ENSG00000243720 | RPL21P127 |  |  | 40852 | NaN | 0.2972 | No |
| 1408 | ENSG00000243721 | RPL23AP63 |  |  | 40853 | NaN | 0.2973 | No |
| 1409 | ENSG00000243894 | AC116562.1 |  |  | 40869 | NaN | 0.2970 | No |
| 1410 | ENSG00000237314 | RPL12P39 |  |  | 40892 | NaN | 0.2966 | No |
| 1411 | ENSG00000259535 | RPL21P12 |  |  | 40894 | NaN | 0.2966 | No |
| 1412 | ENSG00000235264 | RPL5P28 |  |  | 40948 | NaN | 0.2957 | No |
| 1413 | ENSG00000215313 | RPL6P30 |  |  | 40963 | NaN | 0.2954 | No |
| 1414 | ENSG00000219928 | AL161787.1 |  |  | 41002 | NaN | 0.2948 | No |
| 1415 | ENSG00000234559 | RPL22P7 |  |  | 41005 | NaN | 0.2948 | No |
| 1416 | ENSG00000270701 | AP002802.2 |  |  | 41009 | NaN | 0.2947 | No |
| 1417 | ENSG00000259452 | AC009269.1 |  |  | 41024 | NaN | 0.2945 | No |
| 1418 | ENSG00000234907 | AL593851.1 |  |  | 41042 | NaN | 0.2942 | No |
| 1419 | ENSG00000227207 | RPL31P12 |  |  | 41106 | NaN | 0.2931 | No |
| 1420 | ENSG00000228190 | RPL23AP16 |  |  | 41108 | NaN | 0.2931 | No |
| 1421 | ENSG00000240611 | AC010307.1 |  |  | 41117 | NaN | 0.2929 | No |
| 1422 | ENSG00000216754 | AL050335.1 |  |  | 41133 | NaN | 0.2927 | No |
| 1423 | ENSG00000213820 | RPL13P2 |  |  | 41146 | NaN | 0.2925 | No |
| 1424 | ENSG00000232965 | RPS12P18 |  |  | 41183 | NaN | 0.2919 | No |
| 1425 | ENSG00000241511 | RPS15AP24 |  |  | 41280 | NaN | 0.2902 | No |
| 1426 | ENSG00000223777 | AC092162.1 |  |  | 41318 | NaN | 0.2895 | No |
| 1427 | ENSG00000224203 | RPS23P10 |  |  | 41363 | NaN | 0.2888 | No |
| 1428 | ENSG00000215088 | RPS5P3 |  |  | 41371 | NaN | 0.2886 | No |
| 1429 | ENSG00000234589 | AC125807.1 |  |  | 41390 | NaN | 0.2883 | No |
| 1430 | ENSG00000230809 | RPS6P15 |  |  | 41396 | NaN | 0.2883 | No |
| 1431 | ENSG00000226110 | BX323845.2 |  |  | 41414 | NaN | 0.2880 | No |
| 1432 | ENSG00000226117 | AC090844.1 |  |  | 41417 | NaN | 0.2879 | No |
| 1433 | ENSG00000235224 | AL591212.2 |  |  | 41473 | NaN | 0.2870 | No |
| 1434 | ENSG00000217030 | AL359643.1 |  |  | 41474 | NaN | 0.2870 | No |
| 1435 | ENSG00000216347 | RPL10P10 |  |  | 41476 | NaN | 0.2870 | No |
| 1436 | ENSG00000235226 | AL445528.1 |  |  | 41484 | NaN | 0.2869 | No |
| 1437 | ENSG00000236487 | AC079168.2 |  |  | 41502 | NaN | 0.2866 | No |
| 1438 | ENSG00000237729 | AC002075.2 |  |  | 41524 | NaN | 0.2862 | No |
| 1439 | ENSG00000241067 | RPL17P40 |  |  | 41538 | NaN | 0.2860 | No |
| 1440 | ENSG00000241069 | AC125388.1 |  |  | 41540 | NaN | 0.2860 | No |
| 1441 | ENSG00000227578 | RPS3AP53 |  |  | 41554 | NaN | 0.2858 | No |
| 1442 | ENSG00000241008 | AC044810.1 |  |  | 41574 | NaN | 0.2854 | No |
| 1443 | ENSG00000240270 | RPL12P37 |  |  | 41628 | NaN | 0.2845 | No |
| 1444 | ENSG00000235685 | AC092473.1 |  |  | 41630 | NaN | 0.2845 | No |
| 1445 | ENSG00000241891 | RPL9P6 |  |  | 41639 | NaN | 0.2844 | No |
| 1446 | ENSG00000250322 | AC026402.1 |  |  | 41641 | NaN | 0.2844 | No |
| 1447 | ENSG00000231660 | RPS8P6 |  |  | 41648 | NaN | 0.2843 | No |
| 1448 | ENSG00000230391 | RPSAP23 |  |  | 41654 | NaN | 0.2842 | No |
| 1449 | ENSG00000240074 | RPL9P30 |  |  | 41677 | NaN | 0.2838 | No |
| 1450 | ENSG00000244146 | AC106872.3 |  |  | 41724 | NaN | 0.2830 | No |
| 1451 | ENSG00000235579 | AC007283.2 |  |  | 41727 | NaN | 0.2830 | No |
| 1452 | ENSG00000235573 | AC005005.2 |  |  | 41730 | NaN | 0.2829 | No |
| 1453 | ENSG00000229168 | RPL19P20 |  |  | 41776 | NaN | 0.2822 | No |
| 1454 | ENSG00000214846 | AC114744.1 |  |  | 41799 | NaN | 0.2818 | No |
| 1455 | ENSG00000226327 | RPSAP49 |  |  | 41801 | NaN | 0.2818 | No |
| 1456 | ENSG00000213653 | RPL22P22 |  |  | 41812 | NaN | 0.2816 | No |
| 1457 | ENSG00000238263 | RPL21P88 |  |  | 41828 | NaN | 0.2814 | No |
| 1458 | ENSG00000223918 | AL513328.1 |  |  | 41830 | NaN | 0.2813 | No |
| 1459 | ENSG00000223916 | AC097638.1 |  |  | 41833 | NaN | 0.2813 | No |
| 1460 | ENSG00000229985 | AL590640.2 |  |  | 41853 | NaN | 0.2810 | No |
| 1461 | ENSG00000240424 | AC092824.1 |  |  | 41865 | NaN | 0.2808 | No |
| 1462 | ENSG00000237425 | RPSAP2 |  |  | 41882 | NaN | 0.2805 | No |
| 1463 | ENSG00000240271 | AC090142.1 |  |  | 41898 | NaN | 0.2803 | No |
| 1464 | ENSG00000227805 | RPL21P90 |  |  | 41924 | NaN | 0.2798 | No |
| 1465 | ENSG00000253262 | AC019176.1 |  |  | 41958 | NaN | 0.2793 | No |
| 1466 | ENSG00000226142 | RPL35P8 |  |  | 41965 | NaN | 0.2792 | No |
| 1467 | ENSG00000226144 | RPS27AP3 |  |  | 41968 | NaN | 0.2791 | No |
| 1468 | ENSG00000232858 | RPL34P27 |  |  | 41973 | NaN | 0.2791 | No |
| 1469 | ENSG00000241917 | AC084398.1 |  |  | 41984 | NaN | 0.2789 | No |
| 1470 | ENSG00000223640 | RPL30P3 |  |  | 42072 | NaN | 0.2774 | No |
| 1471 | ENSG00000239615 | AC104109.1 |  |  | 42082 | NaN | 0.2772 | No |
| 1472 | ENSG00000226428 | RPL15P13 |  |  | 42134 | NaN | 0.2763 | No |
| 1473 | ENSG00000227770 | RPL7P12 |  |  | 42137 | NaN | 0.2763 | No |
| 1474 | ENSG00000227777 | AL031736.1 |  |  | 42139 | NaN | 0.2763 | No |
| 1475 | ENSG00000225487 | AL390119.1 |  |  | 42154 | NaN | 0.2761 | No |
| 1476 | ENSG00000225483 | RPL22P4 |  |  | 42164 | NaN | 0.2759 | No |
| 1477 | ENSG00000225482 | RPS6P14 |  |  | 42166 | NaN | 0.2759 | No |
| 1478 | ENSG00000217181 | AL139039.2 |  |  | 42205 | NaN | 0.2753 | No |
| 1479 | ENSG00000224427 | AP000281.1 |  |  | 42206 | NaN | 0.2753 | No |
| 1480 | ENSG00000239281 | RPS29P2 |  |  | 42247 | NaN | 0.2746 | No |
| 1481 | ENSG00000237213 | RPL23AP22 |  |  | 42258 | NaN | 0.2744 | No |
| 1482 | ENSG00000243830 | AC092865.1 |  |  | 42270 | NaN | 0.2742 | No |
| 1483 | ENSG00000241590 | RPL17P37 |  |  | 42283 | NaN | 0.2740 | No |
| 1484 | ENSG00000233460 | RPL35AP31 |  |  | 42312 | NaN | 0.2735 | No |
| 1485 | ENSG00000234270 | RPL36P20 |  |  | 42344 | NaN | 0.2730 | No |
| 1486 | ENSG00000236312 | RPL34P34 |  |  | 42437 | NaN | 0.2714 | No |
| 1487 | ENSG00000213657 | RPL31P44 |  |  | 42438 | NaN | 0.2714 | No |
| 1488 | ENSG00000229865 | RPL31P54 |  |  | 42445 | NaN | 0.2713 | No |
| 1489 | novel.251 | - |  |  | 42485 | NaN | 0.2706 | No |
| 1490 | ENSG00000225381 | RPS5P7 |  |  | 42517 | NaN | 0.2701 | No |
| 1491 | ENSG00000257905 | AC004486.1 |  |  | 42548 | NaN | 0.2696 | No |
| 1492 | ENSG00000251062 | AC025459.1 |  |  | 42550 | NaN | 0.2695 | No |
| 1493 | ENSG00000229054 | RPS29P14 |  |  | 42557 | NaN | 0.2694 | No |
| 1494 | ENSG00000165496 | RPL10L |  |  | 42573 | NaN | 0.2692 | No |
| 1495 | ENSG00000242636 | RPL21P129 |  |  | 42577 | NaN | 0.2691 | No |
| 1496 | ENSG00000216938 | RPL7P58 |  |  | 42619 | NaN | 0.2684 | No |
| 1497 | ENSG00000237003 | AC126124.2 |  |  | 42693 | NaN | 0.2672 | No |
| 1498 | ENSG00000240385 | RPS29P20 |  |  | 42697 | NaN | 0.2671 | No |
| 1499 | ENSG00000281087 | AC135068.6 |  |  | 42702 | NaN | 0.2670 | No |
| 1500 | ENSG00000236667 | AC104076.2 |  |  | 42724 | NaN | 0.2667 | No |
| 1501 | ENSG00000227560 | RPS15AP30 |  |  | 42738 | NaN | 0.2665 | No |
| 1502 | ENSG00000224560 | AC108043.1 |  |  | 42749 | NaN | 0.2663 | No |
| 1503 | ENSG00000225352 | RPL31P53 |  |  | 42758 | NaN | 0.2662 | No |
| 1504 | ENSG00000213940 | AC026477.1 |  |  | 42791 | NaN | 0.2656 | No |
| 1505 | ENSG00000213495 | AC012498.1 |  |  | 42806 | NaN | 0.2654 | No |
| 1506 | ENSG00000242439 | AC127024.1 |  |  | 42821 | NaN | 0.2651 | No |
| 1507 | ENSG00000238069 | RPLP0P7 |  |  | 42901 | NaN | 0.2637 | No |
| 1508 | ENSG00000225024 | AC015977.1 |  |  | 42912 | NaN | 0.2636 | No |
| 1509 | ENSG00000227887 | RPS26P13 |  |  | 42916 | NaN | 0.2635 | No |
| 1510 | ENSG00000251313 | AC073648.4 |  |  | 42918 | NaN | 0.2635 | No |
| 1511 | ENSG00000241143 | RPL32P9 |  |  | 42933 | NaN | 0.2633 | No |
| 1512 | ENSG00000241146 | RPL7P41 |  |  | 42935 | NaN | 0.2633 | No |
| 1513 | ENSG00000227312 | AL445686.1 |  |  | 43001 | NaN | 0.2621 | No |
| 1514 | ENSG00000227311 | AL031985.2 |  |  | 43002 | NaN | 0.2622 | No |
| 1515 | ENSG00000233940 | RPL12P45 |  |  | 43009 | NaN | 0.2621 | No |
| 1516 | ENSG00000243171 | AC041039.1 |  |  | 43022 | NaN | 0.2619 | No |
| 1517 | ENSG00000241993 | RPL38P1 |  |  | 43026 | NaN | 0.2618 | No |
| 1518 | ENSG00000231508 | RPL34P20 |  |  | 43035 | NaN | 0.2617 | No |
| 1519 | ENSG00000240484 | AC091544.1 |  |  | 43046 | NaN | 0.2615 | No |
| 1520 | ENSG00000240486 | RPL23AP54 |  |  | 43047 | NaN | 0.2615 | No |
| 1521 | ENSG00000249851 | RPS27AP18 |  |  | 43062 | NaN | 0.2613 | No |
| 1522 | ENSG00000243822 | AC073522.1 |  |  | 43078 | NaN | 0.2610 | No |
| 1523 | ENSG00000249278 | AC112249.1 |  |  | 43092 | NaN | 0.2608 | No |
| 1524 | ENSG00000241462 | AC100832.1 |  |  | 43102 | NaN | 0.2607 | No |
| 1525 | ENSG00000231996 | AC096949.1 |  |  | 43149 | NaN | 0.2598 | No |
| 1526 | ENSG00000250752 | RPL23AP96 |  |  | 43152 | NaN | 0.2598 | No |
| 1527 | ENSG00000226205 | AC007790.1 |  |  | 43217 | NaN | 0.2587 | No |
| 1528 | ENSG00000216917 | AC005587.1 |  |  | 43227 | NaN | 0.2586 | No |
| 1529 | ENSG00000241088 | RPL17P44 |  |  | 43238 | NaN | 0.2584 | No |
| 1530 | ENSG00000242197 | AC098869.1 |  |  | 43272 | NaN | 0.2578 | No |
| 1531 | ENSG00000242198 | AC008897.1 |  |  | 43274 | NaN | 0.2578 | No |
| 1532 | ENSG00000256358 | RPL31P57 |  |  | 43275 | NaN | 0.2578 | No |
| 1533 | ENSG00000232083 | RPL31P7 |  |  | 43292 | NaN | 0.2575 | No |
| 1534 | ENSG00000224252 | AC113618.1 |  |  | 43294 | NaN | 0.2575 | No |
| 1535 | ENSG00000240131 | AL049829.1 |  |  | 43320 | NaN | 0.2571 | No |
| 1536 | ENSG00000262651 | AL513043.1 |  |  | 43352 | NaN | 0.2566 | No |
| 1537 | ENSG00000241846 | AC092451.1 |  |  | 43415 | NaN | 0.2555 | No |
| 1538 | ENSG00000243328 | AC083982.1 |  |  | 43447 | NaN | 0.2549 | No |
| 1539 | ENSG00000273876 | AC019176.2 |  |  | 43472 | NaN | 0.2545 | No |
| 1540 | ENSG00000240674 | AC106872.2 |  |  | 43478 | NaN | 0.2544 | No |
| 1541 | ENSG00000240673 | AC006539.1 |  |  | 43479 | NaN | 0.2545 | No |
| 1542 | ENSG00000259978 | MRPS21P8 |  |  | 43487 | NaN | 0.2543 | No |
| 1543 | ENSG00000275094 | AC079395.3 |  |  | 43498 | NaN | 0.2542 | No |
| 1544 | ENSG00000262759 | MRPS21P9 |  |  | 43503 | NaN | 0.2541 | No |
| 1545 | ENSG00000235425 | RPS7P13 |  |  | 43507 | NaN | 0.2541 | No |
| 1546 | ENSG00000238254 | AC008496.1 |  |  | 43532 | NaN | 0.2536 | No |
| 1547 | ENSG00000223450 | AL590632.1 |  |  | 43561 | NaN | 0.2532 | No |
| 1548 | ENSG00000223455 | AL355298.1 |  |  | 43565 | NaN | 0.2531 | No |
| 1549 | ENSG00000224980 | RPL23AP85 |  |  | 43602 | NaN | 0.2525 | No |
| 1550 | ENSG00000225475 | AC099789.1 |  |  | 43605 | NaN | 0.2525 | No |
| 1551 | ENSG00000229761 | RPS20P1 |  |  | 43613 | NaN | 0.2524 | No |
| 1552 | ENSG00000283043 | AC097655.1 |  |  | 43624 | NaN | 0.2522 | No |
| 1553 | ENSG00000244361 | RPL30P7 |  |  | 43625 | NaN | 0.2522 | No |
| 1554 | ENSG00000213527 | AC026826.1 |  |  | 43670 | NaN | 0.2514 | No |
| 1555 | ENSG00000213318 | AC009078.1 |  |  | 43706 | NaN | 0.2508 | No |
| 1556 | ENSG00000237088 | AL163193.1 |  |  | 43757 | NaN | 0.2499 | No |
| 1557 | ENSG00000242135 | RPL17P2 |  |  | 43761 | NaN | 0.2499 | No |
| 1558 | ENSG00000241656 | UBA52P7 |  |  | 43773 | NaN | 0.2497 | No |
| 1559 | ENSG00000241654 | RPL19P18 |  |  | 43775 | NaN | 0.2497 | No |
| 1560 | ENSG00000233702 | AC107083.1 |  |  | 43808 | NaN | 0.2492 | No |
| 1561 | ENSG00000254170 | AC008802.1 |  |  | 43828 | NaN | 0.2488 | No |
| 1562 | ENSG00000243514 | RPL32P33 |  |  | 43884 | NaN | 0.2479 | No |
| 1563 | ENSG00000248439 | RPL23AP94 |  |  | 43886 | NaN | 0.2479 | No |
| 1564 | ENSG00000242768 | AC096576.1 |  |  | 43901 | NaN | 0.2476 | No |
| 1565 | ENSG00000236580 | AC073626.2 |  |  | 43931 | NaN | 0.2471 | No |
| 1566 | ENSG00000217557 | AL590286.1 |  |  | 43934 | NaN | 0.2471 | No |
| 1567 | ENSG00000213641 | RPL7AP4 |  |  | 43939 | NaN | 0.2470 | No |
| 1568 | ENSG00000224600 | AL031864.2 |  |  | 43949 | NaN | 0.2469 | No |
| 1569 | ENSG00000224602 | RPS26P5 |  |  | 43950 | NaN | 0.2469 | No |
| 1570 | ENSG00000235581 | AC005011.1 |  |  | 43955 | NaN | 0.2468 | No |
| 1571 | ENSG00000235582 | AL365258.2 |  |  | 43957 | NaN | 0.2468 | No |
| 1572 | ENSG00000215905 | AL590609.1 |  |  | 43988 | NaN | 0.2463 | No |
| 1573 | ENSG00000228601 | RPL39P |  |  | 43993 | NaN | 0.2462 | No |
| 1574 | ENSG00000226515 | AC004386.2 |  |  | 44040 | NaN | 0.2454 | No |
| 1575 | ENSG00000227394 | AC007386.1 |  |  | 44056 | NaN | 0.2452 | No |
| 1576 | ENSG00000230180 | RPL12P49 |  |  | 44112 | NaN | 0.2442 | No |
| 1577 | ENSG00000230187 | AL139109.1 |  |  | 44114 | NaN | 0.2442 | No |
| 1578 | ENSG00000213880 | RPL7AP7 |  |  | 44116 | NaN | 0.2442 | No |
| 1579 | ENSG00000239525 | RPL30P5 |  |  | 44177 | NaN | 0.2431 | No |
| 1580 | ENSG00000270954 | RPSAP75 |  |  | 44188 | NaN | 0.2430 | No |
| 1581 | ENSG00000236748 | AC009500.1 |  |  | 44206 | NaN | 0.2427 | No |
| 1582 | ENSG00000224442 | AC017035.1 |  |  | 44219 | NaN | 0.2425 | No |
| 1583 | ENSG00000215297 | AL354941.1 |  |  | 44264 | NaN | 0.2417 | No |
| 1584 | ENSG00000214526 | AP000343.1 |  |  | 44271 | NaN | 0.2416 | No |
| 1585 | ENSG00000221879 | MRPS21P3 |  |  | 44302 | NaN | 0.2411 | No |
| 1586 | ENSG00000244267 | RPL34P22 |  |  | 44306 | NaN | 0.2411 | No |
| 1587 | ENSG00000237130 | MRPS6P2 |  |  | 44312 | NaN | 0.2410 | No |
| 1588 | novel.220 | - |  |  | 44316 | NaN | 0.2409 | No |
| 1589 | ENSG00000238149 | AC104978.1 |  |  | 44358 | NaN | 0.2402 | No |
| 1590 | ENSG00000225142 | AC114495.1 |  |  | 44366 | NaN | 0.2401 | No |
| 1591 | ENSG00000220643 | AL031577.2 |  |  | 44388 | NaN | 0.2397 | No |
| 1592 | ENSG00000226499 | AL136380.1 |  |  | 44405 | NaN | 0.2395 | No |
| 1593 | ENSG00000226498 | RPSAP21 |  |  | 44406 | NaN | 0.2395 | No |
| 1594 | ENSG00000226729 | RPL35AP30 |  |  | 44414 | NaN | 0.2394 | No |
| 1595 | ENSG00000253109 | RPL12P22 |  |  | 44430 | NaN | 0.2391 | No |
| 1596 | ENSG00000244708 | RPL23P10 |  |  | 44450 | NaN | 0.2388 | No |
| 1597 | ENSG00000244705 | AC020658.1 |  |  | 44452 | NaN | 0.2388 | No |
| 1598 | ENSG00000237824 | RPL23AP33 |  |  | 44495 | NaN | 0.2380 | No |
| 1599 | ENSG00000214727 | RPL5P35 |  |  | 44516 | NaN | 0.2377 | No |
| 1600 | ENSG00000237793 | RPL18AP16 |  |  | 44548 | NaN | 0.2372 | No |
| 1601 | ENSG00000239374 | AC090013.1 |  |  | 44571 | NaN | 0.2368 | No |
| 1602 | ENSG00000232540 | RPL36P19 |  |  | 44594 | NaN | 0.2364 | No |
| 1603 | ENSG00000257664 | RSL24D1P5 |  |  | 44607 | NaN | 0.2362 | No |
| 1604 | ENSG00000230853 | RPL10P11 |  |  | 44624 | NaN | 0.2359 | No |
| 1605 | ENSG00000239804 | AC092902.1 |  |  | 44630 | NaN | 0.2358 | No |
| 1606 | ENSG00000226128 | RPL26P9 |  |  | 44646 | NaN | 0.2356 | No |
| 1607 | ENSG00000213174 | AC026316.1 |  |  | 44658 | NaN | 0.2354 | No |
| 1608 | ENSG00000282834 | AP000787.3 |  |  | 44698 | NaN | 0.2347 | No |
| 1609 | ENSG00000217067 | AL355375.1 |  |  | 44712 | NaN | 0.2345 | No |
| 1610 | ENSG00000228210 | AL121916.1 |  |  | 44726 | NaN | 0.2343 | No |
| 1611 | ENSG00000218561 | AL353133.1 |  |  | 44730 | NaN | 0.2342 | No |
| 1612 | ENSG00000225338 | RPL23AP18 |  |  | 44775 | NaN | 0.2335 | No |
| 1613 | ENSG00000244002 | RPSAP39 |  |  | 44787 | NaN | 0.2333 | No |
| 1614 | ENSG00000244004 | AC097493.2 |  |  | 44789 | NaN | 0.2333 | No |
| 1615 | ENSG00000257404 | AC107016.2 |  |  | 44860 | NaN | 0.2320 | No |
| 1616 | ENSG00000262492 | AC104581.1 |  |  | 44866 | NaN | 0.2320 | No |
| 1617 | ENSG00000230691 | MRPL35P4 |  |  | 44890 | NaN | 0.2316 | No |
| 1618 | ENSG00000219547 | RPL17P25 |  |  | 44906 | NaN | 0.2313 | No |
| 1619 | ENSG00000243592 | RPL17P22 |  |  | 44910 | NaN | 0.2313 | No |
| 1620 | ENSG00000240027 | AC084357.1 |  |  | 44941 | NaN | 0.2308 | No |
| 1621 | ENSG00000241114 | AC008280.2 |  |  | 44952 | NaN | 0.2306 | No |
| 1622 | ENSG00000274154 | AC114781.5 |  |  | 44977 | NaN | 0.2302 | No |
| 1623 | ENSG00000224685 | AC099670.2 |  |  | 44998 | NaN | 0.2298 | No |
| 1624 | ENSG00000237478 | AL390023.1 |  |  | 45004 | NaN | 0.2297 | No |
| 1625 | ENSG00000234360 | AL135790.2 |  |  | 45009 | NaN | 0.2297 | No |
| 1626 | ENSG00000262120 | AC010547.6 |  |  | 45025 | NaN | 0.2294 | No |
| 1627 | ENSG00000227814 | MRPS17P9 |  |  | 45026 | NaN | 0.2294 | No |
| 1628 | ENSG00000244244 | RPS3AP42 |  |  | 45036 | NaN | 0.2293 | No |
| 1629 | ENSG00000225644 | RPL35AP4 |  |  | 45037 | NaN | 0.2293 | No |
| 1630 | ENSG00000213270 | RPL6P25 |  |  | 45054 | NaN | 0.2290 | No |
| 1631 | ENSG00000227645 | RPL7P54 |  |  | 45068 | NaN | 0.2288 | No |
| 1632 | ENSG00000179157 | RPS2P28 |  |  | 45077 | NaN | 0.2287 | No |
| 1633 | ENSG00000213448 | RPS23P2 |  |  | 45078 | NaN | 0.2287 | No |
| 1634 | ENSG00000242979 | RPS15AP18 |  |  | 45090 | NaN | 0.2285 | No |
| 1635 | ENSG00000250347 | AC005740.2 |  |  | 45107 | NaN | 0.2282 | No |
| 1636 | ENSG00000242353 | RPL12P30 |  |  | 45120 | NaN | 0.2280 | No |
| 1637 | ENSG00000229977 | AC073264.1 |  |  | 45130 | NaN | 0.2279 | No |
| 1638 | ENSG00000243758 | RPL35AP15 |  |  | 45141 | NaN | 0.2277 | No |
| 1639 | ENSG00000237450 | RPL23AP71 |  |  | 45152 | NaN | 0.2275 | No |
| 1640 | ENSG00000255106 | AC010768.3 |  |  | 45178 | NaN | 0.2271 | No |
| 1641 | ENSG00000229863 | AC097358.1 |  |  | 45196 | NaN | 0.2268 | No |
| 1642 | ENSG00000232867 | AL355433.1 |  |  | 45235 | NaN | 0.2261 | No |
| 1643 | ENSG00000243256 | RPL30P14 |  |  | 45245 | NaN | 0.2260 | No |
| 1644 | ENSG00000243250 | RPS6P16 |  |  | 45246 | NaN | 0.2260 | No |
| 1645 | ENSG00000237959 | RPL36AP10 |  |  | 45253 | NaN | 0.2259 | No |
| 1646 | ENSG00000233380 | RPS2P48 |  |  | 45278 | NaN | 0.2255 | No |
| 1647 | ENSG00000236159 | AL031659.1 |  |  | 45280 | NaN | 0.2255 | No |
| 1648 | ENSG00000271508 | AC090525.3 |  |  | 45304 | NaN | 0.2251 | No |
| 1649 | ENSG00000218476 | AL591416.1 |  |  | 45313 | NaN | 0.2250 | No |
| 1650 | ENSG00000216412 | RPL12P2 |  |  | 45331 | NaN | 0.2247 | No |
| 1651 | ENSG00000239719 | AC004890.1 |  |  | 45376 | NaN | 0.2239 | No |
| 1652 | ENSG00000226410 | AC104058.1 |  |  | 45398 | NaN | 0.2235 | No |
| 1653 | ENSG00000213896 | AC109486.1 |  |  | 45399 | NaN | 0.2235 | No |
| 1654 | ENSG00000242834 | RPL7AP56 |  |  | 45413 | NaN | 0.2233 | No |
| 1655 | ENSG00000226233 | Z82188.1 |  |  | 45444 | NaN | 0.2228 | No |
| 1656 | ENSG00000257389 | AC107016.1 |  |  | 45483 | NaN | 0.2221 | No |
| 1657 | ENSG00000217404 | RPS4XP9 |  |  | 45492 | NaN | 0.2220 | No |
| 1658 | ENSG00000253600 | AC008446.1 |  |  | 45496 | NaN | 0.2220 | No |
| 1659 | ENSG00000230734 | RPL10P3 |  |  | 45506 | NaN | 0.2218 | No |
| 1660 | ENSG00000243494 | AC011476.1 |  |  | 45510 | NaN | 0.2218 | No |
| 1661 | ENSG00000237170 | RPS7P15 |  |  | 45551 | NaN | 0.2211 | No |
| 1662 | ENSG00000186244 | AC091180.1 |  |  | 45593 | NaN | 0.2204 | No |
| 1663 | ENSG00000224279 | RPS29P31 |  |  | 45605 | NaN | 0.2202 | No |
| 1664 | ENSG00000214641 | AL445529.1 |  |  | 45622 | NaN | 0.2199 | No |
| 1665 | ENSG00000241888 | RPSAP37 |  |  | 45658 | NaN | 0.2193 | No |
| 1666 | ENSG00000212695 | AL583805.1 |  |  | 45686 | NaN | 0.2188 | No |
| 1667 | ENSG00000228292 | AL512604.1 |  |  | 45699 | NaN | 0.2186 | No |
| 1668 | ENSG00000260817 | AC074051.3 |  |  | 45735 | NaN | 0.2180 | No |
| 1669 | ENSG00000234735 | AL022237.1 |  |  | 45768 | NaN | 0.2175 | No |
| 1670 | ENSG00000239694 | RPSAP38 |  |  | 45775 | NaN | 0.2174 | No |
| 1671 | ENSG00000244674 | RPS3AP15 |  |  | 45782 | NaN | 0.2173 | No |
| 1672 | ENSG00000244088 | RPL23AP44 |  |  | 45783 | NaN | 0.2173 | No |
| 1673 | ENSG00000244083 | AC021205.2 |  |  | 45788 | NaN | 0.2172 | No |
| 1674 | ENSG00000229067 | AL714022.1 |  |  | 45806 | NaN | 0.2169 | No |
| 1675 | ENSG00000257480 | MRPL2P1 |  |  | 45818 | NaN | 0.2167 | No |
| 1676 | ENSG00000242667 | AC087385.2 |  |  | 45828 | NaN | 0.2166 | No |
| 1677 | ENSG00000242634 | RPS24P16 |  |  | 45837 | NaN | 0.2165 | No |
| 1678 | ENSG00000241052 | RPL23AP70 |  |  | 45857 | NaN | 0.2161 | No |
| 1679 | ENSG00000240531 | RPL21P123 |  |  | 45884 | NaN | 0.2157 | No |
| 1680 | ENSG00000236509 | RPL21P133 |  |  | 45894 | NaN | 0.2155 | No |
| 1681 | ENSG00000178715 | AL450998.1 |  |  | 45915 | NaN | 0.2152 | No |
| 1682 | ENSG00000271153 | RPL23AP88 |  |  | 45950 | NaN | 0.2146 | No |
| 1683 | ENSG00000271150 | AC008745.1 |  |  | 45951 | NaN | 0.2146 | No |
| 1684 | ENSG00000236783 | RPS15AP27 |  |  | 45974 | NaN | 0.2142 | No |
| 1685 | ENSG00000242986 | RPL21P99 |  |  | 46003 | NaN | 0.2137 | No |
| 1686 | ENSG00000237162 | AC007098.1 |  |  | 46027 | NaN | 0.2133 | No |
| 1687 | ENSG00000216902 | AL122016.1 |  |  | 46033 | NaN | 0.2133 | No |
| 1688 | ENSG00000219253 | RPS6P7 |  |  | 46036 | NaN | 0.2132 | No |
| 1689 | ENSG00000242461 | RPS15AP32 |  |  | 46071 | NaN | 0.2126 | No |
| 1690 | ENSG00000235787 | AC092047.1 |  |  | 46073 | NaN | 0.2126 | No |
| 1691 | ENSG00000232172 | AL359092.2 |  |  | 46082 | NaN | 0.2125 | No |
| 1692 | ENSG00000214326 | RPL31P1 |  |  | 46083 | NaN | 0.2125 | No |
| 1693 | ENSG00000240821 | RPL9P25 |  |  | 46153 | NaN | 0.2113 | No |
| 1694 | ENSG00000227854 | AL590135.1 |  |  | 46165 | NaN | 0.2111 | No |
| 1695 | ENSG00000187504 | RPL7P48 |  |  | 46221 | NaN | 0.2102 | No |
| 1696 | ENSG00000231556 | RSL24D1P3 |  |  | 46282 | NaN | 0.2091 | No |
| 1697 | ENSG00000231559 | Z95118.1 |  |  | 46286 | NaN | 0.2091 | No |
| 1698 | ENSG00000203648 | AC007618.1 |  |  | 46324 | NaN | 0.2084 | No |
| 1699 | ENSG00000241385 | AC023245.1 |  |  | 46448 | NaN | 0.2063 | No |
| 1700 | ENSG00000235644 | RPL10P5 |  |  | 46452 | NaN | 0.2062 | No |
| 1701 | ENSG00000257074 | RPL29P33 |  |  | 46494 | NaN | 0.2055 | No |
| 1702 | ENSG00000242169 | AC093789.1 |  |  | 46520 | NaN | 0.2051 | No |
| 1703 | ENSG00000239226 | MRPS17P3 |  |  | 46558 | NaN | 0.2044 | No |
| 1704 | ENSG00000225246 | RPS2P1 |  |  | 46593 | NaN | 0.2038 | No |
| 1705 | ENSG00000182888 | AC007685.1 |  |  | 46605 | NaN | 0.2037 | No |
| 1706 | ENSG00000240043 | RPS27P26 |  |  | 46663 | NaN | 0.2027 | No |
| 1707 | ENSG00000249987 | RPS4XP20 |  |  | 46714 | NaN | 0.2018 | No |
| 1708 | ENSG00000258013 | RPL3P13 |  |  | 46730 | NaN | 0.2015 | No |
| 1709 | ENSG00000261077 | AC016180.1 |  |  | 46738 | NaN | 0.2014 | No |
| 1710 | ENSG00000235619 | RPL36AP33 |  |  | 46762 | NaN | 0.2010 | No |
| 1711 | ENSG00000244131 | RPSAP51 |  |  | 46779 | NaN | 0.2007 | No |
| 1712 | ENSG00000228040 | AL390729.2 |  |  | 46794 | NaN | 0.2005 | No |
| 1713 | ENSG00000229795 | RPS21P1 |  |  | 46819 | NaN | 0.2001 | No |
| 1714 | ENSG00000214925 | AC234778.1 |  |  | 46828 | NaN | 0.2000 | No |
| 1715 | ENSG00000254200 | RPL7AP33 |  |  | 46842 | NaN | 0.1997 | No |
| 1716 | ENSG00000240534 | RPL34P17 |  |  | 46860 | NaN | 0.1994 | No |
| 1717 | ENSG00000224085 | RPL23AP41 |  |  | 46924 | NaN | 0.1983 | No |
| 1718 | ENSG00000241668 | RPL19P11 |  |  | 46974 | NaN | 0.1975 | No |
| 1719 | ENSG00000233558 | AL050331.1 |  |  | 46996 | NaN | 0.1971 | No |
| 1720 | ENSG00000235369 | RPL36AP15 |  |  | 47028 | NaN | 0.1966 | No |
| 1721 | ENSG00000231063 | AC006994.1 |  |  | 47049 | NaN | 0.1962 | No |
| 1722 | ENSG00000240174 | AP000852.1 |  |  | 47054 | NaN | 0.1962 | No |
| 1723 | ENSG00000213060 | AL031864.1 |  |  | 47060 | NaN | 0.1961 | No |
| 1724 | ENSG00000229554 | RPL21P24 |  |  | 47063 | NaN | 0.1961 | No |
| 1725 | ENSG00000213916 | RPL13P |  |  | 47072 | NaN | 0.1959 | No |
| 1726 | ENSG00000213917 | RPL5P8 |  |  | 47073 | NaN | 0.1960 | No |
| 1727 | ENSG00000213068 | AL451050.1 |  |  | 47110 | NaN | 0.1953 | No |
| 1728 | ENSG00000236571 | AC245102.2 |  |  | 47142 | NaN | 0.1948 | No |
| 1729 | ENSG00000235002 | AL513331.1 |  |  | 47175 | NaN | 0.1942 | No |
| 1730 | ENSG00000230469 | RPL5P26 |  |  | 47213 | NaN | 0.1936 | No |
| 1731 | ENSG00000242163 | AL121769.1 |  |  | 47227 | NaN | 0.1934 | No |
| 1732 | ENSG00000239809 | AC008026.2 |  |  | 47229 | NaN | 0.1934 | No |
| 1733 | ENSG00000229182 | MRPS16P1 |  |  | 47244 | NaN | 0.1931 | No |
| 1734 | ENSG00000227694 | RPL23AP74 |  |  | 47252 | NaN | 0.1930 | No |
| 1735 | ENSG00000244540 | AC087884.1 |  |  | 47258 | NaN | 0.1929 | No |
| 1736 | ENSG00000227343 | RPL15P1 |  |  | 47271 | NaN | 0.1927 | No |
| 1737 | ENSG00000213180 | RPL36AP48 |  |  | 47297 | NaN | 0.1923 | No |
| 1738 | ENSG00000213183 | RPS7P8 |  |  | 47299 | NaN | 0.1923 | No |
| 1739 | ENSG00000242431 | AC107398.1 |  |  | 47311 | NaN | 0.1921 | No |
| 1740 | ENSG00000227056 | RPL6P2 |  |  | 47320 | NaN | 0.1920 | No |
| 1741 | ENSG00000225661 | RPL14P5 |  |  | 47337 | NaN | 0.1917 | No |
| 1742 | ENSG00000235656 | RPL36P18 |  |  | 47345 | NaN | 0.1916 | No |
| 1743 | ENSG00000237463 | AL157714.2 |  |  | 47351 | NaN | 0.1915 | No |
| 1744 | ENSG00000230603 | AC011753.1 |  |  | 47358 | NaN | 0.1914 | No |
| 1745 | ENSG00000243780 | AC092754.2 |  |  | 47362 | NaN | 0.1914 | No |
| 1746 | ENSG00000237952 | RPL7AP73 |  |  | 47366 | NaN | 0.1913 | No |
| 1747 | ENSG00000237953 | AC013267.1 |  |  | 47367 | NaN | 0.1913 | No |
| 1748 | ENSG00000241782 | AP002812.1 |  |  | 47372 | NaN | 0.1913 | No |
| 1749 | ENSG00000241434 | AC022137.1 |  |  | 47388 | NaN | 0.1910 | No |
| 1750 | ENSG00000172746 | RPL12P13 |  |  | 47417 | NaN | 0.1905 | No |
| 1751 | ENSG00000236777 | AL157831.1 |  |  | 47421 | NaN | 0.1905 | No |
| 1752 | ENSG00000236776 | RPL21P23 |  |  | 47422 | NaN | 0.1905 | No |
| 1753 | ENSG00000244593 | AC025062.1 |  |  | 47432 | NaN | 0.1903 | No |
| 1754 | ENSG00000224475 | AC073900.1 |  |  | 47436 | NaN | 0.1903 | No |
| 1755 | ENSG00000264054 | RPL31P9 |  |  | 47441 | NaN | 0.1902 | No |
| 1756 | ENSG00000237549 | AC092570.3 |  |  | 47479 | NaN | 0.1896 | No |
| 1757 | ENSG00000234192 | RPS26P38 |  |  | 47486 | NaN | 0.1895 | No |
| 1758 | ENSG00000224907 | RPL21P91 |  |  | 47507 | NaN | 0.1891 | No |
| 1759 | ENSG00000232378 | RPL29P28 |  |  | 47540 | NaN | 0.1886 | No |
| 1760 | ENSG00000260033 | AC023813.2 |  |  | 47546 | NaN | 0.1885 | No |
| 1761 | ENSG00000260031 | RPL10P14 |  |  | 47547 | NaN | 0.1885 | No |
| 1762 | ENSG00000239223 | RPL34P31 |  |  | 47558 | NaN | 0.1883 | No |
| 1763 | ENSG00000238173 | RPL39P6 |  |  | 47589 | NaN | 0.1878 | No |
| 1764 | ENSG00000238172 | RPS2P35 |  |  | 47590 | NaN | 0.1878 | No |
| 1765 | ENSG00000227952 | RPL18AP17 |  |  | 47605 | NaN | 0.1876 | No |
| 1766 | ENSG00000240622 | RPL7P15 |  |  | 47626 | NaN | 0.1873 | No |
| 1767 | ENSG00000228429 | AC004910.1 |  |  | 47627 | NaN | 0.1873 | No |
| 1768 | ENSG00000214759 | AL355076.1 |  |  | 47641 | NaN | 0.1870 | No |
| 1769 | ENSG00000256664 | AC025423.2 |  |  | 47716 | NaN | 0.1857 | No |
| 1770 | ENSG00000215002 | RPL23AP59 |  |  | 47717 | NaN | 0.1858 | No |
| 1771 | ENSG00000215003 | RPL15P20 |  |  | 47718 | NaN | 0.1858 | No |
| 1772 | ENSG00000231767 | AL136454.1 |  |  | 47728 | NaN | 0.1856 | No |
| 1773 | ENSG00000240624 | AL138976.1 |  |  | 47739 | NaN | 0.1854 | No |
| 1774 | ENSG00000233268 | AL691449.1 |  |  | 47767 | NaN | 0.1850 | No |
| 1775 | ENSG00000235175 | RPL26P37 |  |  | 47768 | NaN | 0.1850 | No |
| 1776 | ENSG00000233260 | RPSAP63 |  |  | 47773 | NaN | 0.1849 | No |
| 1777 | ENSG00000236929 | AC112498.1 |  |  | 47808 | NaN | 0.1843 | No |
| 1778 | ENSG00000233335 | AC016696.1 |  |  | 47833 | NaN | 0.1839 | No |
| 1779 | ENSG00000267301 | RPL23AP77 |  |  | 47834 | NaN | 0.1839 | No |
| 1780 | ENSG00000229716 | RPL23AP19 |  |  | 47853 | NaN | 0.1836 | No |
| 1781 | ENSG00000244331 | AC008677.2 |  |  | 47867 | NaN | 0.1834 | No |
| 1782 | ENSG00000243516 | RPL12P40 |  |  | 47912 | NaN | 0.1826 | No |
| 1783 | ENSG00000242041 | RPL35AP28 |  |  | 47947 | NaN | 0.1820 | No |
| 1784 | ENSG00000228247 | UBBP2 |  |  | 47969 | NaN | 0.1817 | No |
| 1785 | ENSG00000239413 | RPS27P23 |  |  | 47982 | NaN | 0.1815 | No |
| 1786 | ENSG00000228681 | AC008072.1 |  |  | 47984 | NaN | 0.1815 | No |
| 1787 | ENSG00000267449 | AC005884.2 |  |  | 48009 | NaN | 0.1811 | No |
| 1788 | ENSG00000237432 | RPS7P12 |  |  | 48027 | NaN | 0.1808 | No |
| 1789 | ENSG00000241002 | AC017007.3 |  |  | 48031 | NaN | 0.1807 | No |
| 1790 | ENSG00000244071 | RPL9P33 |  |  | 48033 | NaN | 0.1807 | No |
| 1791 | ENSG00000268483 | RPL7AP69 |  |  | 48034 | NaN | 0.1807 | No |
| 1792 | ENSG00000270289 | AC012669.1 |  |  | 48072 | NaN | 0.1801 | No |
| 1793 | ENSG00000230837 | RPL31P2 |  |  | 48095 | NaN | 0.1797 | No |
| 1794 | ENSG00000230331 | AL160004.2 |  |  | 48110 | NaN | 0.1795 | No |
| 1795 | ENSG00000243095 | RPL3P10 |  |  | 48112 | NaN | 0.1794 | No |
| 1796 | ENSG00000243099 | AC020983.1 |  |  | 48114 | NaN | 0.1794 | No |
| 1797 | ENSG00000235869 | Z99127.2 |  |  | 48132 | NaN | 0.1791 | No |
| 1798 | ENSG00000213661 | AC106806.1 |  |  | 48159 | NaN | 0.1787 | No |
| 1799 | ENSG00000265402 | RSL24D1P9 |  |  | 48198 | NaN | 0.1780 | No |
| 1800 | ENSG00000235089 | AL357632.1 |  |  | 48205 | NaN | 0.1779 | No |
| 1801 | ENSG00000234683 | AL138799.3 |  |  | 48225 | NaN | 0.1776 | No |
| 1802 | ENSG00000230171 | RPL22P18 |  |  | 48232 | NaN | 0.1775 | No |
| 1803 | ENSG00000234352 | AC009264.1 |  |  | 48234 | NaN | 0.1775 | No |
| 1804 | ENSG00000234358 | AC003080.1 |  |  | 48237 | NaN | 0.1775 | No |
| 1805 | ENSG00000213729 | AC098828.1 |  |  | 48276 | NaN | 0.1768 | No |
| 1806 | ENSG00000225404 | RPL26P34 |  |  | 48297 | NaN | 0.1765 | No |
| 1807 | ENSG00000242941 | RPL12P7 |  |  | 48327 | NaN | 0.1760 | No |
| 1808 | ENSG00000242945 | RPL29P32 |  |  | 48329 | NaN | 0.1760 | No |
| 1809 | ENSG00000241103 | AC109925.1 |  |  | 48380 | NaN | 0.1751 | No |
| 1810 | ENSG00000227791 | AC007365.2 |  |  | 48382 | NaN | 0.1751 | No |
| 1811 | ENSG00000237483 | RPS27AP8 |  |  | 48402 | NaN | 0.1748 | No |
| 1812 | ENSG00000233562 | AL356981.1 |  |  | 48466 | NaN | 0.1737 | No |
| 1813 | ENSG00000241020 | AP000873.1 |  |  | 48484 | NaN | 0.1734 | No |
| 1814 | ENSG00000283095 | FP565171.1 |  |  | 48524 | NaN | 0.1727 | No |
| 1815 | ENSG00000261914 | RPL23AP84 |  |  | 48531 | NaN | 0.1726 | No |
| 1816 | ENSG00000234573 | AL121890.1 |  |  | 48536 | NaN | 0.1725 | No |
| 1817 | ENSG00000219361 | RPSAP72 |  |  | 48541 | NaN | 0.1725 | No |
| 1818 | ENSG00000236189 | RPL18AP15 |  |  | 48558 | NaN | 0.1722 | No |
| 1819 | ENSG00000213269 | AC004386.1 |  |  | 48560 | NaN | 0.1722 | No |
| 1820 | ENSG00000236238 | AC093106.1 |  |  | 48562 | NaN | 0.1722 | No |
| 1821 | ENSG00000240480 | RPL29P2 |  |  | 48584 | NaN | 0.1718 | No |
| 1822 | ENSG00000239627 | RPL12P20 |  |  | 48602 | NaN | 0.1715 | No |
| 1823 | ENSG00000239622 | AC005165.2 |  |  | 48607 | NaN | 0.1715 | No |
| 1824 | ENSG00000260965 | RPL23AP91 |  |  | 48614 | NaN | 0.1714 | No |
| 1825 | ENSG00000226448 | RPL7AP51 |  |  | 48632 | NaN | 0.1711 | No |
| 1826 | ENSG00000243287 | RPS17P14 |  |  | 48642 | NaN | 0.1709 | No |
| 1827 | ENSG00000213791 | AC104212.1 |  |  | 48657 | NaN | 0.1707 | No |
| 1828 | ENSG00000253155 | AC008676.3 |  |  | 48668 | NaN | 0.1705 | No |
| 1829 | ENSG00000213849 | AC104306.1 |  |  | 48672 | NaN | 0.1705 | No |
| 1830 | ENSG00000213579 | AL096829.1 |  |  | 48683 | NaN | 0.1703 | No |
| 1831 | ENSG00000227425 | MRPS21P2 |  |  | 48687 | NaN | 0.1703 | No |
| 1832 | ENSG00000242757 | AL606462.1 |  |  | 48711 | NaN | 0.1699 | No |
| 1833 | ENSG00000233707 | RPL22P11 |  |  | 48728 | NaN | 0.1696 | No |
| 1834 | ENSG00000229014 | RPL30P13 |  |  | 48797 | NaN | 0.1684 | No |
| 1835 | ENSG00000236433 | RPL37P21 |  |  | 48803 | NaN | 0.1683 | No |
| 1836 | ENSG00000254336 | AC008694.2 |  |  | 48824 | NaN | 0.1680 | No |
| 1837 | ENSG00000240179 | RPL26P3 |  |  | 48827 | NaN | 0.1679 | No |
| 1838 | ENSG00000128626 | MRPS12 |  |  | 48830 | NaN | 0.1679 | No |
| 1839 | ENSG00000271248 | AC091193.1 |  |  | 48831 | NaN | 0.1679 | No |
| 1840 | ENSG00000235163 | AC096644.3 |  |  | 48845 | NaN | 0.1677 | No |
| 1841 | ENSG00000232393 | RPL5P6 |  |  | 48847 | NaN | 0.1677 | No |
| 1842 | ENSG00000231965 | AF131215.1 |  |  | 48858 | NaN | 0.1675 | No |
| 1843 | ENSG00000230829 | AC025181.1 |  |  | 48877 | NaN | 0.1672 | No |
| 1844 | ENSG00000219758 | FO393415.2 |  |  | 48895 | NaN | 0.1669 | No |
| 1845 | ENSG00000265936 | AC091042.1 |  |  | 48922 | NaN | 0.1665 | No |
| 1846 | ENSG00000260379 | AC074052.1 |  |  | 48926 | NaN | 0.1664 | No |
| 1847 | ENSG00000239490 | RPS4XP18 |  |  | 48947 | NaN | 0.1661 | No |
| 1848 | ENSG00000213783 | RPL35P4 |  |  | 48954 | NaN | 0.1660 | No |
| 1849 | ENSG00000213787 | RPL7AP38 |  |  | 48957 | NaN | 0.1660 | No |
| 1850 | ENSG00000216365 | RPL37P15 |  |  | 48960 | NaN | 0.1659 | No |
| 1851 | ENSG00000226229 | RPLP0P1 |  |  | 48973 | NaN | 0.1657 | No |
| 1852 | ENSG00000229822 | AL353691.1 |  |  | 48985 | NaN | 0.1655 | No |
| 1853 | ENSG00000239412 | RPL21P71 |  |  | 48986 | NaN | 0.1656 | No |
| 1854 | ENSG00000226928 | RPS14P4 |  |  | 49010 | NaN | 0.1652 | No |
| 1855 | ENSG00000242963 | AC026336.1 |  |  | 49011 | NaN | 0.1652 | No |
| 1856 | ENSG00000228744 | RPS3AP30 |  |  | 49017 | NaN | 0.1651 | No |
| 1857 | ENSG00000183239 | AL109615.1 |  |  | 49025 | NaN | 0.1650 | No |
| 1858 | ENSG00000233934 | RPL21P38 |  |  | 49032 | NaN | 0.1649 | No |
| 1859 | ENSG00000233939 | AC114755.4 |  |  | 49033 | NaN | 0.1649 | No |
| 1860 | ENSG00000242654 | RPL32P14 |  |  | 49070 | NaN | 0.1643 | No |
| 1861 | ENSG00000231022 | RPS3AP9 |  |  | 49074 | NaN | 0.1642 | No |
| 1862 | ENSG00000256708 | AC007458.1 |  |  | 49089 | NaN | 0.1640 | No |
| 1863 | ENSG00000234995 | AC013429.2 |  |  | 49131 | NaN | 0.1633 | No |
| 1864 | ENSG00000240096 | AL132838.1 |  |  | 49138 | NaN | 0.1632 | No |
| 1865 | ENSG00000214142 | RPL7P60 |  |  | 49141 | NaN | 0.1631 | No |
| 1866 | ENSG00000260483 | AC015720.1 |  |  | 49290 | NaN | 0.1605 | No |
| 1867 | ENSG00000244076 | AC026410.2 |  |  | 49317 | NaN | 0.1601 | No |
| 1868 | ENSG00000235735 | RPL7AP72 |  |  | 49340 | NaN | 0.1597 | No |
| 1869 | ENSG00000239354 | RPS17P15 |  |  | 49344 | NaN | 0.1597 | No |
| 1870 | ENSG00000237409 | AL513302.1 |  |  | 49352 | NaN | 0.1596 | No |
| 1871 | ENSG00000241129 | RPL22P19 |  |  | 49386 | NaN | 0.1590 | No |
| 1872 | ENSG00000253383 | AC100784.1 |  |  | 49415 | NaN | 0.1585 | No |
| 1873 | ENSG00000244470 | AC105918.1 |  |  | 49416 | NaN | 0.1585 | No |
| 1874 | ENSG00000233829 | AC017078.1 |  |  | 49434 | NaN | 0.1582 | No |
| 1875 | ENSG00000270702 | AC073657.2 |  |  | 49456 | NaN | 0.1578 | No |
| 1876 | ENSG00000237041 | AC007679.2 |  |  | 49463 | NaN | 0.1578 | No |
| 1877 | ENSG00000279945 | AC013489.3 |  |  | 49475 | NaN | 0.1576 | No |
| 1878 | ENSG00000240759 | RPS27P21 |  |  | 49489 | NaN | 0.1573 | No |
| 1879 | ENSG00000233741 | AC016691.1 |  |  | 49509 | NaN | 0.1570 | No |
| 1880 | ENSG00000218459 | RPS27P15 |  |  | 49535 | NaN | 0.1566 | No |
| 1881 | ENSG00000223624 | AL033528.1 |  |  | 49549 | NaN | 0.1564 | No |
| 1882 | ENSG00000225713 | RPL30P1 |  |  | 49598 | NaN | 0.1555 | No |
| 1883 | ENSG00000244427 | AC105233.1 |  |  | 49616 | NaN | 0.1552 | No |
| 1884 | ENSG00000215800 | RSL24D1P4 |  |  | 49619 | NaN | 0.1552 | No |
| 1885 | ENSG00000232383 | AC010677.1 |  |  | 49644 | NaN | 0.1548 | No |
| 1886 | ENSG00000282994 | AC215524.1 |  |  | 49657 | NaN | 0.1546 | No |
| 1887 | ENSG00000242285 | RPL6P8 |  |  | 49683 | NaN | 0.1542 | No |
| 1888 | ENSG00000242137 | RPL11P5 |  |  | 49700 | NaN | 0.1539 | No |
| 1889 | ENSG00000243423 | AC004223.1 |  |  | 49705 | NaN | 0.1538 | No |
| 1890 | ENSG00000244048 | RPS18P6 |  |  | 49731 | NaN | 0.1534 | No |
| 1891 | ENSG00000244413 | RPL23AP56 |  |  | 49757 | NaN | 0.1530 | No |
| 1892 | ENSG00000233445 | RPL17P11 |  |  | 49810 | NaN | 0.1521 | No |
| 1893 | ENSG00000242814 | AC113398.1 |  |  | 49830 | NaN | 0.1517 | No |
| 1894 | ENSG00000234259 | AC008550.1 |  |  | 49838 | NaN | 0.1516 | No |
| 1895 | ENSG00000234253 | RPL7P13 |  |  | 49841 | NaN | 0.1516 | No |
| 1896 | ENSG00000230203 | Z99714.1 |  |  | 49883 | NaN | 0.1509 | No |
| 1897 | ENSG00000272407 | AL022394.1 |  |  | 49892 | NaN | 0.1507 | No |
| 1898 | ENSG00000219135 | RPL23AP48 |  |  | 49901 | NaN | 0.1506 | No |
| 1899 | ENSG00000224291 | AC012671.1 |  |  | 49903 | NaN | 0.1506 | No |
| 1900 | ENSG00000223628 | AC023449.1 |  |  | 49907 | NaN | 0.1506 | No |
| 1901 | ENSG00000249600 | AC106806.2 |  |  | 49930 | NaN | 0.1502 | No |
| 1902 | ENSG00000177776 | RPS2P2 |  |  | 49982 | NaN | 0.1493 | No |
| 1903 | ENSG00000262990 | AC004706.2 |  |  | 49991 | NaN | 0.1492 | No |
| 1904 | ENSG00000225907 | RPS27P16 |  |  | 50050 | NaN | 0.1481 | No |
| 1905 | ENSG00000254252 | RPL7P20 |  |  | 50070 | NaN | 0.1478 | No |
| 1906 | ENSG00000213309 | RPL9P18 |  |  | 50134 | NaN | 0.1467 | No |
| 1907 | ENSG00000224593 | AC092427.1 |  |  | 50138 | NaN | 0.1467 | No |
| 1908 | ENSG00000230595 | RSL24D1P2 |  |  | 50154 | NaN | 0.1464 | No |
| 1909 | ENSG00000213900 | RPS17P1 |  |  | 50162 | NaN | 0.1463 | No |
| 1910 | ENSG00000182383 | RPL27AP5 |  |  | 50170 | NaN | 0.1462 | No |
| 1911 | ENSG00000218716 | RPL12P23 |  |  | 50224 | NaN | 0.1453 | No |
| 1912 | ENSG00000241261 | RPL17P19 |  |  | 50260 | NaN | 0.1446 | No |
| 1913 | ENSG00000225005 | AC211485.1 |  |  | 50386 | NaN | 0.1425 | No |
| 1914 | ENSG00000236005 | AL389915.1 |  |  | 50400 | NaN | 0.1422 | No |
| 1915 | ENSG00000239872 | RPL35AP19 |  |  | 50435 | NaN | 0.1416 | No |
| 1916 | ENSG00000242991 | RPL7P40 |  |  | 50469 | NaN | 0.1411 | No |
| 1917 | ENSG00000237929 | RPL31P3 |  |  | 50522 | NaN | 0.1402 | No |
| 1918 | ENSG00000226356 | RPS6P20 |  |  | 50524 | NaN | 0.1402 | No |
| 1919 | ENSG00000276576 | MRPL30P1 |  |  | 50530 | NaN | 0.1401 | No |
| 1920 | ENSG00000237816 | RPL21P109 |  |  | 50542 | NaN | 0.1399 | No |
| 1921 | ENSG00000230659 | AL451074.5 |  |  | 50543 | NaN | 0.1399 | No |
| 1922 | ENSG00000243806 | RPL7P18 |  |  | 50559 | NaN | 0.1396 | No |
| 1923 | ENSG00000233353 | RPS7P9 |  |  | 50573 | NaN | 0.1394 | No |
| 1924 | ENSG00000233357 | RPL30P15 |  |  | 50575 | NaN | 0.1394 | No |
| 1925 | ENSG00000258965 | AC044913.1 |  |  | 50590 | NaN | 0.1392 | No |
| 1926 | ENSG00000236722 | AC116036.1 |  |  | 50616 | NaN | 0.1387 | No |
| 1927 | ENSG00000270682 | AP003357.1 |  |  | 50620 | NaN | 0.1387 | No |
| 1928 | ENSG00000232403 | AC009501.2 |  |  | 50624 | NaN | 0.1387 | No |
| 1929 | ENSG00000224315 | RPL7P7 |  |  | 50646 | NaN | 0.1383 | No |
| 1930 | ENSG00000216368 | AL136226.1 |  |  | 50660 | NaN | 0.1381 | No |
| 1931 | ENSG00000234496 | MRPS21P1 |  |  | 50661 | NaN | 0.1381 | No |
| 1932 | ENSG00000250747 | AC116345.2 |  |  | 50703 | NaN | 0.1374 | No |
| 1933 | ENSG00000258519 | AL121579.1 |  |  | 50706 | NaN | 0.1373 | No |
| 1934 | ENSG00000227138 | AL161788.1 |  |  | 50717 | NaN | 0.1372 | No |
| 1935 | ENSG00000242206 | RPS26P35 |  |  | 50730 | NaN | 0.1370 | No |
| 1936 | ENSG00000254012 | RPL10P8 |  |  | 50731 | NaN | 0.1370 | No |
| 1937 | ENSG00000282912 | AC108724.2 |  |  | 50734 | NaN | 0.1370 | No |
| 1938 | ENSG00000244270 | RPL32P29 |  |  | 50743 | NaN | 0.1368 | No |
| 1939 | ENSG00000230451 | RPS29P6 |  |  | 50755 | NaN | 0.1366 | No |
| 1940 | ENSG00000228374 | AC012497.1 |  |  | 50760 | NaN | 0.1366 | No |
| 1941 | ENSG00000239253 | RPS4XP23 |  |  | 50762 | NaN | 0.1366 | No |
| 1942 | ENSG00000239257 | RPL23AP1 |  |  | 50764 | NaN | 0.1366 | No |
| 1943 | ENSG00000239256 | RPL35AP35 |  |  | 50765 | NaN | 0.1366 | No |
| 1944 | ENSG00000235133 | RPL35AP22 |  |  | 50780 | NaN | 0.1363 | No |
| 1945 | ENSG00000229423 | RPL27AP8 |  |  | 50793 | NaN | 0.1361 | No |
| 1946 | ENSG00000231837 | RPS7P2 |  |  | 50811 | NaN | 0.1358 | No |
| 1947 | ENSG00000224672 | RPL17P10 |  |  | 50827 | NaN | 0.1356 | No |
| 1948 | ENSG00000230282 | RPL7P61 |  |  | 50907 | NaN | 0.1342 | No |
| 1949 | ENSG00000237887 | RPL23AP32 |  |  | 50926 | NaN | 0.1339 | No |
| 1950 | ENSG00000237888 | AC087650.1 |  |  | 50930 | NaN | 0.1338 | No |
| 1951 | ENSG00000231735 | AC092573.1 |  |  | 50931 | NaN | 0.1338 | No |
| 1952 | ENSG00000237774 | AC126615.1 |  |  | 50940 | NaN | 0.1337 | No |
| 1953 | ENSG00000232612 | RPL31P35 |  |  | 50987 | NaN | 0.1329 | No |
| 1954 | ENSG00000236913 | AC025750.2 |  |  | 51011 | NaN | 0.1325 | No |
| 1955 | ENSG00000229742 | AC092809.1 |  |  | 51057 | NaN | 0.1317 | No |
| 1956 | ENSG00000231293 | RPL36AP6 |  |  | 51061 | NaN | 0.1317 | No |
| 1957 | ENSG00000266561 | AC011195.1 |  |  | 51085 | NaN | 0.1313 | No |
| 1958 | ENSG00000227586 | RPS26P39 |  |  | 51098 | NaN | 0.1311 | No |
| 1959 | ENSG00000125385 | AL161781.1 |  |  | 51141 | NaN | 0.1304 | No |
| 1960 | ENSG00000284803 | AC245033.4 |  |  | 51153 | NaN | 0.1302 | No |
| 1961 | ENSG00000243672 | RPS3AP40 |  |  | 51166 | NaN | 0.1300 | No |
| 1962 | ENSG00000226972 | RPL12P19 |  |  | 51188 | NaN | 0.1296 | No |
| 1963 | ENSG00000261234 | AC025281.1 |  |  | 51196 | NaN | 0.1295 | No |
| 1964 | ENSG00000240320 | RPS29P27 |  |  | 51201 | NaN | 0.1294 | No |
| 1965 | ENSG00000244571 | RPS6P4 |  |  | 51238 | NaN | 0.1288 | No |
| 1966 | ENSG00000234067 | RPL5P10 |  |  | 51287 | NaN | 0.1280 | No |
| 1967 | ENSG00000230980 | RPL36AP39 |  |  | 51292 | NaN | 0.1279 | No |
| 1968 | ENSG00000230988 | RPL23AP11 |  |  | 51295 | NaN | 0.1279 | No |
| 1969 | ENSG00000243705 | AC010531.2 |  |  | 51328 | NaN | 0.1273 | No |
| 1970 | ENSG00000243538 | RPS26P28 |  |  | 51333 | NaN | 0.1273 | No |
| 1971 | ENSG00000249019 | AC108471.1 |  |  | 51355 | NaN | 0.1269 | No |
| 1972 | ENSG00000214298 | MRPS21P6 |  |  | 51357 | NaN | 0.1269 | No |
| 1973 | ENSG00000243507 | AC005908.1 |  |  | 51381 | NaN | 0.1265 | No |
| 1974 | ENSG00000213620 | AL121657.1 |  |  | 51387 | NaN | 0.1264 | No |
| 1975 | ENSG00000224627 | RPL37P10 |  |  | 51400 | NaN | 0.1262 | No |
| 1976 | ENSG00000243503 | AC090453.1 |  |  | 51412 | NaN | 0.1260 | No |
| 1977 | ENSG00000238085 | AL590682.1 |  |  | 51417 | NaN | 0.1260 | No |
| 1978 | ENSG00000223538 | AC079602.1 |  |  | 51422 | NaN | 0.1259 | No |
| 1979 | ENSG00000226573 | AC003071.2 |  |  | 51467 | NaN | 0.1251 | No |
| 1980 | ENSG00000236976 | AC091685.2 |  |  | 51477 | NaN | 0.1250 | No |
| 1981 | ENSG00000283393 | AC068205.3 |  |  | 51501 | NaN | 0.1246 | No |
| 1982 | ENSG00000226395 | MRPS21P5 |  |  | 51504 | NaN | 0.1246 | No |
| 1983 | ENSG00000231211 | RPL17P49 |  |  | 51529 | NaN | 0.1241 | No |
| 1984 | ENSG00000256243 | RPL7AP3 |  |  | 51543 | NaN | 0.1239 | No |
| 1985 | ENSG00000240427 | RPS26P34 |  |  | 51579 | NaN | 0.1233 | No |
| 1986 | ENSG00000240443 | RPS10P20 |  |  | 51605 | NaN | 0.1229 | No |
| 1987 | ENSG00000240669 | AC023424.1 |  |  | 51639 | NaN | 0.1223 | No |
| 1988 | ENSG00000268116 | AC073539.2 |  |  | 51666 | NaN | 0.1219 | No |
| 1989 | ENSG00000258796 | AL133241.1 |  |  | 51697 | NaN | 0.1213 | No |
| 1990 | ENSG00000229765 | AL354868.1 |  |  | 51721 | NaN | 0.1209 | No |
| 1991 | ENSG00000232032 | AC079781.2 |  |  | 51732 | NaN | 0.1208 | No |
| 1992 | ENSG00000213285 | RPL23AP45 |  |  | 51736 | NaN | 0.1207 | No |
| 1993 | ENSG00000233565 | AC007690.1 |  |  | 51793 | NaN | 0.1198 | No |
| 1994 | ENSG00000232429 | RPL21P131 |  |  | 51804 | NaN | 0.1196 | No |
| 1995 | ENSG00000227982 | RPL7P30 |  |  | 51817 | NaN | 0.1194 | No |
| 1996 | ENSG00000266190 | AC090287.1 |  |  | 51824 | NaN | 0.1193 | No |
| 1997 | ENSG00000242087 | RPL36AP41 |  |  | 51827 | NaN | 0.1193 | No |
| 1998 | ENSG00000250547 | AC079238.1 |  |  | 51852 | NaN | 0.1188 | No |
| 1999 | ENSG00000228453 | RPS15AP9 |  |  | 51853 | NaN | 0.1189 | No |
| 2000 | ENSG00000241423 | RPL21P103 |  |  | 51863 | NaN | 0.1187 | No |
| 2001 | ENSG00000225831 | RPS18P1 |  |  | 51890 | NaN | 0.1183 | No |
| 2002 | ENSG00000229326 | AC069154.1 |  |  | 51893 | NaN | 0.1182 | No |
| 2003 | ENSG00000242899 | RPL7P16 |  |  | 51903 | NaN | 0.1181 | No |
| 2004 | ENSG00000255253 | AC068587.3 |  |  | 51967 | NaN | 0.1170 | No |
| 2005 | ENSG00000243385 | AC094085.1 |  |  | 51979 | NaN | 0.1168 | No |
| 2006 | ENSG00000243388 | RPL3P3 |  |  | 51980 | NaN | 0.1168 | No |
| 2007 | ENSG00000237806 | FAUP2 |  |  | 51990 | NaN | 0.1166 | No |
| 2008 | ENSG00000270916 | RPL12P50 |  |  | 52004 | NaN | 0.1164 | No |
| 2009 | ENSG00000232568 | RPL23AP35 |  |  | 52049 | NaN | 0.1157 | No |
| 2010 | ENSG00000223688 | RPS24P14 |  |  | 52063 | NaN | 0.1154 | No |
| 2011 | ENSG00000228191 | AL512326.2 |  |  | 52088 | NaN | 0.1150 | No |
| 2012 | ENSG00000230871 | RPS6P23 |  |  | 52103 | NaN | 0.1148 | No |
| 2013 | ENSG00000240760 | RPL31P56 |  |  | 52106 | NaN | 0.1148 | No |
| 2014 | ENSG00000260603 | AC133539.1 |  |  | 52112 | NaN | 0.1147 | No |
| 2015 | ENSG00000235962 | RPL7AP53 |  |  | 52157 | NaN | 0.1139 | No |
| 2016 | ENSG00000239198 | RPL5P22 |  |  | 52200 | NaN | 0.1132 | No |
| 2017 | ENSG00000239199 | RPL21P6 |  |  | 52201 | NaN | 0.1132 | No |
| 2018 | ENSG00000243939 | AC090142.2 |  |  | 52230 | NaN | 0.1127 | No |
| 2019 | ENSG00000233900 | RPL17P35 |  |  | 52287 | NaN | 0.1117 | No |
| 2020 | ENSG00000253332 | AP005902.2 |  |  | 52322 | NaN | 0.1111 | No |
| 2021 | ENSG00000240827 | AC021660.1 |  |  | 52323 | NaN | 0.1111 | No |
| 2022 | ENSG00000232496 | RPL3P12 |  |  | 52328 | NaN | 0.1111 | No |
| 2023 | ENSG00000228399 | AL109741.2 |  |  | 52332 | NaN | 0.1110 | No |
| 2024 | ENSG00000243023 | UBA52P3 |  |  | 52355 | NaN | 0.1107 | No |
| 2025 | ENSG00000240590 | RPSAP48 |  |  | 52382 | NaN | 0.1102 | No |
| 2026 | ENSG00000240047 | RPS3AP32 |  |  | 52398 | NaN | 0.1100 | No |
| 2027 | ENSG00000213233 | RPL23AP62 |  |  | 52418 | NaN | 0.1096 | No |
| 2028 | ENSG00000218749 | AL033519.1 |  |  | 52429 | NaN | 0.1095 | No |
| 2029 | ENSG00000232719 | AC007272.1 |  |  | 52437 | NaN | 0.1093 | No |
| 2030 | ENSG00000237551 | AC096775.1 |  |  | 52462 | NaN | 0.1089 | No |
| 2031 | ENSG00000244229 | RPL26P35 |  |  | 52475 | NaN | 0.1087 | No |
| 2032 | ENSG00000225662 | AC012519.1 |  |  | 52477 | NaN | 0.1087 | No |
| 2033 | ENSG00000225669 | RPL23AP27 |  |  | 52479 | NaN | 0.1087 | No |
| 2034 | ENSG00000213461 | AL672212.1 |  |  | 52524 | NaN | 0.1079 | No |
| 2035 | ENSG00000218265 | RPS4XP7 |  |  | 52537 | NaN | 0.1077 | No |
| 2036 | ENSG00000226532 | AL022718.1 |  |  | 52541 | NaN | 0.1077 | No |
| 2037 | ENSG00000241651 | AC099340.1 |  |  | 52573 | NaN | 0.1072 | No |
| 2038 | ENSG00000240201 | AL133372.1 |  |  | 52599 | NaN | 0.1067 | No |
| 2039 | ENSG00000255122 | AC068587.2 |  |  | 52606 | NaN | 0.1066 | No |
| 2040 | ENSG00000267207 | AC005884.1 |  |  | 52631 | NaN | 0.1062 | No |
| 2041 | ENSG00000232848 | AL034417.1 |  |  | 52659 | NaN | 0.1057 | No |
| 2042 | ENSG00000242837 | RPL21P13 |  |  | 52681 | NaN | 0.1054 | No |
| 2043 | ENSG00000236171 | RPL12P26 |  |  | 52705 | NaN | 0.1050 | No |
| 2044 | ENSG00000227271 | RPL39P25 |  |  | 52709 | NaN | 0.1049 | No |
| 2045 | ENSG00000214362 | RPS3AP36 |  |  | 52716 | NaN | 0.1048 | No |
| 2046 | ENSG00000271560 | AP001330.4 |  |  | 52733 | NaN | 0.1046 | No |
| 2047 | ENSG00000235482 | RPL21P135 |  |  | 52745 | NaN | 0.1044 | No |
| 2048 | ENSG00000232105 | RPL32P28 |  |  | 52758 | NaN | 0.1042 | No |
| 2049 | ENSG00000223676 | RPL34P26 |  |  | 52764 | NaN | 0.1041 | No |
| 2050 | ENSG00000223671 | RPL34P3 |  |  | 52766 | NaN | 0.1041 | No |
| 2051 | ENSG00000226439 | AC108161.1 |  |  | 52812 | NaN | 0.1033 | No |
| 2052 | ENSG00000227747 | AC096631.2 |  |  | 52816 | NaN | 0.1033 | No |
| 2053 | ENSG00000266944 | AC005262.1 |  |  | 52853 | NaN | 0.1026 | No |
| 2054 | ENSG00000242660 | AC015911.1 |  |  | 52856 | NaN | 0.1026 | No |
| 2055 | ENSG00000214259 | AC018695.1 |  |  | 52868 | NaN | 0.1024 | No |
| 2056 | ENSG00000240881 | AC068472.1 |  |  | 52950 | NaN | 0.1010 | No |
| 2057 | ENSG00000235208 | AL135938.1 |  |  | 52998 | NaN | 0.1002 | No |
| 2058 | ENSG00000219814 | RPL23AP47 |  |  | 53052 | NaN | 0.0993 | No |
| 2059 | ENSG00000253153 | AP001207.1 |  |  | 53076 | NaN | 0.0989 | No |
| 2060 | ENSG00000213343 | RPL21P18 |  |  | 53088 | NaN | 0.0987 | No |
| 2061 | ENSG00000236327 | AL831737.1 |  |  | 53105 | NaN | 0.0984 | No |
| 2062 | ENSG00000213750 | AP002982.1 |  |  | 53119 | NaN | 0.0982 | No |
| 2063 | ENSG00000235673 | AL929472.4 |  |  | 53134 | NaN | 0.0979 | No |
| 2064 | ENSG00000214878 | RPL5P31 |  |  | 53194 | NaN | 0.0969 | No |
| 2065 | ENSG00000241228 | RPL12P31 |  |  | 53213 | NaN | 0.0966 | No |
| 2066 | ENSG00000225971 | AC003071.1 |  |  | 53217 | NaN | 0.0966 | No |
| 2067 | ENSG00000244604 | AC025518.1 |  |  | 53232 | NaN | 0.0963 | No |
| 2068 | ENSG00000242608 | RPS23P5 |  |  | 53246 | NaN | 0.0961 | No |
| 2069 | ENSG00000231361 | RPS29P23 |  |  | 53270 | NaN | 0.0957 | No |
| 2070 | ENSG00000231362 | AC069271.1 |  |  | 53271 | NaN | 0.0957 | No |
| 2071 | ENSG00000231366 | RPS26P40 |  |  | 53273 | NaN | 0.0957 | No |
| 2072 | ENSG00000280012 | RPL23AP61 |  |  | 53290 | NaN | 0.0954 | No |
| 2073 | ENSG00000240513 | AL049830.2 |  |  | 53330 | NaN | 0.0948 | No |
| 2074 | ENSG00000270842 | AC073413.2 |  |  | 53367 | NaN | 0.0941 | No |
| 2075 | ENSG00000224553 | AC008065.1 |  |  | 53399 | NaN | 0.0936 | No |
| 2076 | ENSG00000234658 | AC013429.1 |  |  | 53420 | NaN | 0.0933 | No |
| 2077 | ENSG00000230935 | RPS3P1 |  |  | 53424 | NaN | 0.0932 | No |
| 2078 | ENSG00000177350 | RPL13AP3 |  |  | 53431 | NaN | 0.0931 | No |
| 2079 | ENSG00000215381 | AL109936.1 |  |  | 53439 | NaN | 0.0930 | No |
| 2080 | ENSG00000260113 | AC093249.1 |  |  | 53499 | NaN | 0.0920 | No |
| 2081 | ENSG00000239327 | AL163195.1 |  |  | 53511 | NaN | 0.0918 | No |
| 2082 | ENSG00000236512 | AL390026.1 |  |  | 53513 | NaN | 0.0918 | No |
| 2083 | ENSG00000226836 | AC018437.1 |  |  | 53518 | NaN | 0.0917 | No |
| 2084 | ENSG00000185078 | AC093698.1 |  |  | 53530 | NaN | 0.0915 | No |
| 2085 | ENSG00000213122 | RPL23AP46 |  |  | 53538 | NaN | 0.0914 | No |
| 2086 | ENSG00000213128 | RPL32P31 |  |  | 53540 | NaN | 0.0914 | No |
| 2087 | ENSG00000239517 | AC116337.2 |  |  | 53541 | NaN | 0.0914 | No |
| 2088 | ENSG00000238055 | Z98742.3 |  |  | 53544 | NaN | 0.0914 | No |
| 2089 | ENSG00000227875 | RPL23AP89 |  |  | 53557 | NaN | 0.0912 | No |
| 2090 | ENSG00000226292 | AL606495.1 |  |  | 53565 | NaN | 0.0911 | No |
| 2091 | ENSG00000226580 | RPL39P40 |  |  | 53587 | NaN | 0.0907 | No |
| 2092 | ENSG00000226297 | AC007677.1 |  |  | 53589 | NaN | 0.0907 | No |
| 2093 | ENSG00000243141 | RPL23AP66 |  |  | 53632 | NaN | 0.0900 | No |
| 2094 | ENSG00000240494 | RPS12P28 |  |  | 53670 | NaN | 0.0893 | No |
| 2095 | ENSG00000240721 | RPS4XP15 |  |  | 53685 | NaN | 0.0891 | No |
| 2096 | ENSG00000235294 | RPL7AP25 |  |  | 53694 | NaN | 0.0889 | No |
| 2097 | ENSG00000241451 | RPS27P22 |  |  | 53712 | NaN | 0.0887 | No |
| 2098 | ENSG00000234751 | AP002381.1 |  |  | 53742 | NaN | 0.0881 | No |
| 2099 | ENSG00000215264 | RPL10AP3 |  |  | 53798 | NaN | 0.0872 | No |
| 2100 | ENSG00000226216 | RPS12P5 |  |  | 53815 | NaN | 0.0869 | No |
| 2101 | ENSG00000274319 | RPSAP73 |  |  | 53828 | NaN | 0.0867 | No |
| 2102 | ENSG00000244266 | AC090058.1 |  |  | 53855 | NaN | 0.0863 | No |
| 2103 | ENSG00000228301 | RPL7P55 |  |  | 53889 | NaN | 0.0857 | No |
| 2104 | ENSG00000240121 | RPS27P20 |  |  | 53907 | NaN | 0.0854 | No |
| 2105 | ENSG00000240125 | RPL23AP75 |  |  | 53909 | NaN | 0.0854 | No |
| 2106 | ENSG00000244113 | AC079193.1 |  |  | 53949 | NaN | 0.0847 | No |
| 2107 | ENSG00000240631 | RPL30P12 |  |  | 53953 | NaN | 0.0847 | No |
| 2108 | ENSG00000232341 | RPL4P2 |  |  | 53996 | NaN | 0.0839 | No |
| 2109 | ENSG00000219222 | RPL12P47 |  |  | 54015 | NaN | 0.0836 | No |
| 2110 | ENSG00000220868 | MRPL35P1 |  |  | 54040 | NaN | 0.0832 | No |
| 2111 | ENSG00000228000 | RPL7AP65 |  |  | 54048 | NaN | 0.0831 | No |
| 2112 | ENSG00000273806 | AL137847.3 |  |  | 54055 | NaN | 0.0830 | No |
| 2113 | ENSG00000276810 | AC244093.2 |  |  | 54073 | NaN | 0.0827 | No |
| 2114 | ENSG00000267752 | RPS10P27 |  |  | 54074 | NaN | 0.0827 | No |
| 2115 | ENSG00000233135 | RPS27P18 |  |  | 54130 | NaN | 0.0818 | No |
| 2116 | ENSG00000242320 | RPL21P126 |  |  | 54138 | NaN | 0.0816 | No |
| 2117 | ENSG00000220069 | RPL7P27 |  |  | 54158 | NaN | 0.0813 | No |
| 2118 | ENSG00000223445 | RPL6P21 |  |  | 54162 | NaN | 0.0813 | No |
| 2119 | ENSG00000224797 | AL359092.1 |  |  | 54165 | NaN | 0.0812 | No |
| 2120 | ENSG00000239212 | RPL6P7 |  |  | 54166 | NaN | 0.0813 | No |
| 2121 | ENSG00000239671 | AC100793.1 |  |  | 54186 | NaN | 0.0809 | No |
| 2122 | ENSG00000225832 | AL035090.1 |  |  | 54225 | NaN | 0.0803 | No |
| 2123 | ENSG00000228807 | MRPS18CP6 |  |  | 54249 | NaN | 0.0799 | No |
| 2124 | ENSG00000250253 | AC117522.3 |  |  | 54270 | NaN | 0.0795 | No |
| 2125 | ENSG00000174572 | RPL10P2 |  |  | 54301 | NaN | 0.0790 | No |
| 2126 | ENSG00000244743 | AC087588.1 |  |  | 54309 | NaN | 0.0789 | No |
| 2127 | ENSG00000244740 | AC092991.1 |  |  | 54328 | NaN | 0.0786 | No |
| 2128 | ENSG00000228489 | RPL21P50 |  |  | 54380 | NaN | 0.0777 | No |
| 2129 | ENSG00000232069 | RPS29P28 |  |  | 54385 | NaN | 0.0776 | No |
| 2130 | ENSG00000240419 | AC022206.1 |  |  | 54402 | NaN | 0.0774 | No |
| 2131 | ENSG00000265626 | AC139085.1 |  |  | 54409 | NaN | 0.0773 | No |
| 2132 | ENSG00000231005 | RPL39P39 |  |  | 54420 | NaN | 0.0771 | No |
| 2133 | ENSG00000240412 | RPL5P15 |  |  | 54444 | NaN | 0.0767 | No |
| 2134 | ENSG00000230350 | RPL35AP3 |  |  | 54452 | NaN | 0.0766 | No |
| 2135 | ENSG00000231586 | RPS23P9 |  |  | 54467 | NaN | 0.0763 | No |
| 2136 | ENSG00000214047 | RPS11P7 |  |  | 54468 | NaN | 0.0763 | No |
| 2137 | ENSG00000240518 | AC116353.1 |  |  | 54500 | NaN | 0.0758 | No |
| 2138 | ENSG00000233072 | RPS15AP6 |  |  | 54514 | NaN | 0.0756 | No |
| 2139 | ENSG00000233071 | RPSAP59 |  |  | 54515 | NaN | 0.0756 | No |
| 2140 | ENSG00000236594 | RPS27AP14 |  |  | 54529 | NaN | 0.0754 | No |
| 2141 | ENSG00000243930 | RPS12P21 |  |  | 54544 | NaN | 0.0751 | No |
| 2142 | ENSG00000215063 | RPL21P2 |  |  | 54608 | NaN | 0.0740 | No |
| 2143 | ENSG00000250475 | AC017037.2 |  |  | 54609 | NaN | 0.0740 | No |
| 2144 | ENSG00000244527 | AC022795.1 |  |  | 54626 | NaN | 0.0738 | No |
| 2145 | ENSG00000227361 | RPS24P7 |  |  | 54637 | NaN | 0.0736 | No |
| 2146 | ENSG00000230198 | RPL37P4 |  |  | 54697 | NaN | 0.0726 | No |
| 2147 | ENSG00000243859 | RPL5P17 |  |  | 54723 | NaN | 0.0721 | No |
| 2148 | ENSG00000243855 | RPL12P5 |  |  | 54727 | NaN | 0.0721 | No |
| 2149 | ENSG00000270966 | AC087763.1 |  |  | 54751 | NaN | 0.0717 | No |
| 2150 | ENSG00000270385 | AC133539.2 |  |  | 54776 | NaN | 0.0713 | No |
| 2151 | ENSG00000223409 | RPL17P17 |  |  | 54808 | NaN | 0.0708 | No |
| 2152 | ENSG00000184961 | RPL7AP49 |  |  | 54817 | NaN | 0.0706 | No |
| 2153 | ENSG00000226166 | AC244670.1 |  |  | 54827 | NaN | 0.0705 | No |
| 2154 | ENSG00000214074 | RPL23AP39 |  |  | 54829 | NaN | 0.0705 | No |
| 2155 | ENSG00000224348 | AL592076.1 |  |  | 54882 | NaN | 0.0696 | No |
| 2156 | ENSG00000242276 | RPL5P3 |  |  | 54883 | NaN | 0.0696 | No |
| 2157 | ENSG00000242279 | AP000893.1 |  |  | 54885 | NaN | 0.0695 | No |
| 2158 | ENSG00000242278 | AC074051.1 |  |  | 54886 | NaN | 0.0696 | No |
| 2159 | ENSG00000239201 | RPL21P106 |  |  | 54907 | NaN | 0.0692 | No |
| 2160 | ENSG00000233518 | RPL7P35 |  |  | 54984 | NaN | 0.0679 | No |
| 2161 | ENSG00000226715 | LINC01709 |  |  | 55003 | NaN | 0.0676 | No |
| 2162 | ENSG00000214676 | RPL9P16 |  |  | 55012 | NaN | 0.0674 | No |
| 2163 | ENSG00000240724 | RPS12P15 |  |  | 55013 | NaN | 0.0675 | No |
| 2164 | ENSG00000214671 | RPL6P12 |  |  | 55022 | NaN | 0.0673 | No |
| 2165 | ENSG00000240992 | RPS23P4 |  |  | 55038 | NaN | 0.0671 | No |
| 2166 | ENSG00000241204 | AC022730.2 |  |  | 55048 | NaN | 0.0669 | No |
| 2167 | ENSG00000214908 | AL353678.1 |  |  | 55100 | NaN | 0.0660 | No |
| 2168 | ENSG00000237783 | AL356441.2 |  |  | 55116 | NaN | 0.0658 | No |
| 2169 | ENSG00000237784 | AC097713.2 |  |  | 55118 | NaN | 0.0658 | No |
| 2170 | ENSG00000219274 | RPS20P2 |  |  | 55133 | NaN | 0.0655 | No |
| 2171 | ENSG00000214089 | RPL9P21 |  |  | 55144 | NaN | 0.0654 | No |
| 2172 | ENSG00000232430 | RPL31P15 |  |  | 55161 | NaN | 0.0651 | No |
| 2173 | ENSG00000233206 | RPS3AP1 |  |  | 55178 | NaN | 0.0648 | No |
| 2174 | ENSG00000225405 | RPS15AP17 |  |  | 55191 | NaN | 0.0646 | No |
| 2175 | ENSG00000271222 | AC091152.3 |  |  | 55200 | NaN | 0.0645 | No |
| 2176 | ENSG00000272657 | AP000317.2 |  |  | 55218 | NaN | 0.0642 | No |
| 2177 | ENSG00000249262 | AC112250.1 |  |  | 55226 | NaN | 0.0641 | No |
| 2178 | ENSG00000275839 | AC243654.2 |  |  | 55246 | NaN | 0.0637 | No |
| 2179 | ENSG00000230841 | RPL23AP15 |  |  | 55256 | NaN | 0.0636 | No |
| 2180 | ENSG00000235266 | RPL22P17 |  |  | 55286 | NaN | 0.0631 | No |
| 2181 | ENSG00000241045 | RPL7P43 |  |  | 55298 | NaN | 0.0629 | No |
| 2182 | ENSG00000267661 | AP001269.3 |  |  | 55311 | NaN | 0.0627 | No |
| 2183 | ENSG00000267669 | AC098847.1 |  |  | 55313 | NaN | 0.0627 | No |
| 2184 | ENSG00000242100 | RPL9P32 |  |  | 55315 | NaN | 0.0627 | No |
| 2185 | ENSG00000237540 | RPL36AP36 |  |  | 55370 | NaN | 0.0617 | No |
| 2186 | ENSG00000240371 | RPS4XP13 |  |  | 55385 | NaN | 0.0615 | No |
| 2187 | ENSG00000225343 | RPL21P66 |  |  | 55405 | NaN | 0.0612 | No |
| 2188 | ENSG00000229824 | RPL12P34 |  |  | 55413 | NaN | 0.0611 | No |
| 2189 | ENSG00000244019 | RPS2P24 |  |  | 55417 | NaN | 0.0610 | No |
| 2190 | ENSG00000270512 | AC005377.1 |  |  | 55435 | NaN | 0.0607 | No |
| 2191 | ENSG00000241808 | RPS15AP34 |  |  | 55460 | NaN | 0.0603 | No |
| 2192 | ENSG00000231622 | RPS29P7 |  |  | 55523 | NaN | 0.0592 | No |
| 2193 | ENSG00000230835 | AP001187.1 |  |  | 55566 | NaN | 0.0585 | No |
| 2194 | ENSG00000270892 | AC006077.1 |  |  | 55592 | NaN | 0.0581 | No |
| 2195 | ENSG00000240873 | RPS29P22 |  |  | 55596 | NaN | 0.0580 | No |
| 2196 | ENSG00000216636 | RPL7P25 |  |  | 55599 | NaN | 0.0580 | No |
| 2197 | ENSG00000223568 | RPL3P11 |  |  | 55611 | NaN | 0.0578 | No |
| 2198 | ENSG00000169253 | AL669983.1 |  |  | 55633 | NaN | 0.0574 | No |
| 2199 | ENSG00000239532 | AC108022.1 |  |  | 55641 | NaN | 0.0573 | No |
| 2200 | ENSG00000244251 | AC013356.1 |  |  | 55668 | NaN | 0.0569 | No |
| 2201 | ENSG00000244259 | AP000797.2 |  |  | 55672 | NaN | 0.0568 | No |
| 2202 | ENSG00000243844 | RPL17P33 |  |  | 55760 | NaN | 0.0553 | No |
| 2203 | ENSG00000243761 | AC023194.2 |  |  | 55780 | NaN | 0.0550 | No |
| 2204 | ENSG00000242417 | RPL18P1 |  |  | 55782 | NaN | 0.0550 | No |
| 2205 | ENSG00000240237 | AF305872.1 |  |  | 55813 | NaN | 0.0545 | No |
| 2206 | ENSG00000226888 | RPLP1P10 |  |  | 55815 | NaN | 0.0544 | No |
| 2207 | ENSG00000240231 | RPS27P29 |  |  | 55816 | NaN | 0.0545 | No |
| 2208 | ENSG00000240238 | AC092754.1 |  |  | 55817 | NaN | 0.0545 | No |
| 2209 | ENSG00000249510 | AP003478.1 |  |  | 55820 | NaN | 0.0544 | No |
| 2210 | ENSG00000241183 | AC009022.2 |  |  | 55844 | NaN | 0.0540 | No |
| 2211 | ENSG00000215263 | AC025750.1 |  |  | 55855 | NaN | 0.0539 | No |
| 2212 | ENSG00000229806 | RPS15P5 |  |  | 55893 | NaN | 0.0532 | No |
| 2213 | ENSG00000232238 | RPS29P8 |  |  | 55902 | NaN | 0.0531 | No |
| 2214 | ENSG00000226217 | RPL19P1 |  |  | 55934 | NaN | 0.0526 | No |
| 2215 | ENSG00000239600 | AP000797.1 |  |  | 55941 | NaN | 0.0525 | No |
| 2216 | ENSG00000215016 | RPL24P7 |  |  | 55950 | NaN | 0.0523 | No |
| 2217 | ENSG00000267664 | RPL17P45 |  |  | 55955 | NaN | 0.0523 | No |
| 2218 | ENSG00000213041 | AC093422.1 |  |  | 56002 | NaN | 0.0515 | No |
| 2219 | ENSG00000206147 | RPL23AP57 |  |  | 56048 | NaN | 0.0507 | No |
| 2220 | ENSG00000239701 | AC006512.1 |  |  | 56060 | NaN | 0.0505 | No |
| 2221 | ENSG00000229682 | AC114755.2 |  |  | 56079 | NaN | 0.0502 | No |
| 2222 | ENSG00000213867 | AL049830.1 |  |  | 56089 | NaN | 0.0500 | No |
| 2223 | ENSG00000225846 | AC112719.1 |  |  | 56092 | NaN | 0.0500 | No |
| 2224 | ENSG00000266915 | MRPS5P4 |  |  | 56123 | NaN | 0.0495 | No |
| 2225 | ENSG00000255286 | AP000907.1 |  |  | 56166 | NaN | 0.0488 | No |
| 2226 | ENSG00000226407 | RPL23P11 |  |  | 56196 | NaN | 0.0483 | No |
| 2227 | ENSG00000248895 | AC068531.1 |  |  | 56285 | NaN | 0.0467 | No |
| 2228 | ENSG00000231139 | AC104843.2 |  |  | 56311 | NaN | 0.0463 | No |
| 2229 | ENSG00000212932 | RPL23AP4 |  |  | 56369 | NaN | 0.0453 | No |
| 2230 | ENSG00000218069 | RSL24D1P1 |  |  | 56379 | NaN | 0.0451 | No |
| 2231 | ENSG00000241282 | RPL34P33 |  |  | 56390 | NaN | 0.0450 | No |
| 2232 | ENSG00000220110 | AL080315.1 |  |  | 56429 | NaN | 0.0443 | No |
| 2233 | ENSG00000235182 | AC104333.1 |  |  | 56438 | NaN | 0.0442 | No |
| 2234 | ENSG00000244099 | RPS23P3 |  |  | 56455 | NaN | 0.0439 | No |
| 2235 | ENSG00000244097 | RPS4XP17 |  |  | 56458 | NaN | 0.0439 | No |
| 2236 | ENSG00000242670 | RPL22P13 |  |  | 56497 | NaN | 0.0432 | No |
| 2237 | ENSG00000242675 | RPS16P9 |  |  | 56499 | NaN | 0.0432 | No |
| 2238 | ENSG00000233555 | AL591438.1 |  |  | 56543 | NaN | 0.0425 | No |
| 2239 | ENSG00000214263 | RPSAP53 |  |  | 56570 | NaN | 0.0420 | No |
| 2240 | ENSG00000241344 | RPL21P47 |  |  | 56596 | NaN | 0.0416 | No |
| 2241 | ENSG00000213605 | AC111200.1 |  |  | 56649 | NaN | 0.0407 | No |
| 2242 | ENSG00000234080 | AL596275.1 |  |  | 56672 | NaN | 0.0403 | No |
| 2243 | ENSG00000225267 | RPL8P2 |  |  | 56673 | NaN | 0.0403 | No |
| 2244 | ENSG00000213987 | AC093151.1 |  |  | 56691 | NaN | 0.0400 | No |
| 2245 | ENSG00000243469 | RPL7P51 |  |  | 56692 | NaN | 0.0400 | No |
| 2246 | ENSG00000213179 | RPL17P41 |  |  | 56750 | NaN | 0.0390 | No |
| 2247 | ENSG00000213172 | AL031985.1 |  |  | 56752 | NaN | 0.0390 | No |
| 2248 | ENSG00000216642 | AL136116.1 |  |  | 56762 | NaN | 0.0389 | No |
| 2249 | ENSG00000241105 | AC022730.1 |  |  | 56782 | NaN | 0.0385 | No |
| 2250 | ENSG00000239793 | AC098818.1 |  |  | 56795 | NaN | 0.0383 | No |
| 2251 | ENSG00000269228 | RPS27AP19 |  |  | 56823 | NaN | 0.0379 | No |
| 2252 | ENSG00000241950 | RPL29P23 |  |  | 56854 | NaN | 0.0373 | No |
| 2253 | ENSG00000231541 | RPS6P2 |  |  | 56867 | NaN | 0.0371 | No |
| 2254 | ENSG00000240776 | AC079944.1 |  |  | 56889 | NaN | 0.0368 | No |
| 2255 | ENSG00000237827 | RPS15AP29 |  |  | 56891 | NaN | 0.0368 | No |
| 2256 | ENSG00000270992 | AC024085.1 |  |  | 56937 | NaN | 0.0360 | No |
| 2257 | ENSG00000223604 | AC007394.1 |  |  | 56946 | NaN | 0.0358 | No |
| 2258 | ENSG00000274756 | AC243732.1 |  |  | 56953 | NaN | 0.0357 | No |
| 2259 | ENSG00000240919 | AC022034.3 |  |  | 56971 | NaN | 0.0355 | No |
| 2260 | ENSG00000232174 | AC113340.1 |  |  | 56976 | NaN | 0.0354 | No |
| 2261 | ENSG00000253429 | AP005660.1 |  |  | 56987 | NaN | 0.0352 | No |
| 2262 | ENSG00000242411 | AC011506.1 |  |  | 57019 | NaN | 0.0347 | No |
| 2263 | ENSG00000244441 | AC126182.2 |  |  | 57032 | NaN | 0.0345 | No |
| 2264 | ENSG00000259198 | AC020658.2 |  |  | 57048 | NaN | 0.0342 | No |
| 2265 | ENSG00000225196 | RPL10P17 |  |  | 57061 | NaN | 0.0340 | No |
| 2266 | ENSG00000230116 | AL445669.1 |  |  | 57062 | NaN | 0.0340 | No |
| 2267 | ENSG00000239686 | AL158801.1 |  |  | 57076 | NaN | 0.0338 | No |
| 2268 | ENSG00000243404 | RPL35AP32 |  |  | 57096 | NaN | 0.0335 | No |
| 2269 | ENSG00000230449 | RPL7P4 |  |  | 57110 | NaN | 0.0333 | No |
| 2270 | ENSG00000234415 | RPL5P7 |  |  | 57138 | NaN | 0.0328 | No |
| 2271 | ENSG00000238015 | AC104837.2 |  |  | 57139 | NaN | 0.0328 | No |
| 2272 | ENSG00000280969 | RPS4Y2 |  |  | 57143 | NaN | 0.0328 | No |
| 2273 | ENSG00000242255 | RPL39P34 |  |  | 57192 | NaN | 0.0319 | No |
| 2274 | ENSG00000232379 | RPL22P23 |  |  | 57197 | NaN | 0.0319 | No |
| 2275 | ENSG00000225289 | RPL29P29 |  |  | 57246 | NaN | 0.0310 | No |
| 2276 | ENSG00000219150 | AL603914.1 |  |  | 57266 | NaN | 0.0307 | No |
| 2277 | ENSG00000213726 | RPS2P52 |  |  | 57304 | NaN | 0.0301 | No |
| 2278 | ENSG00000228053 | AL158823.1 |  |  | 57346 | NaN | 0.0293 | No |
| 2279 | ENSG00000242936 | RPL30P6 |  |  | 57357 | NaN | 0.0292 | No |
| 2280 | ENSG00000239626 | RPSAP41 |  |  | 57361 | NaN | 0.0291 | No |
| 2281 | ENSG00000267398 | AC006130.2 |  |  | 57364 | NaN | 0.0291 | No |
| 2282 | ENSG00000243122 | AC016304.1 |  |  | 57369 | NaN | 0.0290 | No |
| 2283 | ENSG00000186008 | RPS4XP21 |  |  | 57379 | NaN | 0.0289 | No |
| 2284 | ENSG00000239481 | RPS3AP41 |  |  | 57387 | NaN | 0.0288 | No |
| 2285 | ENSG00000225922 | RPL21P92 |  |  | 57388 | NaN | 0.0288 | No |
| 2286 | ENSG00000216480 | AL078604.1 |  |  | 57436 | NaN | 0.0280 | No |
| 2287 | ENSG00000243314 | AC106707.2 |  |  | 57450 | NaN | 0.0277 | No |
| 2288 | ENSG00000230064 | AL772161.1 |  |  | 57486 | NaN | 0.0271 | No |
| 2289 | ENSG00000237593 | AL445220.1 |  |  | 57525 | NaN | 0.0265 | No |
| 2290 | ENSG00000237049 | RPL34P1 |  |  | 57540 | NaN | 0.0262 | No |
| 2291 | ENSG00000241612 | AC114728.1 |  |  | 57551 | NaN | 0.0261 | No |
| 2292 | ENSG00000239323 | AL163973.1 |  |  | 57554 | NaN | 0.0260 | No |
| 2293 | ENSG00000233747 | RPL36AP13 |  |  | 57555 | NaN | 0.0261 | No |
| 2294 | ENSG00000229814 | RPL35AP21 |  |  | 57598 | NaN | 0.0253 | No |
| 2295 | ENSG00000214484 | RPSAP28 |  |  | 57648 | NaN | 0.0245 | No |
| 2296 | ENSG00000233131 | AC096649.2 |  |  | 57654 | NaN | 0.0244 | No |
| 2297 | ENSG00000232881 | RPS10P21 |  |  | 57667 | NaN | 0.0242 | No |
| 2298 | ENSG00000236549 | RPS27AP7 |  |  | 57706 | NaN | 0.0235 | No |
| 2299 | ENSG00000235018 | AL137077.1 |  |  | 57726 | NaN | 0.0232 | No |
| 2300 | ENSG00000251354 | AC024451.1 |  |  | 57746 | NaN | 0.0229 | No |
| 2301 | ENSG00000189145 | AC092268.1 |  |  | 57767 | NaN | 0.0225 | No |
| 2302 | ENSG00000229197 | RPS15AP28 |  |  | 57811 | NaN | 0.0218 | No |
| 2303 | ENSG00000233873 | RPL7P44 |  |  | 57812 | NaN | 0.0218 | No |
| 2304 | ENSG00000229687 | AL049745.1 |  |  | 57825 | NaN | 0.0216 | No |
| 2305 | ENSG00000230636 | RPL36P1 |  |  | 57903 | NaN | 0.0202 | No |
| 2306 | ENSG00000243864 | RPS3AP50 |  |  | 57926 | NaN | 0.0199 | No |
| 2307 | ENSG00000232463 | AL359265.2 |  |  | 57979 | NaN | 0.0189 | No |
| 2308 | ENSG00000217041 | AL512430.2 |  |  | 58037 | NaN | 0.0180 | No |
| 2309 | ENSG00000236065 | AL020995.1 |  |  | 58046 | NaN | 0.0178 | No |
| 2310 | ENSG00000242229 | RPS3AP14 |  |  | 58077 | NaN | 0.0173 | No |
| 2311 | ENSG00000248716 | AC093815.1 |  |  | 58135 | NaN | 0.0163 | No |
| 2312 | ENSG00000225239 | RPL21P107 |  |  | 58153 | NaN | 0.0160 | No |
| 2313 | ENSG00000187472 | AL589826.1 |  |  | 58198 | NaN | 0.0152 | No |
| 2314 | ENSG00000232918 | AC118469.1 |  |  | 58242 | NaN | 0.0145 | No |
| 2315 | ENSG00000231562 | AL512593.1 |  |  | 58265 | NaN | 0.0141 | No |
| 2316 | ENSG00000240934 | AC066616.1 |  |  | 58268 | NaN | 0.0141 | No |
| 2317 | ENSG00000250940 | AC116562.3 |  |  | 58278 | NaN | 0.0139 | No |
| 2318 | ENSG00000214760 | RPL21P1 |  |  | 58291 | NaN | 0.0137 | No |
| 2319 | ENSG00000230406 | AC079834.1 |  |  | 58314 | NaN | 0.0134 | No |
| 2320 | ENSG00000230405 | RPS3AP52 |  |  | 58315 | NaN | 0.0134 | No |
| 2321 | ENSG00000233278 | RPS26P2 |  |  | 58336 | NaN | 0.0130 | No |
| 2322 | ENSG00000233189 | RPL12P29 |  |  | 58347 | NaN | 0.0129 | No |
| 2323 | ENSG00000224856 | AL035398.1 |  |  | 58357 | NaN | 0.0127 | No |
| 2324 | ENSG00000239689 | RPL17P46 |  |  | 58375 | NaN | 0.0124 | No |
| 2325 | ENSG00000244329 | UBA52P9 |  |  | 58433 | NaN | 0.0114 | No |
| 2326 | ENSG00000233287 | AC009362.1 |  |  | 58444 | NaN | 0.0113 | No |
| 2327 | ENSG00000282875 | BX322534.1 |  |  | 58498 | NaN | 0.0103 | No |
| 2328 | ENSG00000242058 | RPS4XP19 |  |  | 58507 | NaN | 0.0102 | No |
| 2329 | ENSG00000242052 | RPL10P7 |  |  | 58509 | NaN | 0.0102 | No |
| 2330 | ENSG00000218472 | AL590004.2 |  |  | 58534 | NaN | 0.0098 | No |
| 2331 | ENSG00000244043 | RPS27P27 |  |  | 58591 | NaN | 0.0088 | No |
| 2332 | ENSG00000232037 | AL590556.1 |  |  | 58624 | NaN | 0.0082 | No |
| 2333 | ENSG00000273155 | AC092587.1 |  |  | 58648 | NaN | 0.0078 | No |
| 2334 | ENSG00000241828 | AC006517.1 |  |  | 58654 | NaN | 0.0078 | No |
| 2335 | ENSG00000241035 | AL161747.1 |  |  | 58658 | NaN | 0.0077 | No |
| 2336 | ENSG00000233683 | AC004965.1 |  |  | 58684 | NaN | 0.0073 | No |
| 2337 | ENSG00000259873 | AC009117.1 |  |  | 58686 | NaN | 0.0073 | No |
| 2338 | ENSG00000214268 | RPS3AP33 |  |  | 58699 | NaN | 0.0071 | No |
| 2339 | ENSG00000217514 | AL031121.1 |  |  | 58708 | NaN | 0.0069 | No |
| 2340 | ENSG00000228071 | RPL7P47 |  |  | 58714 | NaN | 0.0069 | No |
| 2341 | ENSG00000232778 | RPL23AP50 |  |  | 58720 | NaN | 0.0068 | No |
| 2342 | ENSG00000234324 | RPL9P2 |  |  | 58763 | NaN | 0.0060 | No |
| 2343 | ENSG00000234325 | MRPS10P2 |  |  | 58764 | NaN | 0.0061 | No |
| 2344 | ENSG00000228646 | RPL31P13 |  |  | 58769 | NaN | 0.0060 | No |
| 2345 | ENSG00000244289 | RPS3AP35 |  |  | 58793 | NaN | 0.0056 | No |
| 2346 | ENSG00000244283 | RPL23AP73 |  |  | 58794 | NaN | 0.0056 | No |
| 2347 | ENSG00000226558 | AL590383.1 |  |  | 58807 | NaN | 0.0054 | No |
| 2348 | ENSG00000220553 | RPL5P19 |  |  | 58828 | NaN | 0.0051 | No |
| 2349 | ENSG00000226253 | MRPL35P3 |  |  | 58850 | NaN | 0.0047 | No |
| 2350 | ENSG00000242931 | RPL7P49 |  |  | 58858 | NaN | 0.0046 | No |
| 2351 | ENSG00000250306 | AC091826.1 |  |  | 58863 | NaN | 0.0045 | No |
| 2352 | ENSG00000231688 | RPL21P43 |  |  | 58882 | NaN | 0.0042 | No |
| 2353 | ENSG00000243711 | RPL21P116 |  |  | 58907 | NaN | 0.0038 | No |
| 2354 | ENSG00000241640 | AC092757.1 |  |  | 58924 | NaN | 0.0035 | No |
| 2355 | ENSG00000243050 | RPL30P10 |  |  | 58947 | NaN | 0.0031 | No |
| 2356 | ENSG00000271029 | AC135178.6 |  |  | 58971 | NaN | 0.0027 | No |
| 2357 | ENSG00000231922 | AL138479.1 |  |  | 59008 | NaN | 0.0021 | No |
| 2358 | ENSG00000234120 | AF228730.2 |  |  | 59033 | NaN | 0.0017 | No |
| 2359 | ENSG00000236191 | RPS15AP25 |  |  | 59098 | NaN | 0.0006 | No |
| 2360 | ENSG00000244422 | RPL38P3 |  |  | 59127 | NaN | 0.0001 | No |
| 2361 | ENSG00000240870 | RPL19P14 |  |  | 59132 | NaN | 0.0000 | No |
Table: GSEA details [plain text format]

  

Fig 2: RIBOSOME(HSA03010)      
 Blue-Pink O' Gram in the Space of the Analyzed GeneSet

  

Fig 3: RIBOSOME(HSA03010): Random ES distribution      
 Gene set null distribution of ES for **RIBOSOME(HSA03010)**
